# "I want to take the power back": An interpretative phenomenological analysis of self-management in coexisting physical and mental long-term conditions

**DOI:** 10.1371/journal.pone.0359157

**Published:** 2026-09-25

**Authors:** Catherine Lodwick, Leire Ambrosio, Mari Carmen Portillo

**Affiliations:** 1 School of Health Sciences, University of Southampton, Southampton, United Kingdom; 2 NIHR ARC Wessex, Southampton, United Kingdom; SKYDA Health Nigeria, NIGERIA

## Abstract

**Introduction:**

Individuals living with both physical and mental health conditions face compounding barriers to self-management. These challenges arise from complex care needs, interactions between long-term conditions symptoms and inequalities resulting in a high treatment burden. While previous research on coexisting conditions has largely focused on those with severe mental illness, there remains an important gap in understanding the factors that help or hinder self-management among adults living with coexisting physical and common mental health conditions (anxiety/depression) in the community.

**Objective:**

This study aims to explore the facilitators and barriers to self-management for adults living with a physical long-term condition and coexisting anxiety or depression in the community.

**Methods:**

A qualitative study with in-depth semi-structured interviews was conducted between May and August 2025. Eight adults living with coexisting physical and mental health conditions in the UK participated. Interview data was recorded, transcribed and analysed using Interpretative Phenomenological Analysis.

**Results:**

Three main themes were generated; (1) Moving beyond sink or swim: scaffolding support to self-manage (2) Self-managing: a choice or systemic exclusion (3) Unseen struggles: learning to live with my long-term conditions. Participants described the complex, often unsupported reality of self-managing coexisting long-term conditions, where many felt forced to integrate self-management into their lives due to systemic gaps and negative interactions with healthcare professional. Their experiences demonstrated an apparent hinderance to self-management, with age, condition visibility and identity struggles creating compounding barriers. Therefore, a holistic scaffolded support approach promoting integrated care was suggested to meet both their health and social needs.

**Conclusions:**

Our study provides insight into self-management facilitators and barriers among adults living with coexisting physical-mental health long-term conditions, highlighting substantial barriers to self-management and the need for person-centred, flexible, integrated support. The findings emphasise the need for new care models reflecting evolving presentations and domains of coexisting conditions.

## Introduction

In recent decades, the burden of living with long-term conditions (LTCs) has increased markedly. In the United Kingdom one in four adults, equating to 15 million people, live with at least two LTCs which are lifelong and cannot be cured [1]. Alongside this, NHS England and Improvement [2] estimate 30% of those living with LTCs have a coexisting mental health condition. It is plausible this figure has continued to rise, particularly as the World Health Organization [3] reported a 25% global prevalence increase in anxiety and depression following the COVID-19 pandemic thus demonstrating their expanding contribution to the global disease burden, highlighting the significant public health impact of coexisting physical and mental LTCs.

The growth in prevalence of coexisting conditions has far reaching implications not only for individuals but also their families and the wider health and social care systems [4]. In the UK, The Kings Fund [5] estimated the collective cost of coexisting physical and mental LTCs to NHS England to be more than £11 billion annually, with the Mental Health Foundation [6] finding a further £88 billion attributed to loss of productivity. Whilst there is currently no global consensus on the economic impact of coexisting LTCs, the World Health Organization [7,8] recognises that they significantly drive early mortality, raise perceived and actual treatment burden, and reduce productivity. All these factors have their own profound fiscal implications which drive long-term financial strain on national and global public services. Yet beyond the financial costs, living with coexisting physical and mental LTCs can be challenging for the individual as it creates complex, multidimensional care needs due to inequalities and condition interactions. Ultimately the interplay from coexisting conditions may cause poor care adherence, psychological distress and social isolation, which when combined lead to a significantly lower quality of life and higher healthcare utilisation compared to those living with a single LTC [9,10]. Notwithstanding, it is essential to highlight for this population not all care experiences are positive with many individuals reporting stigma, service fragmentation, and diagnostic overshadowing whereby a service users’ symptoms may be attributed to their mental health condition overlooking possible medical causes [11]. This disruption to care adversely impacts their wellbeing and trust in health and social care professionals [12].

In response to these challenges, there has been growing emphasis across clinical policies, practice and public health. This shift is most recently reflected in the NHS 10 Year plan, which is deeply focused on empowering individuals to be more active in managing their own health and wellbeing within community settings [1,13,14]. An important pillar of this plan focuses on facilitating the widespread adoption of digital tools, personalised care plans and self-management initiatives among service users and providers, which aligns directly with this study’s aim. Self-management refers to a combination of strategies from medication management, clinical treatments and behavioural changes to promote good health outcomes and improve one’s quality of life [15]. Although self-management is common amongst the general population, approaches used do not always account for the bidirectional relationship and specific needs of those living with coexisting physical and mental LTCs which produce a spectrum of challenges and contributes to widening health and social inequalities [16–18].

The World Health Organization define mental health as a state of mental wellbeing which enables individuals to cope with life stressors, learn and work well and contribute to their community [19]. Common mental ill health symptoms including low mood, anxiety, fatigue, and difficulties with motivation and concentration may reduce an individual’s capacity to self-manage day-to-day aspects of LTCs, including medication adherence, symptom monitoring, lifestyle modification, and engagement with healthcare services [20,21]. To manage these challenges alongside physical LTC, individuals may establish routines, use self-regulation and coping techniques, and draw on both informal support from friends and family and formal support from health and social care professionals [22]. Formal support for anxiety and depression may involve psychological therapies and pharmacological treatments; however, variability in their accessibility, continuity, and perceived effectiveness may further affect sustained self-management behaviours [23]. As recognised in the NHS Fit for the Future [14] plan, these obstacles highlight the need for integrated and person-centred care models which address both physical and mental health needs simultaneously, support continuity across services and recognise the interaction between coexisting LTCs.

Despite an increasing evidence-based on self-management interventions and support programmes for LTCs, the focus primarily remains on single-disease models, physical health conditions with clear biomedical markers and severe mental illnesses [24–26]. While Balogun-Katung et al. [27] and Coventry et al. [28] have begun to broadly examine barriers and enablers to self-managing coexisting LTCs, their research remains centred on those living with severe mental illness such as schizophrenia and bipolar disorder. In contrast, recent qualitative studies by Beaudin et al. [29] and Kappelin et al. [30] focus on common mental health conditions although from the perspective of healthcare professionals. This research illustrate that self-management support continues to be delivered in silos, despite acknowledging coexisting conditions, with practice continuing to show favour towards physical LTCs. Despite widespread recognition of the importance of integrated care in both international policies and guidelines, including The Big Mental Health Report [31] Major Conditions Strategy [1], UN Sustainable Development Goals [32], and WHO Policy on Mental Health [33], achieving meaningful integration remains a persistent challenge. These policies consistently emphasise the need to embed mental health support within physical health services, and vice versa, to deliver holistic, coordinated, and person-centred care.

The current care model does not capture coexisting mental LTCs, considering them as secondary concerns rather than integral components of LTC self-management. Therefore, the way living with coexisting LTCs impacts an individual’s systemic, relational and personal contexts are insufficiently understood [34–36]. As a result, there continues to be a limited awareness of how individuals self-manage the multidimensional and intersecting demands of coexisting physical LTCs and anxiety/depression. This qualitative study offers originality by aiming to explore facilitators and barriers to self-management among adults living with both a physical LTC and anxiety/depression in the community. By addressing this critical knowledge gap, the present study has the potential to inform the design of person-centred and contextually responsive self-management intervention, thereby, contributing to the reduction of health and social inequalities and enhancing the health and wellbeing outcomes for this underserved population.

## Materials and methods

### Study design

This was a qualitative study with interpretative phenomenological analysis (IPA) focused on the self-management experiences of adults living with coexisting physical LTCs and anxiety/depression. The IPA paradigm was deemed appropriate for this subject opposed to other broader qualitative inquiry approaches as its grounding in hermeneutic phenomenology aims to gain an in-depth understanding of an individual’s lived experience, alongside its idiographic nature, enabling rigorous interpretation on the meaning of an individual’s experience [37,38]. The interpretative stance of IPA facilitated a double hermeneutic process, an approach more congruent with the study’s phenomenological aim opposed to the systematic abstraction of grounded theory or observational immersion of ethnography, which was considered especially relevant for this study [39,40]. By promoting the researchers to deeply and systematically engage with participant’s realities and sense-making, this study’s design choice strengthens its trustworthiness [41,42]. To enhance the quality and rigor of results, the COREQ checklist created by Tong, Sainsbury, and Craig [43] was used and can be found in S1 Checklist.

### Sampling, study participants, and recruitment

Aligning with IPA practices, purposeful sampling was applied as it identifies individuals or groups with knowledge or experiences relevant to the phenomenon of interest; thus, this study focused on adults self-managing coexisting physical LTCs alongside anxiety and/or depression [44,45]. Participants were considered eligible to participate if they had a history of coexisting physical LTCs and anxiety and/or depression, lived in Southeast England, were aged 18 and over. Participants’ LTCs were self-diagnosed to enhance accessibility and reduce participation barriers, including limited feasibility and time to obtain or verify clinical information with healthcare providers. Those living in inpatient or residential settings were excluded as their self-management may be influenced or carried out by others. Sampling was congruent with the data analysis process and continued until no new ideas emerged, inferring information power had been achieved [46,47].

Recruitment for the study commenced on the 3^rd^ of May 2025 and concluded on the 31^st^ of July 2025 and occurred using three approaches. Firstly, recruitment posters were displayed in the Hampshire area. Secondly, social media channels (Instagram) advertised the research study and finally, two charitable organisations (Age UK, Communicare) were contacted, obtaining managerial approval, to publicise recruitment materials to members of their network. Potentially interested participants initially emailed the primary author using contact information provided on the recruitment poster. Upon expressing interest, participants completed a screen questionnaire to ensure eligibility. Where all criteria were met, participant information sheets and consent forms were sent to participants via email. Participants returned informed written consent forms, if they wished to proceed, via SafeSend software to ensure secure transfer of personal information. This was followed by the participant and the author arranging a mutually convenient interview date and time.

### Data collection

Participants were individually interviewed using a semi-structured format with open-ended questions, designed to encourage participants to provide a rich account of their lived experiences with minimal direction from the researcher. Following consultations with the PPIEP group and due to pragmatic considerations, interviews were conducted on Microsoft Teams which were audio and video recorded. The interview topic guide, found in S1 Table, was generated based on the existing literature and feedback from MCP and the study’s PPIEP group. All interviews were conducted between May and August 2025 and lasted between 40–70 minutes. Recordings were securely stored on a password protected computer in accordance with the Data Protection Act 2018 [48]. The researchers had no therapeutic relationship with study participants.

### Participation and Public Involvement, Engagement and Participation (PPIEP)

Two PPIEP members were consulted throughout all stages of the research process to ensure the study remained grounded in service user priorities with lived experience of coexisting LTCs, enhancing rigor and credibility. One member was a White mental health nurse living with anxiety, depression and several physical LTCs and the second member was a service user from the BAME (Black, Asian, and Minority Ethnic) community living with urinary and reproductive LTCs alongside anxiety and depression, which provided a range of knowledge and experiences. Meetings were held with the lead researcher (CL) and PPIEP members both in-person and online depending on the individual’s availability. Both PPIEP members contributed to the study from designing the scope and direction, topic guide development, theme naming and refinement. Key contributions from discussions between PPIEP members and CL included providing guidance on interview settings as discussions indicated that while in-person interviews may have further supported rapport-building, they were less feasible due to logistical constraints and the fluctuating nature of living with LTCs. As such, an online approach was chosen to accommodate participants’ varying needs and circumstances whilst allowing a more personable and interactive experience than telephone calls, which was crucial given the sensitive nature of discussing self-management experiences. Their extended contributions and engagement have been evaluated using the GRIPP2-SF as recommended by Staniszewska et al. [49] to ensure reporting transparency, see S2 Checklist.

### Data analysis

Each interview was transcribed verbatim with all personal identifying information removed and pseudonymised, with pseudonyms chosen by participants when arranging the interviews [50]. Data analysis began as soon as each interview was transcribed, employing the six step IPA process by Smith et al. [38]. No digital software was used to analyse the data as Smith et al. [38] encourage use of the paper-pencil method to promote closeness and deep engagement when analysing and critically interpreting the data.

Initially, the transcripts were printed and numbered with continuous lines. Transcripts were examined one-by-one to gain a clear picture of the individual’s lived self-management experience. The inductive analysis process began by creating exploratory notes focusing on three distinct categories: descriptive, linguistic, and conceptual, marked by corresponding coloured pens on the right-hand side of the margins. Descriptive comments focused on the participants’ accounts of key events, experiences, and emotions. Linguistic notes attended to the participants’ use of language, such as tone, repetition, pauses, metaphors, emphasis, or pronoun use. Conceptual codes involved a more interpretative level of engagement, exploring underlying assumptions, possible meanings and connections between participant’s experiences and broader psychological or experiential concepts. From the exploratory notes, experiential statements were constructed, written on the left-hand side of the margins, summarising the transcription as well as exploratory notes. This process involved synthesising descriptive, linguistic, and conceptual observations into concise interpretative statements which captured the lived meaning of participant’s experiences. The researcher then cut the transcript into these experiential statements, accompanied by the corresponding transcript lines and exploratory notes. These experiential statements were then laid out to visually facilitate a systematic exploration of connections, similarities, differences, and patterns across the data. Through this iterative process, related statements were clustered together to generate Personal Experiential Themes (PETs), capturing how individual participants understood and made meaning of their lived experiences. See S2 Table for further illustration of the process of developing exploratory notes, into experiential statements and subsequent themes.

This process was applied to all eight transcripts after which a summary document was produced for each participant’s PETs. Following refinement of all PETs, theme summaries were reorganised to enable comparison across participants and exploration of patterns and relationships between accounts. This iterative process informed the development of Group Experiential Themes (GETs), representing shared experiential meanings across participants, and were developed when a theme was present in at least half of the accounts. The findings of this study underwent a rigorous analysis and interpretation process, thus resulting in the creation of analytical themes which represent a synthesis of participant accounts, interpretative engagement, and theoretically informed understandings [51,52]. Consistent with the theoretical and interpretative underpinnings of IPA, the GETs were examined at increasing levels of abstraction through a theory-informed interpretative lens. The aim of this process was not to generate formal theory, but to deepen understanding of participants lived experience and position the findings in relation to existing scholarship [53,54]. To ensure analytical robustness, two of the authors (CL and MCP) corresponded monthly to discuss and revise the GETs and subthemes. Member checking was not employed as a validation strategy reflecting recent discourse questioning its suitability within IPA [55]. Instead, credibility was enhanced using peer debriefing, audit trails, and the inclusion of thick descriptions [41,54].

### Ethical considerations

Ethical approval was obtained from the University of Southampton Ethics Committee and the Research Integrity and Governance offices under the reference ERGO 102445, see S1 and S2 Fig. As this research study was unfunded, no financial or material compensation was provided to participants. All the research conforms to the ethical principles for medical research on human beings set out in the Declaration of Helsinki [56].

## Results

### Study participants

A total of ten potential participants contacted the researcher (CL). However, one did not complete the follow-up, and another withdrew at interview stage. Whilst the concept of information power influenced the overall sampling approach, only eight participants were interviewed and included in the final analysis of this study [46]. Despite this, the dataset was deemed sufficient to support meaningful analysis commensurate with IPA philosophy which prioritises depth of insights opposed to frequency to preserve the nuanced interpretation which IPA demands. The characteristics of included participants (Table 1) consisted of seven women and one man, aged between 18 and 34, with physical LTCs encompassing a scope of body systems from hepatic, gastrointestinal, reproductive, musculoskeletal and the nervous system. This small sample is common within IPA studies aligning with recommended ranges of four to ten participants, allowing for more in-depth interpretation as opposed to superficial understanding which may accompany a more diverse sample [57].

**Table 1:** Characteristics of interview participants.

Participant	Gender	Age	Physical LTCs	Mental LTCs
Rosemary	Female	25-34	Non-Alcoholic Fatty Liver Disease (NAFLD), Irritable Bowel Syndrome (IBS)	Anxiety and Depression
Grace	Female	18-24	Scoliosis, Irritable Bowel Syndrome (IBS)	Anxiety and Depression
Poppy	Female	18-24	Hypermobile Ehlers-Danlos Syndrome (hEDS), Chronic fatigue syndrome (CFS), Chronic migraines	Anxiety and Depression
Ruby	Female	18-24	Chronic constipation, Irritable Bowel Syndrome (IBS), Hypermobility	Anxiety and Depression
Oskar	Male	18-24	Gout	Anxiety
Ashley	Female	18-24	Hypermobile Ehlers-Danlos Syndrome (hEDS), Postural Orthostatic Tachycardia Syndrome (PoTS)	Anxiety and Depression
Laticia	Female	18-24	Irritable Bowel Syndrome (IBS)	Anxiety and Depression
Simone	Female	18-24	Endometriosis, Adenomyosis	Anxiety

Names included are pseudonyms to maintain anonymity.

### Themes and subthemes

The analysis process revealed high levels of convergence across the eight interviews which produced three Group Experiential Themes centered on the facilitators and barriers encountered when self-managing coexisting physical and mental LTCs. The themes alongside their associated sub-themes are detailed in Table 2. Furthermore, each participant’s contribution to the development of GETs is illustrated in S3 Table.

**Table 2:** Description of themes and subthemes.

Theme	Description of themes	Subthemes
Moving beyond sink or swim: scaffolding support to self-manage	Articulated the experiences of self-managing coexisting LTCs in the community, through balancing condition demands and calling for more holistic strategies.	“An internal battle”: living with coexisting conditions Seeing me, not just my long-term conditions
Self-managing: a choice or systemic exclusion	Focused on a critical exploration of structural barriers in the healthcare system for adults living with coexisting physical and mental LTCs, and how people responded by creating their own individualised self-management meaning.	“You just feel lost”: Navigating the healthcare system “Taking control” through self-management
Unseen struggles: learning to live with my long-term conditions	Centred on how participants felt their lived experiences were undermined, either due to age or the invisibility of LTCs leading to internal struggles regarding their identities.	My medical identity vs my social identity Self-managing now for my future

### Theme 1. Moving beyond sink or swim: Scaffolding support to self-manage

#### “An internal battle”: Living with coexisting conditions.

All participants described a deeply “interlinking” relationship between their physical and mental health LTCs, whereby one condition intensified the other’s symptoms often negatively. This bidirectional relationship made addressing coexisting LTCs simultaneously very challenging, frequently resulting in the prioritisation of one condition with remaining LTCs forced to take a “backseat”. For many, there was a continual shifting of priorities depending on the LTC causing the most prevalent concerns, repeatedly reported as pain or the external effects of mental health on friends and family. This fluidity contributed to a cycle of fatigue, emotional exhaustion, and frustration.

*“It’s like pushing a boulder up a hill and if I take my hand off that boulder to focus on my mental health and that boulder starts rolling, it’s just like, uh my God. Now we have to start all of this again” (Rosemary)*

Seven participants described managing their mental and physical LTCs concurrently as an ongoing challenge, referred to as an “internal battle” or “double edged blade”. All participants went on to note unlike their physical LTCs which they felt had clear, more tangible self-management strategies, mental health concerns were harder to address and slower to resolve.

*“I think, I think I personally prioritise my mental health over my physical health just because as long as I’m not like acutely unwell physically I can get back on top of my physical health but sometimes I know that if my mental health suffers it takes longer to get out of that kind of rut than it would if I was physically ill. Um so I do prioritise my mental health just because I know the consequences can be a bit more severe in terms of it I kind of slip on that end of things.” (Ashley)*

This lack of immediate identification and relief often led to reduced motivation to engage in self-management activities, feeding into a “downward spiral”. Overall, participants conveyed that the interplay between LTCs and constant renegotiation of self-management strategies had a significant emotional and physical impact on their daily lives.

*“You kind of just don’t want to bother with self-managing after that because you’re like what’s the point if it’s, if it’s not worked, what’s the point? And you sort of, sort of I don’t know how to describe it, it’s more, it’s like a pit you sort of just get stuck in this pit of what’s the point, because nothing works and I just have to deal with it but I don’t want to deal with it and so you’re just stuck in this like little downward spiral almost.” (Laticia)*

#### Seeing me, not just my long-term conditions.

Despite the complexity of managing coexisting conditions, participants reported a persistent expectation to adhere to standardised models of care. Grace and Simone described these experiences as dehumanising making them feel like “guinea pigs” in a system overly focused on medications. Further supported by a strong rejection of a “one-size-fits-all” approach with participants instead calling for tailored strategies that acknowledge both the individual and specific interplay of their LTCs.

*“Instead, quite often I’m faced with you need to do this and you need to do that and I’m like, ok, but we’re not really thinking about what does and doesn’t work. We’re kind of trying to put that one-size-fits-all thing.” (Rosemary)*

*“Yeah, the WRAP [Wellness Recovery Action Plan] planning for mental health, I think that was really good in making me identify what kind of like, asking those questions and putting it back to me to kind of tell. Making me kind of work out what helped, like facilitating me to work out what helped me. They, I think it’s a lot of like just communicating to me that they were hopeful for me to go and often live this like good life whilst self-managing. I think it was just their overall approach of being very like hopeful and realistic and listening to me about what worked and what didn’t work. And like when things weren’t going so well just involving me and making the decisions to do the things to tailor my treatment or like make a plan like very much involving me in the planning. Whereas like with the GP, they just made the plan and gave it to me.” (Ruby)*

Although recognised by all participants, this need was especially pronounced among Poppy, Ruby and Oskar who wanted healthcare professionals to acknowledge self-management “from the get go” of their chronic illness “journeys”, as they found receiving diagnoses and treatment planning to be overwhelming moments.

*“I think it wasn’t really, when I was getting my diagnosis it wasn’t really emphasised that how controllable this is. I think it’s more something that I’ve learned with time. Um, whereas like I think some reassurance from the start of hey look, this is like this is a very treatable thing, yeah, like, this is a very manageable problem like it would help with not panicking at the, at the start” (Oskar)*

Six participants emphasised the importance of early interventions and education on coexisting LTCs, including timely conversations post-diagnosis which explore personalised self-management strategies, reinforcing motivation and helping to establish individualised goals.

*“I think as a healthcare professional acknowledging that is so important, like talking about exercise, talking about hobbies, you know, Maslow’s hierarchy of needs what’s at the bottom part like what’s your purpose kind of thing I think is really important’ (Simone)*

A consensus among participants was that feeling heard and collaboration with healthcare providers when planning care are critical factors to long-term engagement. However, to achieve this goal there is a need to move away from the current perceived sink or swim model which places sole responsibility on the individual and their personal resources, to a collaborative approach where support is scaffolded over time.

*“It has just been a lot of throwing them in the deep end and hope that they can swim. It definitely has been an approach of that and so a lot of the stuff that you figure out, you either figure out on by yourself or using your own resources such as paying for counselling.” (Laticia)*

Although when planning care, Poppy stressed the value of practical over theoretical tools. This opinion was supported by Ruby, who together highlighted the importance of usable, realistic approaches tailored to the challenges of managing coexisting LTCs, although they hadn’t personally found one yet. However, Simone spoke of her “toolkit”, whereby self-management strategies are colour-coded green, orange or red to represent corresponding symptom severity. This approach was designed in collaboration with healthcare providers creating a tangible, effective self-management plan which aided her to “kill two birds with one stone” referring to her coexisting LTCs.

*“We’ve made a plan. In fact, we’ve made a plan, here’s our number at the bottom we might not get back to you for eight weeks but you know we’ve got it. I think that gives someone, that puts them in control of their outcomes and their own health and do you know what I mean, in like a more palatable way” (Simone)*

### Theme 2. Self-managing: A choice or systemic exclusion

#### “You just feel lost”: Navigating the healthcare system.

Whilst most participants wanted the healthcare system to move towards a “holistic route”, their current reality was often opposite. Many participants described damaged relationships with healthcare professionals characterised by feeling dismissed, ignored or disbelieved, which was regularly linked to diagnostic overshadowing.

*“I just think that when you go with a problem they should consider your whole, your whole story instead of, ‘cause I’ve not only had like, it’s not just. If I go there and say I’m, you know, I feel sick or whatever, um I think it’s quite easy for them to say, oh, it’s probably you’re probably anxious or whatever when really, well you should actually look into that, because I’ve also had surgery. So, you could be missing something else type thing, but, yeah, I don’t know, I just think they should consider the story as a whole and not. They should take more time before they come to a conclusion, I think” (Grace)*

Enduring these “constant experiences” contributed towards a resentment of healthcare providers among seven participants. These participants withdrew from formal care and turned towards self-management, not as a preference for LTC management but a necessity born out of advocacy fatigue.

*“So, it was kind of a it was a bit of a fight with my GP trying to just get like the testing done and, and push for the diagnosis just because then it did entitle me to like it just allowed me to have more understanding about myself.” (Ashley)*

Instead of traditional services, informal sources of support such as online communities, social media, and support groups were more popular. These spaces offered validation and understanding, regularly missing in clinical encounters, although Poppy noted the absence of professional healthcare involvement could foster uncertainty about the reliability of shared advice creating self-doubt in one’s self-management abilities.

*“Um I worry that like I’m possibly not doing the right things or there are things I’m missing, because I’m not having any input from people that really know what they’re talking about” (Poppy)*

Structural barriers within the healthcare system were considered by all participants. They highlighted poor communication, fragmented care pathways, complex referral processes, and geographical constraints as persistent obstacles to self-managing their coexisting LTCs. There was a clear need to streamline services and integrate care to create healthcare systems which are inclusive and responsive opposed to exclusionary and delayed.

#### “Taking control” through self-management.

Despite their common negative care experiences, all participants recounted a gradual shift from service reliance to personal agency in self-managing their LTCs. All participants recounted how the meaning of self-management had evolved into some form of “taking control”, whereby traditional clinical outcomes were replaced with personal definitions such as achieving a sense of normality, independence, happiness, productivity or simply basic functioning. As a method to achieve these goals, knowledge emerged as a key tool, with participants using a variety of strategies to build their understanding including peer learning, trial and error, formal education, and personal research.

*“I’ve had a lot of time for trial and error. I’ve had a lot of time to do different things, find what works for me and used like different tools to kind of find what works for me.” (Ruby)*

*“I think my mum looking into how to properly self-manage and all of that and she like gave me these like cliff notes basically that I could like look into myself because at the time where this information was like when my mum was looking into it, I was in the middle of a gout attack. I wasn’t looking up anything for myself. Yeah, that was actually the most helpful thing anyone’s ever done.” (Oskar)*

However, when gathering this information Rosemary and Ashley encountered difficulties accessing relevant condition specific information, especially when unwell which felt like a “backwards” process. Supported by Oskar and Laticia who recounted knowledge finding as an “energy consuming task” which was at odds with trying to meet their basic daily needs. Therefore, recommendations for a reliable “bank” of information from the healthcare systems were idealised to mitigate this barrier.

*“I understand that GPs are generalists, but it would be nice if they had, you know, sort of a bank. You know how, like GPs, they’ve always got leaflets or information sheets, it would be nice if those kind of existed about my condition that they could pass on instead of me being the one going out and doing all of this research” (Rosemary)*

Where this information was already available, through charity websites, Poppy expressed a strong desire for information to be presented from a holistic self-management lens rather than a purely biomedical perspective.

*“There’s some really good like guidance on Ehlers-Danlos, they have on their own website which has some really helpful stuff. But a lot of it, like when I look into it, it’s either really fundamental stuff or it’s not written in a perspective of self-management at all. It’s written in like these are the biological components of hypermobility. And I’m like, ugh, I don’t care” (Poppy)*

### Theme 3. Unseen struggles: Learning to live with my long-term conditions

#### My medical identity vs my social identity.

A key barrier to self-management expressed by all participants was how ageism and stigma significantly shaped their experiences of care, with their youth causing their concerns to be invalidated or second-guessed.

*“Ugh to be honest I just showed up one day, the woman looked at my foot and she said it looks like gout, but you’re young so do you have a family history?” (Oskar)*

Many felt age created a hierarchy of legitimacy where youth conflicted with perceptions of illness, leaving them to feel invisible or misunderstood. This tension was especially pronounced for Rosemary and Poppy, who revealed this feeling was further complicated by living with LTCs which lacked objective physical signs. The absence of visible symptoms appeared to undermine their credibility in social and clinical settings, causing them to “doubt” their level of illness and intensified feelings of exclusion.

*“Um, it just feels like everything that I’ve come up against is kind of you have to do it and you have to be the only person in it when that’s just not the way that I’m able to operate you know, like it’s if a person, I don’t know, needed a crutch or a walking stick, but for some reason the GP wants them to walk through the surgery without a walking stick that just feels a bit irritating.” (Rosemary)*

*“I just have this thought of, like, we are all just tired and like just go to uni and get a grip, and it’s not that big of a deal and actually it’s not normal and it’s, it’s not something everyone deals with um and trying to remember that because I can be really hard on myself and be like, why can’t I just push through? But actually, because it’s a medical condition, you wouldn’t tell someone with like asthma to push through an asthma attack like.” (Poppy)*

While there was a desire for greater recognition and visibility of younger people with less-represented LTCs, Simone feared being boxed in or stereotyped as a result.

*“I understand that there needs to be visibility with chronic pain conditions and the more we talk about it, but I did wonder about the stigma and the label of labelling myself as someone like if I presume this identity of someone with chronic pain what have I, have I actually put myself into a box where I can’t, do you know what I mean? It becomes part of your identity... I tried it and realised actually I’ve just minimised myself more as a person because now I’m just some you know [18-24]-year-old with chronic pain and not someone who likes going out.” (Simone)*

These experiences contributed to a blurring of identities: where medical needs became entangled with personal identity, overwhelming their sense of self. However, obtaining formal diagnoses was viewed as both gateways to care and burdensome, concurrently validating experiences whilst constraining identity. Balancing medical and social identities was particularly difficult for those in their early twenties who spoke about feeling torn when managing their health and maintaining friendships, relationships and a sense of normality. This created an internal conflict marked by sacrifice and loss as participants struggled to reconcile youth with chronic illness.

*"I didn’t want to be that like mentally ill friend that was only talking about mental her mental illness. That friend that was always like kind of dragging people down or like, oh, my mental health is rubbish today.” (Ashley)*

*“I love festivals and I love music and I think when my symptoms got out of control and I was like I actually don’t, I don’t know if I can do this and this is really upsetting.” (Simone)*

#### Self-managing now for my future.

Following the dilemma of being younger and living with LTCs, participants highlighted significant gaps in self-management resources tailored towards younger people. They found information about self-management to have a primary condition focus or be “catered” towards older generations, failing to reflect the realities of youth. There was clear frustration at the assumption of younger people being uninterested in self-managing, where in fact participants expressed strong motivation to learn and adopt strategies to maintain their health. Poppy, Oskar and Simone wanted “sustainable” solutions that allowed them to participate in social activities like clubbing, drinking or festivals without forfeiting their mental or physical wellbeing.

*“I also think like a lot of self-management, I see definitely tends to be catered towards like an older generation. Like, it’s like, oh you want to move more? Go out in the garden or play bowls...but the, the like sustainable lifestyle changes don’t often seem to accommodate for being younger and like your social groups looking different and being at university, or because I think they just assume that people aren’t trying to self-manage in those situations. And it, it makes it really difficult because it, it does like when I look into things, it’s like it just doesn’t feel like it’s catered to me and my life at all.” (Poppy)*

A key theme raised was the need for healthcare providers to recognise varying life stages alter how LTCs impact an individual’s life and therefore their self-management approach. Both Grace and Ruby recalled losing the ability to self-manage in childhood due to parental or institutional involvement. These events shaped their views of self-management in adulthood, with Grace wanting to see self-management as a choice not a discipline, whilst Ruby viewed it as a privilege after it was “taken out of my hands”.

*“So, it’s almost like I work harder to self-manage because I don’t want to experience that again but then also I feel like, it’s like for me part of it is having the power of being able to self-manage myself like it’s such because its something that I haven’t been able to do before, um or like it’s more been taken away from me. Because I wasn’t in a place to do that, it’s, I almost feel like I’ve got a bit of like, it’s a bit of a privilege to be able to do that myself.” (Ruby)*

A further barrier was the emotional and practical toll of self-managing as an emerging adult, having to account for future deterioration and LTC progression. Ruby and Poppy noted this outlook as “quite a hard thing to accept” but were resolved to incorporate self-management as a proactive measure to age well. Whereas Simone determined it was best for her to live in the present “I kind of just do what I want and then reap the consequences”.

*“I try my best to make choices that will help me age well, but there’s definitely some fear of like, what’s my future going to look life if things, things could get progressively worse. So, I have a component of me that’s that it’s quite scary and then there’s just also bits of me that just get annoyed. I’m like, what, for God’s sake [laughs], I don’t need to like, I don’t need to be thinking about this at [18-24] but like also I do. But you know, one of those.” (Poppy)*

Other key transitions of aging such as moving between school, university and work arose as vulnerable periods marked by gaps in support, embarrassment around disclosing health needs and uncertainty requesting reasonable adjustments. Ashley and Laticia discussed the lack of employer knowledge of coinciding conditions, and these were evident areas of improvement needed to foster self-management.

*“The services that you need to go to in your like workplace aren’t as obvious as well. Like I didn’t know about the basically what is like the DSA [Disabled Students’ Allowance] equivalent of working like and I’ve already forgotten it’s name, but like access to work, I think it’s yeah access to work. I didn’t know anything about that. No one told me. It’s that, that into that’s this not the seamless, what’s the word, that sort of movement on from university on from education into like working life. Like no one prepares you for that so you don’t know what you have available and even when you go looking, it’s not that obvious. Like they leave it quite up to you which don’t get me wrong is, is good in some parts but at the same time it’s I also don’t know because it’s my first time in this situation and I don’t know what I can ask for.” (Laticia)*

Despite these barriers, participants offered solution-focused suggestions such as digital profile packs detailing their LTCs and self-management needs to be sent to educational or occupational supervisors, streamlining advocacy processes and clearer support pathways. Their message was clear: young people are willing and ready to self-manage but they need systems whether that be healthcare, higher education or occupational that are designed to support their self-management goals, not assumptions overlooking them.

## Discussion

This qualitative study has explored the facilitators and barriers to self-management for adults living with coexisting physical and mental LTCs. Our findings add a new perspective to the literature by evidencing how developmental and life-stage factors may shape experiences of self-management and contribute to health and social inequalities. Previous studies exploring inequalities in multiple LTCs research have predominantly centred on socioeconomic status [58,59], education and health literacy [60–62] as primary determinants of disparity. While these factors remain important, our findings suggest that age and life-experiences may also heavily influence how individuals navigate the complexities of managing coexisting conditions. Although our sample was considered small and heavily weighted towards younger adults and women, which may have influenced the analysis and limits the transferability of the results, our findings suggest that young age represents an overlooked axis of inequality with emerging adults experiencing distinct, under-recognised challenges in self-management compared to other age groups. These findings contest dominant narratives found within current self-management frameworks, underscoring the need to reconsider how age-related developmental factors intersect with LTC management [63–65].

The findings from our study offer a conceptual leap by providing detailed accounts of participants shared, complex, and varied experiences of self-managing coexisting physical and mental LTCs. Participants recalled constant negative interactions with healthcare professionals and services which they attributed to either their age or the complexity of living with coexisting LTCs. Our data showed these experiences detrimentally impacted their trust and view of traditional healthcare services thus prompting a growing reliance on peer networks and social media platforms as alternative sources of self-management support, signalling a notable shift in both the locus and modality of self-management behaviours. Furthermore, the analysis indicated pronounced healthcare disparities owing to the visibility or invisibility of their coexisting conditions, which shaped how their care needs were recognised and responded to by others. Moreover, participants highlighted a lack of scaffolded self-management support specifically tailored to coexisting conditions, reflecting broader gaps within both healthcare provision and support resources regarding integrated self-management for concurrent mental and physical LTCs. Collectively, these findings demonstrate the critical reform required in both professional and societal understandings of living with and self-managing coexisting conditions to better support this population to live well. These insights must be integrated into policy and practice to help prevent the further widening of health and social inequalities.

Compared with previous literature, our research both aligns with and diverges from dominant perspectives of self-management [66–68]. Whilst existing literature has addressed aspects of systematic facilitators and barriers to self-management for adults living with coexisting LTCS [27,69,70], concurrent with our findings, few papers have explicitly isolated younger age as a central concept to health and social inequalities within the self-management of coexisting LTCs. Despite being routinely collected, age is seldom critically examined beyond the context of aging in self-management. As demonstrated in our results, young adults living with coexisting LTCs often experience stigma both from healthcare professionals and peers when discussing their LTCs, leading to experiences of self-doubt, resentment and feeling both medically and socially excluded. The youth-age dichotomy often constructs these life stages as oppositional, reinforcing assumptions of youth equating health, adaptability and resilience. However, this overlooks the profound self-management challenges faced by younger adults, which whilst different in nature are equally as significant as those faced by older populations [71,72]. Although the World Health Organization [8,73] recognises age as a social determinant of health, national and international guidelines [1,14,74] remain largely age-neutral, failing to account for the developmental and lifestyle factors which shape self-management experiences across the life course. While older and working-age adults are likely to experience distinct barriers to self-management [75], these challenges are more commonly represented within existing research and policy discourse, whereas the experiences of younger adults remain comparatively underexplored. Recent evidence, including the NHS Fit for the Future Plan [14] demonstrates that LTC prevalence is no longer driven solely by an aging population, with increasing numbers of working-age and young adults affected. Nonetheless, policy responses continue to prioritise older adults, framed through deficit-based narratives of cognitive decline or low health and digital literacy [76,77]. Whilst emerging initiatives, such as Young Future Hubs, remain limited in scope supporting only those under twenty-five and focusing primarily on mental health and social needs [78]. This leaves a significant care gap for emerging adults living with coexisting LTCs who require tailored and integrated support for their physical, mental, and social care needs. Our study challenges the prevailing assumption that younger age implies a lesser need or greater capacity for self-management. Such assumptions marginalise emerging adults living with coexisting LTCs whose health and social care needs may fall outside of normative expectations across health, academic and occupational settings. These findings indicate current care pathways must be adapted to better support young adults with coexisting LTCs through integrated, developmentally appropriate self-management strategies.

Additionally, our findings further showed participants who lived with non-visible LTCs such as hypermobility spectrum disorder, irritable bowel syndrome, or mental health conditions faced significant barriers to professional recognition and self-management support. Although non-visible chronic illnesses have gained increasing public recognition in recent years, for example through initiatives such as the sunflower lanyard [79], individuals with non-visible LTCs continue to experience stigma and challenges to illness legitimacy in both medical and social settings [80]. Participants attributed these difficulties to care professionals’ prioritisation of visible or objective indicators such as appearance, use of mobility aids, and abnormal diagnostic markers over lived experience and reported impacts on quality of life. This lack of recognition contributed to feelings of exclusion and stigma [12,81] reinforcing barriers to accessing care and wider support, including reasonable adjustments in education and employment [82,83]. Requiring individuals to prove the credibility of their ill health exemplifies epistemic injustice, distorts clinical encounters and undermines the therapeutic relationships essential to effective practice. These obstacles were compounded by self-management interventions narrowly tailored towards physical LTCs with clear biomedical markers and prescribed behaviours such as exercise, diet, and medication management [84,85]. Consequently, this focus marginalises individuals with invisible, fluctuating or complex LTCs, particularly those with coexisting mental health conditions. Moreover, the limited or superficial integration of mental health support was misaligned with participants lived experiences, reducing engagement and intervention effectiveness. Participants reported current interventions failed to accommodate their coexisting LTCs often forcing them to prioritise one over another, despite multiple LTCs increasingly reflected in routine clinical practice. Without embedding mental health as a core component of LTC management, rather than an adjunct, adults with coexisting LTCs will continue to experience unmet needs, poorer health outcomes, and inequitable access to support [86–88]. To prevent further widening of health inequalities, self-management interventions must recognise invisible LTCs, incorporate subjective symptoms, and embed mental health as a foundational element.

Our findings illustrated that participants perceived a lack of tailored self-management support. Existing strategies were experienced as biomedical in focus; catered towards older adults; and insufficiently inclusive of different lifestyles. This was evidenced within the literature by a paucity of self-management frameworks targeted towards both emerging adults and those with coexisting physical-mental LTCs. Dominant care models underpinning self-management interventions such as the Chronic Care Model [89] and Bandura’s self-efficacy theory [90], have limited applicability for these groups given their focus on single physical conditions and their presupposition for a level of autonomy, cognitive maturity, and life stability [91]. As such, participants described uncertainty about managing their LTCs cohesively without compromising social participation and relationships, which they identified as essential to maintaining mental wellbeing. In contrast, Paterson’s Shifting Perspective Model of Chronic Illness [92], which conceptualises chronic illness as a dynamic and evolving experience across the life course, offers a more appropriate framework for coexisting LTC self-management. The recognition that individuals move between periods of illness and wellness resonated strongly with participant narratives. Although our findings extend this model by suggesting that illness and wellbeing may coexist simultaneously rather than as alternating states. This reframing underlines the need for sustainable self-management resources, which enable individuals to prioritise wellbeing and social participation even during periods of illness. Notwithstanding, to avoid exacerbating health and social inequalities, the design of such resources must account for socioeconomic and cultural contexts as these factors have a moderating effect on self-management support [93,94]. Overall, our findings illustrate a conceptual leap for new self-management frameworks which consider managing coexisting LTC as a developmental process evolving alongside identity formation and illness trajectories.

Our findings provide an original contribution to this field of research by emphasising the complex interplay between age, identity and system navigation, offering a new conceptual lens to understand the facilitators and barriers to self-management of coexisting physical and mental LTCs. Rather than acting as a standalone determinant, age appears to interact with broader structural and individual factors, including identity formation and service organisation, shaping how individuals experience and engage with self-management practices. The accounts of emerging adults suggest that developmental context may be relevant to understand these processes across adulthood. Overall, the results emphasise the importance of developing age-sensitive self-management strategies, improving the visibility of coexisting LTCs within care provision, and ensuring mental health is recognised as a central and integral component of self-management alongside physical health needs.

### Implications for practice, policy and research

#### Clinical practice and policy.

Our study stresses the need to create age-sensitive self-management frameworks reflecting the distinctive biopsychosocial domains within young adulthood. As traditionally self-management models of care have focused on compliance and structured interventions tailored towards older generations which may be less effective for individuals still forming their identity and autonomy. Therefore, our findings offer a rationale for policymakers and practice providers to co-create a self-management framework which incorporates age-related barriers to engagement and explicitly embeds mental health support as a core component. Incorporating the priories and lived experiences of younger adults into clinical guidelines may enhance sustained engagement in both short and long-term self-management behaviours.

#### Future research.

Future research is warranted to examine how young adulthood and intersecting sociological factors such as social media use, peer support, and unstructured lifestyles shape self-management intervention uptake. Moreover, quantitative expansion of this work could employ longitudinal surveys to systematically examine how key facilitators (social support, technology, integrated care) and barriers (lack of tailored guidance, care discontinuity, social stigma) influence the development and evolution of self-management behaviours across the life course in adults living with coexisting physical-mental LTCs. A crucial element of this approach would be ensuring maximum variation in sampling to achieve representation across key demographic variables such as age, gender, socioeconomic status and types of LTC combination alongside differing levels of digital engagement and healthcare access. This would enable more robust comparative analyses across groups and strengthen the validity and transferability of findings. Based upon the results, there is potential to codesign targeted self-management interventions that integrate both medical and social needs, ensuring relevance and effectiveness for this underserved group.

### Strengths and limitations

A key strength of this study lies in its use of Interpretative Phenomenological Analysis which enables in-depth exploration of participants’ lived experiences. Although the small sample size, consistent with IPA’s idiographic focus, may have limited the breadth of experiences captured; the recurrence of similar themes suggests a degree of convergence and robustness. Peer debriefing between two authors further strengthened the analytical process and supported the credibility of the findings. However, there were several limitations within this study to note. Firstly, the recruitment strategy relied heavily on digital access and skills, which may have excluded underserved or older populations. Secondly, the researchers did not formally track which recruitment methods yielded participants, limiting the ability to evaluate the effectiveness of recruitment streams and inform future research. Thirdly, the sample size was relatively small and skewed towards female participants and younger adults, potentially reflecting self-selection bias and limiting transferability of the results. Fourth, as diagnostic status of mental health conditions was not verified the study relied on participants’ self-identification. Consequently, participants may have had either a formally diagnosed condition or a self-diagnosed condition which may have introduced variability in the nature and severity of reported experiences. Finally, the absence of socioeconomic, educational, and employment data represents a key limitation, as collecting these demographic factors could have enabled a more nuanced interpretation of the findings.

## Conclusion

This qualitative study examined the facilitators and barriers to self-management for adults living with physical LTCs and coexisting anxiety/depression. Overall, the study contributes to a more nuanced understanding of the lived experience of managing coexisting physical and mental LTCs, emphasising the need for person-centred, flexible, and age-appropriate responses to self-management support. While our study centred on the voices of emerging adults, the study provides a foundation for further research comparing self-management experiences across different age groups of adults living with coexisting conditions. Such work would support the development of life-course informed approaches to self-management that better reflect the developmental context and evolving needs of individuals across adulthood, which may inform the design of more tailored and effective self-management strategies.

## Supporting information

S1 Checklist Consolidated Criteria for reporting qualitative studies (COREQ): 32-item checklist. (DOCX)

S2 Checklist GRIPP2-SF Reporting Checklist. (DOCX)

S1 Fig Ethical approval. (TIF)

S2 Fig Ethical approval. (TIF)

S1 Table Interview topic guide. (DOCX)

S2 Table Stages of Interpretative Phenomenological analysis (extracts from Rosemary’s transcript). (DOCX)

S3 Table Participant contribution to Group Experiential Themes (GETs). (DOCX)
