# Supplementary material for: "I want to take the power back": An interpretative phenomenological analysis of self-management in coexisting physical and mental long-term conditions
Source: PLoS One. 2026 Sep 25;21(9):e0359157. doi: 10.1371/journal.pone.0359157 (PMC13614564; doi:10.1371/journal.pone.0359157)
Supplement: Supplementary file 2 [file pone.0359157.s010.pdf]

PLOS One

"I want to take the power back": An interpretative phenomenological analysis of self-management in coexisting physical and mental long-term conditions

--Manuscript Draft--

**Manuscript Number:**	PONE-D-26-09215R1
**Article Type:**	Research Article
**Full Title:**	"I want to take the power back": An interpretative phenomenological analysis of self-management in coexisting physical and mental long-term conditions
**Short Title:**	"I want to take the power back": Self-management in coexisting physical and mental long-term conditions
**Corresponding Author:**	Catherine Elizabeth Lodwick, MNurs, MRes<br>University of Southampton<br>Southampton, Hampshire UNITED KINGDOM OF GREAT BRITAIN AND NORTHERN IRELAND
**Keywords:**	self-management; long-term conditions; mental health; Inequality
**Abstract:**	Introduction: Individuals living with both physical and mental health conditions face compounding barriers to self-management. These challenges arise from complex care needs, interactions between long-term conditions symptoms and inequalities resulting in a high treatment burden. While previous research on coexisting conditions has largely focused on those with severe mental illness, there remains an important gap in understanding the factors that help or hinder self-management among adults living with coexisting physical and common mental health conditions (anxiety/depression) in the community.<br><br>Objective: This study aims to explore the facilitators and barriers to self-management for adults living with a physical long-term condition and coexisting anxiety or depression in the community.<br><br>Methods: A qualitative study with in-depth semi-structured interviews was conducted between May and August 2025. Eight adults living with coexisting physical and mental health conditions in the UK participated. Interview data was recorded, transcribed and analysed using Interpretative Phenomenological Analysis.<br><br>Results: Three main themes were generated; (1) Moving beyond sink or swim: scaffolding support to self-manage (2) Self-managing: a choice or systemic exclusion (3) Unseen struggles: learning to live with my long-term conditions. Participants described the complex, often unsupported reality of self-managing coexisting long-term conditions, where many felt forced to integrate self-management into their lives due to systemic gaps and negative interactions with healthcare professional. Their experiences demonstrated an apparent hinderance to self-management, with age, condition visibility and identity struggles creating compounding barriers. Therefore, a holistic scaffolded support approach promoting integrated care was suggested to meet both their health and social needs.<br><br>Conclusions: Our study provides insight into self-management facilitators and barriers among adults living with coexisting physical-mental health long-term conditions, highlighting substantial barriers to self-management and the need for person-centred, flexible, integrated support. The findings emphasise theneed for new care models reflecting evolving presentations and domains of coexisting conditions.
**Order of Authors:**	Catherine Lodwick
	Leire Ambrosio
	Mari Carmen Portillo
**Response to Reviewers:**	Comments from Editor<br><br>Comment 1: Funding statement – please add funding/grant number.

Powered by Editorial Manager® and ProduXion Manager® from Aries Systems Corporation

Response: We thank the Editor for this comment. We have updated the Funding Statement to include the relevant funding source and grant number. The grant: NIHR304046 – Inspiring Students into Research (R1 2023) was awarded to the lead researcher (CL) which covered tuition fees and a stipend. This funding was not allocated to the specific research study.

Comment 2: Sample size is relatively small and heavily skewed (Male 1, female 8). This should be factored in the discussion and perhaps presented as part of study weakness.

Response: We thank the Editor for highlighting this issue. We agree that the sample size and gender imbalance are limitations of the study. This has now been acknowledged in the revised discussion section (Page 29, Lines 652-654) and included explicitly as a study limitation (Page 36, Lines 819-820, 828-830).

Comment 3: Socioeconomic, education/employment status are missing. These are critical determinants of self-management and inequality. Please attribute this to study weakness

Response: We thank the Editor for this important observation. We agree that socioeconomic status, education and employment are key determinants of self-management and health inequality. These variables were not collected in this study, and this limitation has now been explicitly stated as a study weakness (Page 36, Lines 830-833).

Comment 4: It is unclear how interpretations moved from codes to themes and to theory. Please elaborate on this.

Response: We thank the Editor for this helpful comment. We have now elaborated on the analytic process to clarify how interpretations developed from initial coding through to theme development. In line with an Interpretative Phenomenological Analysis (IPA) approach, analysis involved close iterative, in-depth engagement with each transcript, beginning with detailed exploratory notes (descriptive, linguistic, and conceptual). These exploratory notes then informed the development of experiential statements, which were subsequently clustered into Personal Experiential Themes (PETs) for each participant. Cross-case analysis then enabled the development of Group Experiential Themes (GETs), which represent shared patterns of meaning across participants. We also clarify that IPA is not a theory-generating methodology, but an approach aiming at producing an interpretative account of lived experiences. This clarification has been added to the Methods section (Pages 11-13, Lines 260-298).

Comments from Reviewer 1

Comment 1: Line 177 - please resolve or complete this sentence to make it easy for readers to understand. e.g. thus for this study, adults self-managing coexisting physical LTCs with anxiety and/or depression was the phenomenon of interest/focus.

Response: We thank the reviewer for this comment. We have revised the sentence to improve clarity and readability, specifying that the phenomenon of interest in this study was adults self-managing coexisting physical long-term conditions alongside anxiety and/or depression (Page 8, Lines 194-195).

Powered by Editorial Manager® and ProduXion Manager® from Aries Systems Corporation

Comment 2: Line 178 - had a history or previously diagnosed with coexisting...

Response: We thank the reviewer for pointing this out. We have revised the wording for clarity and grammatical precision. This has been updated in the manuscript (Page 8-9, Lines 195-198).

Comment 3: Line 186 - Was the recruitment post disseminated via social media channels? which of the channels of dissemination yielded the highest number of participants?

Response: We thank the reviewer for this important point. The recruitment poster was disseminated via social media, and we have now specified the platform (Instagram) in the revised Methods section (Page 9, Line 206). However, recruitment sources were not systematically recorded by dissemination channels, as such it is not possible to determine which platform yielded the highest number of participants. This has been noted as a procedural limitation in the manuscript (Page 36, Lines 825-828).

Comment 4: Line 193 – sent out how? What platform/channel of transmission?

Response: We thank the reviewer for this comment. Participant consent forms were distributed to participants via email and once completed, participants returned them electronically via a secure online platform called SafeSend, prior to participation in the study. We have now clarified the consent procedure in the revised Methods section (Page 9, Lines 212-215).

Comment 5: Line 205 - Is there a reason for the inconsistency in dates? earlier you mentioned 3rd May - 31st July. Although you used the word between, but if possible, main the month of July for the sake of consistency.

Response: We thank the reviewer for pointing this out. Recruitment of participants took place between May and July, with a small number of interviews conducted in August due to participant availability and scheduling constraints. This provides clarification regarding the apparent inconsistency in the reporting.

Comment 6: Line 217 - Meetings with who? or between who?

Response: We thank this reviewer for this helpful comment. The meetings were held between the lead researcher (CL) and members of the PPIEP group. We have clarified the wording in the revised manuscript to avoid ambiguity (Page 10, Lines 238).

Comment 7: Any reference to support this or was it just a result from the discussions between PPIEP members?

Response: We thank the reviewer for this helpful comment. The changes to study design were informed by structured discussions with members of the PPIEP group, rather than a specific external reference. This has been clarified within the revised Methods section (Page 10, Lines 241-242).

Comment 8: Line 232 - what was chosen by the participants? the pseudo-names?

Response: We thank the reviewer for this clarification comment. This refers to

Powered by Editorial Manager® and ProduXion Manager® from Aries Systems Corporation

participants selecting their own pseudonyms to support anonymity. The manuscript has been revised to make this explicit (Page 11, Lines 253-255).

Comment 9: Thanks for including this. I was interested in learning why your study did not include any participant 35 years and above, and also why you had more females than males.

Response: We thank the reviewer for this insightful comment. The age range and female to male distribution reflect the characteristics of the eligible population who responded to the recruitment strategy and met the study inclusion criteria. No upper age limit of 35 years was set, and the sample composition may reflect self-selection bias. This has now been acknowledged in the Discussion section as a study limitation (Page 36, Lines 828-830).

Comment 10: On another note, thank you for suggesting future possible research. Would the authors consider incorporating strategies to capture a more representative demographic sample? This might allow for comparisons across age groups and gender, where appropriate. However, I recognize that such an expansion could become a separate research inquiry on its own.

Response: We thank the reviewer for this thoughtful suggestion. We agree that future research could benefit from recruiting a more demographically representative sample to facilitate comparisons across characteristics such as age and gender, where appropriate. In response to this comment, we have incorporated this recommendation into the Future Research section (Page 35, Lines 807-812), noting that future studies could employ a maximum variation sampling strategy during recruitment to capture a broader range of demographic and experiential characteristics.

Comments from Reviewer 2

Comment 1: I felt the description of mental health conditions could be thicker. Which mental health symptoms specifically affect self-management? How do they manage their mental health conditions? Does psycho-/pharmacotherapy help?

Response: We thank the reviewer for this insightful comment and respond to the points raised below:

Mental health symptoms: We agree that providing a more detailed description of mental health symptomology strengthens the manuscript. The Introduction has been expanded to specify key symptoms (e.g. low mood, anxiety, fatigue, and reduced motivation) that may affect an individuals' capacity to self-manage alongside an added definition of mental health from the World Health Organization (2025) (Pages 5-6, Lines 125-131)

Self-managing mental health conditions: We have further elaborated in the Introduction section on how individuals may self-manage mental health conditions, highlighting the complexity of managing coexisting physical and mental health conditions (Page 6, Lines 131-134; 137-141).

Psycho/pharmacotherapy: We have also expanded the Introduction to reflect the role of psychological and pharmacological therapies in supporting self-management, while acknowledging that their effectiveness and accessibility may vary between individuals (Page 6, Lines 134-137). These additions strengthen the conceptual framing of mental health within the manuscript.

Comment 2: I felt it a bit odd that Discussion focuses on age a lot, even though it does

Powered by Editorial Manager® and ProduXion Manager® from Aries Systems Corporation

	not come up in the Introduction, Methods, or the earlier parts of the Results. Though the authors do mention the potential unintentional exclusion of older populations, it would be better to discuss this earlier and more extensively.<br><br>Response: We thank the reviewer for this thoughtful comment. We acknowledge that the emphasis on age in the Discussion may have appeared disproportionate given its more limited framing in earlier sections of the manuscript. This reflects the fact that the study was not designed with age as a primary focus, and therefore younger age was not foregrounded in the earlier sections of the manuscript.<br><br>However, the final sample comprised only younger adults, which may reflect limitations in the recruitment process. This became a defining feature of the dataset, and in turn, shaped the interpretation of the findings. We therefore considered this an important and unanticipated outcome, particularly given this represents a significant gap in the existing literature, as younger adults with coexisting physical and mental health long-term conditions remain underrepresented in self-management research. As such the prominence of younger participants with coexisting physical and mental health long-term conditions is thus presented as a novel contribution arising from the data rather than an a priori focus of the study.<br><br>In response to the reviewer's comment, we have revised the Discussion section to improve balance across themes, while retaining appropriate emphasis on age as a key contextual and novel feature of the findings. We believe these revisions improve the coherence of the manuscript (Pages 29-34, Lines 643-659; 660-679, 697-701; 774-785).<br><br><br>Comment 3: Similarly, I feel that the conclusion of this manuscript ("The study found younger age to be a central inequality within self-management support, which crucially must be addressed…") may be too strong given that older populations were not included in the study. While it is true that young people face unique barriers as described in the manuscript, they probably also have advantages older people do not possess. A more balanced interpretation might be more appropriate.<br><br>Response: We thank the reviewer for this important comment. We agree that the original concluding statement was stronger than warranted given the study did not include participants from older age groups and therefore cannot support direct comparisons across the life course. In response, we have revised the Conclusion in both the manuscript (Page 37, Lines 834-846) and abstract (Page 3, Lines 57-62) to provide a more balanced interpretation of the findings. The revised Conclusion now emphasises that, while our findings highlight age-related factors may influence self-management among emerging adults living with coexisting physical and mental health long-term conditions, further research is needed to examine how these experiences may differ across age groups. This revision better reflects the scope of the data while retaining the novel contribution of the study.<br><br><br>Comment 4: The provided link for data seems inaccessible (it says "DOI Not Found"). Please double check.<br><br>Response: We thank the reviewer for highlighting the issue. We have checked the link and confirm that the DOI is not currently resolving. A DOI has been reserved for this dataset, which is present in the updated Data Availability Statement, and will be formally minted by the University of Southampton Data Management Team upon acceptance of the manuscript. The DOI will resolve to the below URL once active.<br><br>The dataset has now been made publicly accessible via the University of Southampton repository. This dataset can be accessed at the following URL: https://eprints.soton.ac.uk/512196/<br><br>To provide further clarification, we have included a letter from the University's Research Data Management team within the submission materials outlining this

	process. This ensures transparency regarding the status of the DOI and the associated data governance procedures.<br><br>Comment 5: Did the participants receive any compensation?<br><br>Response: We thank the reviewer for this question. The research study activities were unfunded, and as such participants did not receive any financial or material compensation for their participation. This has now been clearly stated in the revised Methods section of the manuscript (Page 13, Lines 307-308).<br><br>Comment 6: Line 109 - Explanation on "diagnostic overshadowing" might be beneficial for some readers<br><br>Response: We thank the reviewer for this helpful suggestion. We agree that a brief explanation of "diagnostic overshadowing" would improve clarity for readers. We have therefore added a concise definition in the revised manuscript (Page 5, Lines 107-108), describing diagnostic overshadowing as the tendency for symptoms of physical health conditions to be misattributed to an existing mental health diagnosis, potentially leading to under-recognition or delayed diagnosis of physical illness.<br><br>Comment 7: Line 178 - What criteria were used for anxiety/depression? Who diagnosed?<br><br>Response: We thank the reviewer for this important comment. Eligibility for anxiety and/or depression was based on participants' self-reported diagnosis. This approach was used to enhance accessibility and inclusivity, recognising variability in access to formal diagnostic services and differences in diagnostic recording within NHS pathways. This has now been clarified in the revised Methods section (Page 9, Lines 198-200).<br><br>Comment 8: Line 585 – What is DSA?<br><br>Response: We thank the reviewer for this clarification comment. We have now expanded the acronym of DSA in brackets next to the quotation (Page 28, Lines 624-625). DSA refers to Disabled Students' Allowance, a UK government-funded support scheme to help students in higher education who have a disability, long-term health condition, mental health condition, or specific learning difficulty. It is not a cash payment to the student, rather it provides funding for extra study-related support and equipment.<br><br>Additional Clarifications<br><br>In addition to the above comments, all spelling and grammatical errors pointed out by the reviewers have been corrected.<br><br>We look forward to hearing from you regarding our submission and to respond to any further questions and comments you may have.
**Additional Information:**	
**Question**	**Response**
**Competing Interests**<br><br>Use the instructions below to enter a competing interest statement for this	The authors have declared that no competing interests exist.

submission. On behalf of all authors, disclose any competing interests that could be perceived to bias this work—acknowledging all financial support and any other relevant financial or non-financial competing interests.<br><br>This statement is required for submission and **will appear in the published article** if the submission is accepted. Please make sure it is accurate and that any funding sources listed in your Funding Information later in the submission form are also declared in your Financial Disclosure statement.<br><br>View published research articles from *PLOS ONE* for specific examples.<br><br>**NO authors have competing interests**<br><br>Enter: *The authors have declared that no competing interests exist.*<br><br>**Authors with competing interests**<br><br>Enter competing interest details beginning with this statement:<br><br>*I have read the journal's policy and the authors of this manuscript have the following competing interests: [insert competing interests here]*<br><br>* typeset	
**Data Availability**<br><br>Authors are required to make all data underlying the findings described fully available, without restriction, and from the time of publication. PLOS allows rare exceptions to address legal and ethical concerns. See the PLOS Data Policy and FAQ for detailed information.<br><br>A Data Availability Statement describing where the data can be found is required at	Yes - all data are fully available without restriction

Powered by Editorial Manager® and ProduXion Manager® from Aries Systems Corporation

submission. Your answers to this question constitute the Data Availability Statement and **will be published in the article**, if accepted.<br><br>**Important:** Stating 'data available on request from the author' is not sufficient. If your data are only available upon request, select 'No' for the first question and explain your exceptional situation in the text box.<br><br>Do the authors confirm that all data underlying the findings described in their manuscript are fully available without restriction?	
Additional data availability information:	
**Financial Disclosure**<br><br>Enter a financial disclosure statement that describes the sources of funding for the work included in this submission. Review the submission guidelines for detailed requirements. View published research articles from *PLOS ONE* for specific examples.<br><br>This statement is required for submission and **will appear in the published article** if the submission is accepted. Please make sure it is accurate.<br><br>**Funded studies**<br>Enter a statement with the following details:<br>• Initials of the authors who received each award<br>• Grant numbers awarded to each author<br>• The full name of each funder<br>• URL of each funder website<br>• Did the sponsors or funders play any role in the study design, data collection and analysis, decision to publish, or preparation of the manuscript?<br><br>Did you receive funding for this work?	Yes
Please add funding details.<br>as follow-up to "**Financial Disclosure**	Catherine Lodwick was the recipient of the following National Institute for Health and Care Research award: NIHR304046 – Inspiring Students into Research (R1 2023). The views expressed in this publication are those of the authors and not necessarily

Enter a financial disclosure statement that describes the sources of funding for the work included in this submission. Review the submission guidelines for detailed requirements. View published research articles from *PLOS ONE* for specific examples.<br><br>This statement is required for submission and **will appear in the published article** if the submission is accepted. Please make sure it is accurate.<br><br>**Funded studies**<br>Enter a statement with the following details:<br>• Initials of the authors who received each award<br>• Grant numbers awarded to each author<br>• The full name of each funder<br>• URL of each funder website<br>• Did the sponsors or funders play any role in the study design, data collection and analysis, decision to publish, or preparation of the manuscript?<br><br>Did you receive funding for this work?"	those of the NIHR, NHS or the UK Department of Health and Social Care. The funders had no role in study design, data collection and analysis, decision to publish, or preparation of the manuscript.
Please select the country of your main research funder (please select carefully as in some cases this is used in fee calculation).<br>as follow-up to "**Financial Disclosure**<br><br>Enter a financial disclosure statement that describes the sources of funding for the work included in this submission. Review the submission guidelines for detailed requirements. View published research articles from *PLOS ONE* for specific examples.<br><br>This statement is required for submission and **will appear in the published article** if the submission is accepted. Please make sure it is accurate.	UNITED KINGDOM - GB

**Funded studies**<br>Enter a statement with the following details:<br>• Initials of the authors who received each award<br>• Grant numbers awarded to each author<br>• The full name of each funder<br>• URL of each funder website<br>• Did the sponsors or funders play any role in the study design, data collection and analysis, decision to publish, or preparation of the manuscript?<br><br>Did you receive funding for this work?"	
**Ethics Statement**<br><br>Enter an ethics statement for this submission. This statement is required if the study involved:<br><br>• Human participants<br>• Human specimens or tissue<br>• Vertebrate animals or cephalopods<br>• Vertebrate embryos or tissues<br>• Field research<br><br>Write "N/A" if the submission does not require an ethics statement.<br><br>General guidance is provided below. Consult the submission guidelines for detailed instructions. **Make sure that all information entered here is included in the Methods section of the manuscript.**	The study was reviewed and approved by the University of Southampton Ethics Committee and the Research Integrity and Governance Offices (reference number ERGO: 102445). Written informed consent was obtained from all participants.

**Format for specific study types**<br><br>**Human Subject Research (involving human participants and/or tissue)**<br>• Give the name of the institutional review board or ethics committee that approved the study<br>• Include the approval number and/or a statement indicating approval of this research<br>• Indicate the form of consent obtained (written/oral) or the reason that consent was not obtained (e.g. the data were analyzed anonymously)<br><br>**Animal Research (involving vertebrate animals, embryos or tissues)**<br>• Provide the name of the Institutional Animal Care and Use Committee (IACUC) or other relevant ethics board that reviewed the study protocol, and indicate whether they approved this research or granted a formal waiver of ethical approval<br>• Include an approval number if one was obtained<br>• If the study involved *non-human primates*, add *additional details* about animal welfare and steps taken to ameliorate suffering<br>• If anesthesia, euthanasia, or any kind of animal sacrifice is part of the study, include briefly which substances and/or methods were applied<br><br>**Field Research**<br><br>Include the following details if this study involves the collection of plant, animal, or other materials from a natural setting:<br>• Field permit number<br>• Name of the institution or relevant body that granted permission	
**Data Availability**<br><br>Before publication, Authors are required to make fully available and without restriction all data underlying their findings. Sharing the data underlying your findings increases the visibility, credibility, and impact of your work, while supporting transparency and future scientific discovery. Please see our PLOS Data	The underlying dataset supporting the findings of this study are openly available in the University of Southampton Institutional Repository at https://doi.org/10.5258/SOTON/D3861

Policy page for detailed information on this policy.<br><br>A **Data Availability Statement**, detailing where the data can be accessed, is required at first submission. Insert your Data Availability Statement in the box below.<br><br>Please see the data reporting section of our submission guidelines for instructions on what you need to include in your Data Availability Statement. Examples of acceptable Data Availability Statements are included here for reference:<br>1. The minimal data set [and accompanying code, where generated] is available at [repository name] via [DOI hyperlink]<br>2. No data was generated by this study. The following existing data sources were used: [dataset name] from [dataset location] available via [web address or DOI hyperlink].<br>3. For Study Protocols: No datasets were generated or analysed during the current study. All relevant data from this study will be made available upon study completion.<br><br>*PLOS allows rare exemptions to address legal and ethical concerns. If you have legal or ethical restrictions, please use the box below to detail these in full sentences for the Journal team to consider.*<br><br>This statement will be typeset if the manuscript is accepted for publication.	

Manuscript Click here to access/download;Manuscript;Manuscript.docx

"I want to take the power back": An interpretative phenomenological analysis of self-management in coexisting physical and mental long-term conditions

Catherine Lodwick [1*], Leire Ambrosio [1,2], Mari Carmen Portillo [1,2]

[1] School of Health Sciences, University of Southampton, Southampton, UK

[2] National Institute for Health and Care Research (NIHR) Applied Research Collaboration Wessex, Southampton, UK

* Correspondence concerning this article should be addressed to Catherine Lodwick.

Email: cel1g20@soton.ac.uk (CL)

Authors' Contributions

Catherine Lodwick: Conceptualization, Data curation, Formal analysis, Funding acquisition, Investigation, Methodology, Project administration, Writing – original draft, Writing – reviewing & editing

Leire Ambrosio: Funding acquisition, Supervision, Writing – review & editing

Mari Carmen Portillo: Conceptualization, Formal Analysis, Supervision, Writing – review & editing

Abstract

Introduction: Individuals living with both physical and mental health conditions face compounding barriers to self-management. These challenges arise from complex care needs, interactions between long-term conditions symptoms and inequalities resulting in a high treatment burden. While previous research on coexisting conditions has largely focused on those with severe mental illness, there remains an important gap in understanding the factors that help or hinder self-management among adults living with coexisting physical and common mental health conditions (anxiety/depression) in the community.

Objective: This study aims to explore the facilitators and barriers to self-management for adults living with a physical long-term condition and coexisting anxiety or depression in the community.

Methods: A qualitative study with in-depth semi-structured interviews was conducted between May and August 2025. Eight adults living with coexisting physical and mental health conditions in the UK participated. Interview data was recorded, transcribed and analysed using Interpretative Phenomenological Analysis.

Results: Three main themes were generated; (1) Moving beyond sink or swim: scaffolding support to self-manage (2) Self-managing: a choice or systemic exclusion (3) Unseen struggles: learning to live with my long-term conditions. Participants described the complex, often unsupported reality of self-managing coexisting long-term conditions, where many felt forced to integrate self-management into their lives due to systemic gaps and negative interactions with healthcare professional. Their experiences demonstrated an apparent hinderance to self-management, with age, condition visibility and identity struggles creating compounding barriers. Therefore, a

holistic scaffolded support approach promoting integrated care was suggested to meet both their health and social needs.

Conclusions: Our study provides insight into self-management facilitators and barriers among adults living with coexisting physical-mental health long-term conditions, highlighting substantial barriers to self-management and the need for person-centred, flexible, integrated support. The findings emphasise the need for new care models reflecting evolving presentations and domains of coexisting conditions.

Introduction

In recent decades, the burden of living with long-term conditions (LTCs) has increased markedly. In the United Kingdom one in four adults, equating to 15 million people, live with at least two LTCs which are lifelong and cannot be cured [1]. Alongside this, NHS England and Improvement [2] estimate 30% of those living with LTCs have a coexisting mental health condition. It is plausible this figure has continued to rise, particularly as the World Health Organization [3] reported a 25% global prevalence increase in anxiety and depression following the COVID-19 pandemic thus demonstrating their expanding contribution to the global disease burden, highlighting the significant public health impact of coexisting physical and mental LTCs.

The growth in prevalence of coexisting conditions has far reaching implications not only for individuals but also their families and the wider health and social care systems [4]. In the UK, The Kings Fund [5] estimated the collective cost of coexisting physical and mental LTCs to NHS England to be more than £11 billion annually, with the Mental Health Foundation [6] finding a further £88 billion attributed to loss of productivity. Whilst there is currently no global consensus on the economic impact of coexisting LTCs, the World Health Organization [7,8] recognises that they significantly drive early mortality, raise perceived and actual treatment burden, and reduce productivity. All these factors have their own profound fiscal implications which drive long-term financial strain on national and global public services. Yet beyond the financial costs, living with coexisting physical and mental LTCs can be challenging for the individual as it creates complex, multidimensional care needs due to inequalities and condition interactions. Ultimately the interplay from coexisting

conditions may cause poor care adherence, psychological distress and social isolation, which when combined lead to a significantly lower quality of life and higher healthcare utilisation compared to those living with a single LTC [9,10]. Notwithstanding, it is essential to highlight for this population not all care experiences are positive with many individuals' reporting stigma, service fragmentation, and diagnostic overshadowing whereby a service users' symptoms may be attributed to their mental health condition overlooking possible medical causes [11]. This disruption to care adversely impacts their wellbeing and trust in health and social care professionals [12].

In response to these challenges, there has been growing emphasis across clinical policies, practice and public health. Most recently observed in the NHS 10 Year plan, which is deeply focused on empowering individuals to be more active in managing their own health and wellbeing within community settings [1,13,14]. An important pillar of this plan focuses on facilitating the widespread adoption of digital tools, personalised care plans and self-management initiatives among service users and providers, which aligns directly with this study's aim. Self-management refers to a combination of strategies from medication management, clinical treatments and behavioural changes to promote good health outcomes and improve one's quality of life [15]. Although self-management is common amongst the general population, approaches used do not always account for the bidirectional relationship and specific needs of those living with coexisting physical and mental LTCs which produce a spectrum of challenges and contributes to widening health and social inequalities [16-18].

The World Health Organization define mental health as a state of mental wellbeing which enables individuals to cope with life stressors, learn and work well and

contribute to their community [19]. Common mental ill health symptoms including low mood, anxiety, fatigue, and difficulties with motivation and concentration may reduce an individual’s capacity to self-manage day-to-day aspects of LTCs, including medication adherence, symptom monitoring, lifestyle modification, and engagement with healthcare services [20,21]. To manage these challenges alongside physical LTC, individuals may establish routines, use self-regulation and coping techniques, and draw on both informal support from friends and family and formal support from health and social care professionals [22]. Formal support for anxiety and depression may involve psychological therapies and pharmacological treatments; however, variability in their accessibility, continuity, and perceived effectiveness may further affect sustained self-management behaviours [23]. As recognised in the NHS Fit for the Future [14] plan, these obstacles highlight the need for integrated and person-centred care models which address both physical and mental health needs simultaneously, support continuity across services and recognise the interaction between coexisting LTCs.

Despite an increasing evidence-based on self-management interventions and support programmes for LTCs, the focus primarily remains on single-disease models, physical health conditions with clear biomedical markers and severe mental illnesses [24-26]. While Balogun-Katung et al. [27] and Coventry et al. [28] have begun to broadly examine barriers and enablers to self-managing coexisting LTCs, their research remains centred on those living with severe mental illness such as schizophrenia and bipolar disorder. In contrast, recent qualitative studies by Beaudin et al. [29] and Kappelin et al. [30] focus on common mental health conditions although from the perspective of healthcare professionals. This research illustrate that self-management support continues to be delivered in silos, despite

acknowledging coexisting conditions, with practice continuing to show favour towards physical LTCs. Despite the recognition of integrated care in both international policies and guidelines, including The Big Mental Health Report [31] Major Conditions Strategy [1], UN Sustainable Development Goals [32], and WHO Policy on Mental Health [33]. Their objectives are explicit, stating the need to embed mental health support within physical health services and vice versa, with the aspiration to deliver holistic and coordinated care, yet this clearly remains a persistent challenge.

The current care model does not capture coexisting mental LTCs, considering them as secondary concerns rather than integral components of LTC self-management. Therefore, the way living with coexisting LTCs impacts an individual's systemic, relational and personal contexts are insufficiently understood [34-36]. As a result, there continues to be a limited awareness of how individuals self-manage the multidimensional and intersecting demands of coexisting physical LTCs and anxiety/depression. This qualitative study offers originality by aiming to explore facilitators and barriers to self-management among adults living with both a physical LTC and anxiety/depression in the community. By addressing this critical knowledge gap, the present study has the potential to inform the design of a person-centred and contextually responsive self-management interventions, thereby, contributing to the reduction of health and social inequalities and enhancing the health and wellbeing outcomes for this underserved population.

Materials and Methods

Study Design

This was a qualitative study with interpretative phenomenological analysis (IPA) focused on the self-management experiences of adults living with coexisting physical LTCs and anxiety/depression. The IPA paradigm was deemed appropriate for this subject opposed to other broader qualitative inquiry approaches as its grounding in hermeneutic phenomenology aims to gain an in-depth understanding of an individual's lived experience, alongside its idiographic nature, enabling rigorous interpretation on the meaning of an individual's experience [37,38]. The interpretative stance of IPA facilitated a double hermeneutic process, an approach more congruent with the study's phenomenological aim opposed to the systematic abstraction of grounded theory or observational immersion of ethnography, which was considered especially relevant for this study [39,40]. By promoting the researchers to deeply and systematically engage with participant's realities and sense-making, this study's design choice strengthens its trustworthiness [41,42]. To enhance the quality and rigor of results, the COREQ checklist created by Tong, Sainsbury, and Craig [43] was used and can be found in S1 Checklist.

Sampling, study participants, and recruitment

Aligning with IPA practices, purposeful sampling was applied as it identifies individuals or groups with knowledge or experiences relevant to the phenomenon of interest; thus, this study focused on adults self-managing coexisting physical LTCs alongside anxiety and/or depression [44,45]. Participants were considered eligible to participate if they had a history of coexisting physical LTCs and

anxiety and/or depression, lived in Southeast England, were aged 18 and over. Participants' LTCs were self-reported to enhance accessibility and reduce participation barriers, including limited feasibility and time to obtain or verify clinical information with healthcare providers. Those living in inpatient or residential settings were excluded as their self-management may be influenced or carried out by others. Sampling was congruent with the data analysis process and continued until no new ideas emerged, inferring information power had been achieved [46,47].

Recruitment for the study commenced on the 3rd of May 2025 and concluded on the 31st of July 2025 and occurred using three approaches. Firstly, recruitment posters were displayed in the Hampshire area. Secondly, social media channels (Instagram) advertised the research study and finally, two charitable organisations (Age UK, Communicare) were contacted, obtaining managerial approval, to publicise recruitment materials to members of their network. Potentially interested participants initially emailed the primary author using contact information provided on the recruitment poster. Upon expressing interest, participants completed a screen questionnaire to ensure eligibility. Where all criteria were met, participant information sheets and consent forms were sent to participants via email. Participants returned informed written consent forms, if they wished to proceed, via SafeSend software to ensure secure transfer of personal information. This was followed by the participant and the author arranging a mutually convenient interview date and time.

Data Collection

Participants were individually interviewed using a semi-structured format with open-ended questions, designed to encourage participants to provide a rich account of their lived experiences with minimal direction from the researcher. Following

consultations with the PPIEP group and due to pragmatic considerations, interviews were conducted on Microsoft Teams which were audio and video recorded. The interview topic guide, found in S1 Table, was generated based on the existing literature and feedback from MCP and the study's PPIEP group. All interviews were conducted between May and August 2025 and lasted between 40-70 minutes. Recordings were securely stored on a password protected computer in accordance with the Data Protection Act 2018 [48]. The researchers had no therapeutic relationship with study participants.

Participation and Public Involvement, Engagement and Participation (PPIEP)

Two PPIEP members were consulted throughout all stages of the research process to ensure the study remained grounded in service user priorities with lived experience of coexisting LTCs, enhancing rigor and credibility. One member was a White mental health nurse living with anxiety, depression and several physical LTCs and the second member was a service user from the BAME (Black, Asian, and Minority Ethnic) community living with urinary and reproductive LTCs alongside anxiety and depression, which provided a range of knowledge and experiences. Meetings were held with the lead researcher (CL) and PPIEP members both in-person and online depending on the individual's availability. Both PPIEP members contributed to the study from designing the scope and direction, topic guide development, theme naming and refinement. Key contributions from discussions between PPIEP members and CL included providing guidance on interview settings as discussions indicated that while in-person interviews may have further supported rapport-building, they were less feasible due to logistical constraints and the

fluctuating nature of living with LTCs. As such, an online approach was chosen to accommodate participants' varying needs and circumstances whilst allowing a more personable and interactive experience than telephone calls, which was crucial given the sensitive nature of discussing self-management experiences. Their extended contributions and engagement have been evaluated using the GRIPP2-SF as recommended by Staniszewska et al. [49] to ensure reporting transparency, see S2 Checklist.

Data Analysis

Each interview was transcribed verbatim with all personal identifying information removed and pseudonymised, with pseudonyms chosen by participants when arranging the interviews [50]. Data analysis began as soon as each interview was transcribed, employing the six step IPA process by Smith et al. [38]. No digital software was used to analyse the data as Smith et al. [38] encourage use of the paper-pencil method to promote closeness and deep engagement when analysing and critically interpreting the data.

Initially, the transcripts were printed and numbered with continuous lines. Transcripts were examined one-by-one to gain a clear picture of the individual's lived self-management experience. The inductive analysis process began by creating exploratory notes focusing on three distinct categories: descriptive, linguistic, and conceptual, marked by corresponding coloured pens on the right-hand side of the margins. Descriptive comments focused on the participants' accounts of key events, experiences, and emotions. Linguistic notes attended to the participants' use of language, such as tone, repetition, pauses, metaphors, emphasis, or pronoun use. Conceptual codes involved a more interpretative level of engagement, exploring

underlying assumptions, possible meanings and connections between participant's experiences and broader psychological or experiential concepts. From the exploratory notes, experiential statements were constructed, written on the left-hand side of the margins, summarising the transcription as well as exploratory notes. This process involved synthesising descriptive, linguistic, and conceptual observations into concise interpretative statements which captured the lived meaning of participant's experiences. The researcher then cut the transcript into these experiential statements, accompanied by the corresponding transcript lines and exploratory notes. These experiential statements were then laid out to visually facilitate a systematic exploration of connections, similarities, differences, and patterns across the data. Through this iterative process, related statements were clustered together to generate Personal Experiential Themes (PETs), capturing how individual participants understood and made meaning of their lived experiences. See S2 Table for further illustration of the process of developing exploratory notes, into experiential statements and subsequent themes.

This process was applied to all eight transcripts after which a summary document was produced for each participant's PETs. Following refinement of all PETs, theme summaries were reorganised to enable comparison across participants and exploration of patterns and relationships between accounts. This iterative process informed the development of Group Experiential Themes (GETs), representing shared experiential meanings across participants, and were developed when a theme was present in at least half of the accounts. The findings of this study underwent a rigorous analysis and interpretation process, thus resulting in the creation of analytical themes which represent a synthesis of participant accounts, interpretative engagement, and theoretically informed

understandings [51]. Consistent with the theoretical and interpretative underpinnings of IPA, the GETs were examined at increasing levels of abstraction through a theory-informed interpretative lens. The aim of this process was not to generate formal theory, but to deepen understanding of participants lived experience and position the findings in relation to existing scholarship [55,53]. To ensure analytical robustness, two of the authors (CL and MCP) corresponded monthly to discuss and revise the GETs and subthemes. Member checking was not employed as a validation strategy reflecting recent discourse questioning its suitability within IPA [54]. Instead, credibility was enhanced using peer debriefing, audit trails, and the inclusion of thick descriptions [41,55].

Ethical Considerations

Ethical approval was obtained from the University of Southampton Ethics Committee and the Research Integrity and Governance offices under the reference ERGO 102445, see S1 and S2 Fig. As this research study was unfunded, no financial or material compensation was provided to participants. All the research conforms to the ethical principles for medical research on human beings set out in the Declaration of Helsinki [56].

Results

Study participants

A total of ten potential participants contacted the researcher (CL). However, one did not complete the follow-up, and another withdrew at interview stage. Whilst the

concept of information power influenced the overall sampling approach, only eight participants were interviewed and included in the final analysis of this study [46]. Despite this, the dataset was deemed sufficient to support meaningful analysis commensurate with IPA philosophy which prioritises depth of insights opposed to frequency to preserve the nuanced interpretation which IPA demands. The characteristics of included participants (Table 1) consisted of seven women and one man, aged between 18 and 34, with physical LTCs encompassing a scope of body systems from hepatic, gastrointestinal, reproductive, musculoskeletal and the nervous system. This small sample is common within IPA studies aligning with recommended ranges of four to ten participants, allowing for more in-depth interpretation opposed to superficial understanding which may accompany a more diverse sample [57].

Table 1: Characteristics of interview participants

Participant	Gender	Age	Physical LTCs	Mental LTCs
Rosemary	Female	25-34	Non-Alcoholic Fatty Liver Disease (NAFLD), Irritable Bowel Syndrome (IBS)	Anxiety and Depression
Grace	Female	18-24	Scoliosis, Irritable Bowel Syndrome (IBS)	Anxiety and Depression
Poppy	Female	18-24	Hypermobile Ehlers-Danlos Syndrome (hEDS), Chronic fatigue syndrome (CFS), Chronic migraines	Anxiety and Depression
Ruby	Female	18-24	Chronic constipation, Irritable Bowel Syndrome (IBS), Hypermobility	Anxiety and Depression
Oskar	Male	18-24	Gout	Anxiety
Ashley	Female	18-24	Hypermobile Ehlers-Danlos Syndrome (hEDS), Postural	Anxiety and Depression

			Orthostatic Tachycardia Syndrome (PoTS)	
Laticia	Female	18-24	Irritable Bowel Syndrome (IBS)	Anxiety and Depression
Simone	Female	18-24	Endometriosis, Adenomyosis	Anxiety

Names included are pseudonyms to maintain anonymity.

Themes and subthemes

The analysis process revealed high levels of convergence across the eight interviews which produced three Group Experiential Themes centered on the facilitators and barriers encountered when self-managing coexisting physical and mental LTCs. The themes alongside their associated sub-themes are detailed in Table 2. Furthermore, each participant's contribution to the development of GETs is illustrated in S3 Table.

Table 2: Description of themes and subthemes

Theme	Description of themes	Subthemes
Moving beyond sink or swim: scaffolding support to self-manage	Articulated the experiences of self-managing coexisting LTCs in the community, through balancing condition demands and calling for more holistic strategies.	"An internal battle": living with coexisting conditions<br>Seeing me, not just my long-term conditions
Self-managing: a choice or systemic exclusion	Focused on a critical exploration of structural barriers in the healthcare system for adults living with coexisting physical and mental LTCs, and how people responded by creating their own individualised self-management meaning.	"You just feel lost": Navigating the healthcare system<br>"Taking control" through self-management
Unseen struggles: learning to live	Centred on how participants felt their lived experiences were undermined, either due to age or	My medical identity vs my social identity

with my long-term conditions	the invisibility of LTCs leading to internal struggles regarding their identities.	Self-managing now for my future

Theme 1. Moving beyond sink or swim: scaffolding support to self-manage

“An internal battle”: living with coexisting conditions

All participants described a deeply “interlinking” relationship between their physical and mental health LTCs, whereby one condition intensified the other’s symptoms often negatively. This bidirectional relationship made addressing coexisting LTCs simultaneously very challenging, frequently resulting in the prioritisation of one condition with remaining LTCs forced to take a “backseat”. For many, there was a continual shifting of priorities depending on the LTC causing the most prevalent concerns, repeatedly reported as pain or the external effects of mental health on friends and family. This fluidity contributed to a cycle of fatigue, emotional exhaustion, and frustration.

> *“It’s like pushing a boulder up a hill and if I take my hand off that boulder to focus on my mental health and that boulder starts rolling, it’s just like, uh my God. Now we have to start all of this again” (Rosemary)*

Seven participants described managing their mental and physical LTCs concurrently as an ongoing challenge, referred to as an “internal battle” or “double edged blade”. All participants went on to note unlike their physical LTCs which they felt had clear, more tangible self-management strategies, mental health concerns were harder to address and slower to resolve.

> *"I think, I think I personally prioritise my mental health over my physical health just because as long as I'm not like acutely unwell physically I can get back on top of my physical health but sometimes I know that if my mental health suffers it takes longer to get out of that kind of rut than it would if I was physically ill. Um so I do prioritise my mental health just because I know the consequences can be a bit more severe in terms of it I kind of slip on that end of things." (Ashley)*

This lack of immediate identification and relief often led to reduced motivation to engage in self-management activities, feeding into a "downward spiral". Overall, participants conveyed that the interplay between LTCs and constant renegotiation of self-management strategies had a significant emotional and physical impact on their daily lives.

> *"You kind of just don't want to bother with self-managing after that because you're like what's the point if it's, if it's not worked, what's the point? And you sort of, sort of I don't know how to describe it, it's more, it's like a pit you sort of just get stuck in this pit of what's the point, because nothing works and I just have to deal with it but I don't want to deal with it and so you're just stuck in this like little downward spiral almost." (Laticia)*

Seeing me, not just my long-term conditions

Despite the complexity of managing coexisting conditions, participants reported a persistent expectation to adhere to standardised models of care. Grace and Simone described these experiences as dehumanising making them feel like "guinea pigs" in a system overly focused on medications. Further supported by a strong rejection of a

"one-size-fits-all" approach with participants instead calling for tailored strategies that acknowledge both the individual and specific interplay of their LTCs.

> *"Instead, quite often I'm faced with you need to do this and you need to do that and I'm like, ok, but we're not really thinking about what does and doesn't work. We're kind of trying to put that one-size-fits-all thing." (Rosemary)*

> *"Yeah, the WRAP [Wellness Recovery Action Plan] planning for mental health, I think that was really good in making me identify what kind of like, asking those questions and putting it back to me to kind of tell. Making me kind of work out what helped, like facilitating me to work out what helped me. They, I think it's a lot of like just communicating to me that they were hopeful for me to go and often live this like good life whilst self-managing. I think it was just their overall approach of being very like hopeful and realistic and listening to me about what worked and what didn't work. And like when things weren't going so well just involving me and making the decisions to do the things to tailor my treatment or like make a plan like very much involving me in the planning. Whereas like with the GP, they just made the plan and gave it to me." (Ruby)*

Although recognised by all participants, this need was especially pronounced among Poppy, Ruby and Oskar who wanted healthcare professionals to acknowledge self-management "from the get go" of their chronic illness "journeys", as they found receiving diagnoses and treatment planning to be overwhelming moments.

> *"I think it wasn't really, when I was getting my diagnosis it wasn't really emphasised that how controllable this is. I think it's more something that I've learned with time. Um, whereas like I think some reassurance from the start of*

hey look, this is like this is a very treatable thing, yeah, like, this is a very manageable problem like it would help with not panicking at the, at the start" (Oskar)

Six participants emphasised the importance of early interventions and education on coexisting LTCs, including timely conversations post-diagnosis which explore personalised self-management strategies, reinforcing motivation and helping to establish individualised goals.

"I think as a healthcare professional acknowledging that is so important, like talking about exercise, talking about hobbies, you know, Maslow's hierarchy of needs what's at the bottom part like what's your purpose kind of thing I think is really important' (Simone)

A consensus among participants was that feeling heard and collaboration with healthcare providers when planning care are critical factors to long-term engagement. However, to achieve this goal there is a need to move away from the current perceived sink or swim model which places sole responsibility on the individual and their personal resources, to a collaborative approach where support is scaffolded over time.

"It has just been a lot of throwing them in the deep end and hope that they can swim. It definitely has been an approach of that and so a lot of the stuff that you figure out, you either figure out on by yourself or using your own resources such as paying for counselling." (Laticia)

Although when planning care, Poppy stressed the value of practical over theoretical tools. This opinion was supported by Ruby, who together highlighted the importance of usable, realistic approaches tailored to the challenges of managing coexisting

LTCs, although they hadn't personally found one yet. However, Simone spoke of her "toolkit", whereby self-management strategies are colour-coded green, orange or red to represent corresponding symptom severity. This approach was designed in collaboration with healthcare providers creating a tangible, effective self-management plan which aided her to "kill two birds with one stone" referring to her coexisting LTCs.

> *"We've made a plan. In fact, we've made a plan, here's our number at the bottom we might not get back to you for eight weeks but you know we've got it. I think that gives someone, that puts them in control of their outcomes and their own health and do you know what I mean, in like a more palatable way" (Simone)*

Theme 2. Self-managing: a choice or systemic exclusion

"You just feel lost": Navigating the healthcare system

Whilst most participants wanted the healthcare system to move towards a "holistic route", their current reality was often opposite. Many participants described damaged relationships with healthcare professionals characterised by feeling dismissed, ignored or disbelieved, which was regularly linked to diagnostic overshadowing.

> *"I just think that when you go with a problem they should consider your whole, your whole story instead of, 'cause I've not only had like, it's not just. If I go there and say I'm, you know, I feel sick or whatever, um I think it's quite easy for them to say, oh, it's probably you're probably anxious or whatever when really, well you should actually look into that, because I've also had surgery.*

So, you could be missing something else type thing, but, yeah, I don't know, I just think they should consider the story as a whole and not. They should take more time before they come to a conclusion, I think" (Grace)

Enduring these "constant experiences" contributed towards a resentment of healthcare providers among seven participants. These participants withdrew from formal care and turned towards self-management, not as a preference for LTC management but a necessity born out of advocacy fatigue.

"So, it was kind of a it was a bit of a fight with my GP trying to just get like the testing done and, and push for the diagnosis just because then it did entitle me to like it just allowed me to have more understanding about myself." (Ashley)

Instead of traditional services, informal sources of support such as online communities, social media, and support groups were more popular. These spaces offered validation and understanding, regularly missing in clinical encounters, although Poppy noted the absence of professional healthcare involvement could foster uncertainty about the reliability of shared advice creating self-doubt in one's self-management abilities.

"Um I worry that like I'm possibly not doing the right things or there are things I'm missing, because I'm not having any input from people that really know what they're talking about" (Poppy)

Structural barriers within the healthcare system were considered by all participants. They highlighted poor communication, fragmented care pathways, complex referral processes, and geographical constraints as persistent obstacles to self-managing their coexisting LTCs. There was a clear need to streamline services and integrate

care to create healthcare systems which are inclusive and responsive opposed to exclusionary and delayed.

"Taking control" through self-management

Despite their common negative care experiences, all participants recounted a gradual shift from service reliance to personal agency in self-managing their LTCs. All participants recounted how the meaning of self-management had evolved into some form of "taking control", whereby traditional clinical outcomes were replaced with personal definitions such as achieving a sense of normality, independence, happiness, productivity or simply basic functioning. As a method to achieve these goals, knowledge emerged as a key tool, with participants using a variety of strategies to build their understanding including peer learning, trial and error, formal education, and personal research.

> *"I've had a lot of time for trial and error. I've had a lot of time to do different things, find what works for me and used like different tools to kind of find what works for me." (Ruby)*

> *"I think my mum looking into how to properly self-manage and all of that and she like gave me these like cliff notes basically that I could like look into myself because at the time where this information was like when my mum was looking into it, I was in the middle of a gout attack. I wasn't looking up anything for myself. Yeah, that was actually the most helpful thing anyone's ever done." (Oskar)*

However, when gathering this information Rosemary and Ashley encountered difficulties accessing relevant condition specific information, especially when unwell

which felt like a “backwards” process. Supported by Oskar and Laticia who recounted knowledge finding as an “energy consuming task” which was at odds with trying to meet their basic daily needs. Therefore, recommendations for a reliable “bank” of information from the healthcare systems were idealised to mitigate this barrier.

> *“I understand that GPs are generalists, but it would be nice if they had, you know, sort of a bank. You know how, like GPs, they’ve always got leaflets or information sheets, it would be nice if those kind of existed about my condition that they could pass on instead of me being the one going out and doing all of this research” (Rosemary)*

Where this information was already available, through charity websites, Poppy expressed a strong desire for information to be presented from a holistic self-management lens rather than a purely biomedical perspective.

> *“There’s some really good like guidance on Ehlers-Danlos, they have on their own website which has some really helpful stuff. But a lot of it, like when I look into it, it’s either really fundamental stuff or it’s not written in a perspective of self-management at all. It’s written in like these are the biological components of hypermobility. And I’m like, ugh, I don’t care” (Poppy)*

Theme 3. Unseen struggles: learning to live with my long-term conditions

My medical identity vs my social identity

A key barrier to self-management expressed by all participants was how ageism and stigma significantly shaped their experiences of care, with their youth causing their concerns to be invalidated or second-guessed.

> *"Ugh to be honest I just showed up one day, the woman looked at my foot and she said it looks like gout, but you're young so do you have a family history?" (Oskar)*

Many felt age created a hierarchy of legitimacy where youth conflicted with perceptions of illness, leaving them to feel invisible or misunderstood. This tension was especially pronounced for Rosemary and Poppy, who revealed this feeling was further complicated by living with LTCs which lacked objective physical signs. The absence of visible symptoms appeared to undermine their credibility in social and clinical settings, causing them to "doubt" their level of illness and intensified feelings of exclusion.

> *"Um, it just feels like everything that I've come up against is kind of you have to do it and you have to be the only person in it when that's just not the way that I'm able to operate you know, like it's if a person, I don't know, needed a crutch or a walking stick, but for some reason the GP wants them to walk through the surgery without a walking stick that just feels a bit irritating." (Rosemary)*

"I just have this thought of, like, we are all just tired and like just go to uni and get a grip, and it's not that big of a deal and actually it's not normal and it's, it's not something everyone deals with um and trying to remember that because I can be really hard on myself and be like, why can't I just push through? But actually, because it's a medical condition, you wouldn't tell someone with like asthma to push through an asthma attack like." (Poppy)

While there was a desire for greater recognition and visibility of younger people with less-represented LTCs, Simone feared being boxed in or stereotyped as a result.

"I understand that there needs to be visibility with chronic pain conditions and the more we talk about it, but I did wonder about the stigma and the label of labelling myself as someone like if I presume this identity of someone with chronic pain what have I, have I actually put myself into a box where I can't, do you know what I mean? It becomes part of your identity... I tried it and realised actually I've just minimised myself more as a person because now I'm just some you know [18-24]-year-old with chronic pain and not someone who likes going out." (Simone)

These experiences contributed to a blurring of identities: where medical needs became entangled with personal identity, overwhelming their sense of self. However, obtaining formal diagnoses was viewed as both gateways to care and burdensome, concurrently validating experiences whilst constraining identity. Balancing medical and social identities was particularly difficult for those in their early twenties who spoke about feeling torn when managing their health and maintaining friendships, relationships and a sense of normality. This created an internal conflict marked by sacrifice and loss as participants struggled to reconcile youth with chronic illness.

> *"I didn't want to be that like mentally ill friend that was only talking about mental her mental illness. That friend that was always like kind of dragging people down or like, oh, my mental health is rubbish today." (Ashley)*

> *"I love festivals and I love music and I think when my symptoms got out of control and I was like I actually don't, I don't know if I can do this and this is really upsetting." (Simone)*

Self-managing now for my future

Following the dilemma of being younger and living with LTCs, participants highlighted significant gaps in self-management resources tailored towards younger people. They found information about self-management to have a primary condition focus or be "catered" towards older generations, failing to reflect the realities of youth. There was clear frustration at the assumption of younger people being uninterested in self-managing, where in fact participants expressed strong motivation to learn and adopt strategies to maintain their health. Poppy, Oskar and Simone wanted "sustainable" solutions that allowed them to participate in social activities like clubbing, drinking or festivals without forfeiting their mental or physical wellbeing.

> *"I also think like a lot of self-management, I see definitely tends to be catered towards like an older generation. Like, it's like, oh you want to move more? Go out in the garden or play bowls...but the, the like sustainable lifestyle changes don't often seem to accommodate for being younger and like your social groups looking different and being at university, or because I think they just assume that people aren't trying to self-manage in those situations. And*

it, it makes it really difficult because it, it does like when I look into things, it's like it just doesn't feel like it's catered to me and my life at all." (Poppy)

A key theme raised was the need for healthcare providers to recognise varying life stages alter how LTCs impact an individual's life and therefore their self-management approach. Both Grace and Ruby recalled losing the ability to self-manage in childhood due to parental or institutional involvement. These events shaped their views of self-management in adulthood, with Grace wanting to see self-management as a choice not a discipline, whilst Ruby viewed it as a privilege after it was "taken out of my hands".

"So, it's almost like I work harder to self-manage because I don't want to experience that again but then also I feel like, it's like for me part of it is having the power of being able to self-manage myself like it's such because its something that I haven't been able to do before, um or like it's more been taken away from me. Because I wasn't in a place to do that, it's, I almost feel like I've got a bit of like, it's a bit of a privilege to be able to do that myself." (Ruby)

A further barrier was the emotional and practical toll of self-managing as an emerging adult, having to account for future deterioration and LTC progression. Ruby and Poppy noted this outlook as "quite a hard thing to accept" but were resolved to incorporate self-management as a proactive measure to age well. Whereas Simone determined it was best for her to live in the present "I kind of just do what I want and then reap the consequences".

"I try my best to make choices that will help me age well, but there's definitely some fear of like, what's my future going to look life if things, things could get

progressively worse. So, I have a component of me that's that it's quite scary and then there's just also bits of me that just get annoyed. I'm like, what, for God's sake [laughs], I don't need to like, I don't need to be thinking about this at [18-24] but like also I do. But you know, one of those." (Poppy)

Other key transitions of aging such as moving between school, university and work arose as vulnerable periods marked by gaps in support, embarrassment around disclosing health needs and uncertainty requesting reasonable adjustments. Ashley and Laticia discussed the lack of employer knowledge of coinciding conditions, and these were evident areas of improvement needed to foster self-management.

"The services that you need to go to in your like workplace aren't as obvious as well. Like I didn't know about the basically what is like the DSA [Disabled Students' Allowance] equivalent of working like and I've already forgotten it's name, but like access to work, I think it's yeah access to work. I didn't know anything about that. No one told me. It's that, that into that's this not the seamless, what's the word, that sort of movement on from university on from education into like working life. Like no one prepares you for that so you don't know what you have available and even when you go looking, it's not that obvious. Like they leave it quite up to you which don't get me wrong is, is good in some parts but at the same time it's I also don't know because it's my first time in this situation and I don't know what I can ask for." (Laticia)

Despite these barriers, participants offered solution-focused suggestions such as digital profile packs detailing their LTCs and self-management needs to be sent to educational or occupational supervisors, streamlining advocacy processes and clearer support pathways. Their message was clear: young people are willing and

ready to self-manage but they need systems whether that be healthcare, higher education or occupational that are designed to support their self-management goals, not assumptions overlooking them.

Discussion

This qualitative study has explored the facilitators and barriers to self-management for adults living with coexisting physical and mental LTCs. Our findings add a new perspective to the literature by evidencing how developmental and life-stage factors may shape experiences of self-management and contribute to health and social inequalities. Previous studies exploring inequalities in multiple LTCs research have predominantly centred on socioeconomic status [58,59], education and health literacy [60-62] as primary determinants of disparity. While these factors remain important, our findings suggest that age and life-experiences may also heavily influence how individuals navigate the complexities of managing coexisting conditions. Although our sample was considered small and heavily weighted towards younger adults and women, which may have influenced the analysis and limits the transferability of the results, our findings suggest that young age represents an overlooked axis of inequality with emerging adults experiencing distinct, under-recognised challenges in self-management compared to other age groups. These findings contest dominant narratives found within current self-management frameworks, underscoring the need to reconsider how age-related developmental factors intersect with LTC management [63-65].

The findings from our study offer a conceptual leap by providing detailed accounts of participants shared, complex, and varied experiences of self-managing coexisting

physical and mental LTCs. Participants recalled constant negative interactions with healthcare professionals and services which they attributed to either their age or the complexity of living with coexisting LTCs. Our data showed these experiences detrimentally impacted their trust and view of traditional healthcare services thus prompting a growing reliance on peer networks and social media platforms as alternative sources of self-management support, signalling a notable shift in both the locus and modality of self-management behaviours. Furthermore, the analysis indicated pronounced healthcare disparities owing to the visibility or invisibility of their coexisting conditions, which shaped how their care needs were recognised and responded to by others. Moreover, participants highlighted a lack of scaffolded self-management support specifically tailored to coexisting conditions, reflecting broader gaps within both healthcare provision and support resources regarding integrated self-management for concurrent mental and physical LTCs. Collectively, these findings demonstrate the critical reform required in both professional and societal understandings of living with and self-managing coexisting conditions to better support this population to live well. These insights must be integrated into policy and practice to help prevent the further widening of health and social inequalities.

Compared with previous literature, our research both aligns with and diverges from dominant perspectives of self-management [66-68]. Whilst existing literature has addressed aspects of systematic facilitators and barriers to self-management for adults living with coexisting LTCS [27,69,70], concurrent with our findings, few papers have explicitly isolated younger age as a central concept to health and social inequalities within the self-management of coexisting LTCs. Despite being routinely collected, age is seldom critically examined beyond the context of aging in self-

management. As demonstrated in our results, young adults living with coexisting LTCs often experience stigma both from healthcare professionals and peers when discussing their LTCs, leading to experiences of self-doubt, resentment and feeling both medically and socially excluded. The youth-age dichotomy often constructs these life stages as oppositional, reinforcing assumptions of youth equating health, adaptability and resilience. However, this overlooks the profound self-management challenges faced by younger adults, which whilst different in nature are equally as significant as those faced by older populations [71,72]. Although the World Health Organization [8,73] recognises age as a social determinant of health, national and international guidelines [1,14,74] remain largely age-neutral, failing to account for the developmental and lifestyle factors which shape self-management experiences across the life course. While older and working-age adults are likely to experience distinct barriers to self-management [75], these challenges are more commonly represented within existing research and policy discourse, whereas the experiences of younger adults remain comparatively underexplored. Recent evidence, including the NHS Fit for the Future Plan [14] demonstrates that LTC prevalence is no longer driven solely by an aging population, with increasing numbers of working-age and young adults affected. Nonetheless, policy responses continue to prioritise older adults, framed through deficit-based narratives of cognitive decline or low health and digital literacy [76,77]. Whilst emerging initiatives, such as Young Future Hubs, remain limited in scope supporting only those under twenty-five and focusing primarily on mental health and social needs [78]. This leaves a significant care gap for emerging adults living with coexisting LTCs who require tailored and integrated support for their physical, mental, and social care needs. Our study challenges the prevailing assumption that younger age implies a lesser need or greater capacity for

self-management. Such assumptions marginalise emerging adults living with coexisting LTCs whose health and social care needs may fall outside of normative expectations across health, academic and occupational settings. These findings indicate current care pathways must be adapted to better support young adults with coexisting LTCs through integrated, developmentally appropriate self-management strategies.

Additionally, our findings further showed participants who lived with non-visible LTCs such as hypermobility spectrum disorder, irritable bowel syndrome, or mental health conditions faced significant barriers to professional recognition and self-management support. Although non-visible chronic illnesses have gained increasing public recognition in recent years, for example through initiatives such as the sunflower lanyard [79], individuals with non-visible LTCs continue to experience stigma and challenges to illness legitimacy in both medical and social settings [80]. Participants attributed these difficulties to care professionals' prioritisation of visible or objective indicators such as appearance, use of mobility aids, and abnormal diagnostic markers over lived experience and reported impacts on quality of life. This lack of recognition contributed to feelings of exclusion and stigma [12,81] reinforcing barriers to accessing care and wider support, including reasonable adjustments in education and employment [82,83]. Requiring individuals to prove the credibility of their ill health exemplifies epistemic injustice, distorts clinical encounters and undermines the therapeutic relationships essential to effective practice. These obstacles were compounded by self-management interventions narrowly tailored towards physical LTCs with clear biomedical markers and prescribed behaviours such as exercise, diet, and medication management [84,85]. Consequently, this focus marginalises individuals with invisible, fluctuating or complex LTCs, particularly

those with coexisting mental health conditions. Moreover, the limited or superficial integration of mental health support was misaligned with participants lived experiences, reducing engagement and intervention effectiveness. Participants reported current interventions failed to accommodate their coexisting LTCs often forcing them to prioritise one over another, despite MLTCs increasingly reflected in routine clinical practice. Without embedding mental health as a core component of LTC management, rather than an adjunct, adults with coexisting LTCs will continue to experience unmet needs, poorer health outcomes, and inequitable access to support [86-88]. To prevent further widening of health inequalities, self-management interventions must recognise invisible LTCs, incorporate subjective symptoms, and embed mental health as a foundational element.

Our findings illustrated that participants perceived a lack of tailored self-management support. Existing strategies were experienced as biomedical in focus; catered towards older adults; and insufficiently inclusive of different lifestyles. This was evidenced within the literature by a paucity of self-management frameworks targeted towards both emerging adults and those with coexisting physical-mental LTCs. Dominant care models underpinning self-management interventions such as the Chronic Care Model [89] and Bandura's self-efficacy theory [90], have limited applicability for these groups given their focus on single physical conditions and their presupposition for a level of autonomy, cognitive maturity, and life stability [91]. As such, participants described uncertainty about managing their LTCs cohesively without compromising social participation and relationships, which they identified as essential to maintaining mental wellbeing. In contrast, Paterson's Shifting Perspective Model of Chronic Illness [92], which conceptualises chronic illness as a dynamic and evolving experience across the life course, offers a more appropriate

framework for coexisting LTC self-management. The recognition that individuals move between periods of illness and wellness resonated strongly with participant narratives. Although our findings extend this model by suggesting that illness and wellbeing may coexist simultaneously rather than as alternating states. This reframing underlines the need for sustainable self-management resources, which enable individuals to prioritise wellbeing and social participation even during periods of illness. Notwithstanding, to avoid exacerbating health and social inequalities, the design of such resources must account for socioeconomic and cultural contexts as these factors have a moderating effect on self-management support [93,94]. Overall, our findings illustrate a conceptual leap for new self-management frameworks which consider managing coexisting LTC as a developmental process evolving alongside identity formation and illness trajectories.

Our findings provide an original contribution to this field of research by emphasising the complex interplay between age, identity and system navigation, offering a new conceptual lens to understand the facilitators and barriers to self-management of coexisting physical and mental LTCs. Rather than acting as a standalone determinant, age appears to interact with broader structural and individual factors, including identity formation and service organisation, shaping how individuals experience and engage with self-management practices. The accounts of emerging adults suggest that developmental context may be relevant to understand these processes across adulthood. Overall, the results emphasise the importance of developing age-sensitive self-management strategies, improving the visibility of coexisting LTCs within care provision, and ensuring mental health is recognised as a central and integral component of self-management alongside physical health needs.

Implications for practice, policy and research

Clinical practice and policy

Our study stresses the need to create age-sensitive self-management frameworks reflecting the distinctive biopsychosocial domains within young adulthood. As traditionally self-management models of care have focused on compliance and structured interventions tailored towards older generations which may be less effective for individuals still forming their identity and autonomy. Therefore, our findings offer a rationale for policymakers and practice providers to co-create a self-management framework which incorporates age-related barriers to engagement and explicitly embeds mental health support as a core component. Incorporating the priories and lived experiences of younger adults into clinical guidelines may enhance sustained engagement in both short and long-term self-management behaviours.

Future research

Future research is warranted to examine how young adulthood and intersecting sociological factors such as social media use, peer support, and unstructured lifestyles shape self-management intervention uptake. Moreover, quantitative expansion of this work could employ longitudinal surveys to systematically examine how key facilitators (social support, technology, integrated care) and barriers (lack of tailored guidance, care discontinuity, social stigma) influence the development and evolution of self-management behaviours across the life course in adults living with coexisting physical-mental LTCs. A crucial element of this approach would be ensuring maximum variation in sampling to achieve representation across key demographic variables such as age, gender, socioeconomic status and types of LTC combination alongside differing levels of digital engagement and healthcare access.

This would enable more robust comparative analyses across groups and strengthen the validity and transferability of findings. Based upon the results, there is potential to codesign targeted self-management interventions that integrate both medical and social needs, ensuring relevance and effectiveness for this underserved group.

Strengths and Limitations

A key strength of this study lies in its use of Interpretative Phenomenological Analysis which enables in-depth exploration of participants lived experiences. Although the small sample size, consistent with IPA's idiographic focus, may have limited the breadth of experiences captured; the recurrence of similar themes suggests a degree of convergence and robustness. Peer debriefing between two authors further strengthened the analytical process and supported the credibility of the findings. However, there were several limitations within this study to note. Firstly, the recruitment strategy relied heavily on digital access and skills, which may have excluded underserved or older populations. Additionally, the researchers did not formally track which recruitment methods yielded participants, limiting the ability to evaluate the effectiveness of recruitment streams and inform future research. Secondly, the sample size was relatively small and skewed towards female participants and younger adults, potentially reflecting self-selection bias and limiting transferability of the results. Finally, the absence of socioeconomic, educational, and employment data represents a key limitation, as collecting these demographic factors could have enabled a more nuanced interpretation of the findings.

Conclusion

This qualitative study examined the facilitators and barriers to self-management for adults living with physical LTCs and coexisting anxiety/depression. Overall, the study contributes to a more nuanced understanding of the lived experience of managing coexisting physical and mental LTCs, emphasising the need for person-cantered, flexible, and age-appropriate responses to self-management support. While our study centred on the voices of emerging adults, the study provides a foundation for further research comparing self-management experiences across different age groups of adults living with coexisting conditions. Such work would support the development of life-course informed approaches to self-management that better reflect the developmental context and evolving needs of individuals across adulthood, which may inform the design of more tailored and effective self-management strategies.

Acknowledgements

The authors would like to acknowledge and thank the participants and PPIEP members who took the time and effort to take part in this research and share their experiences.

References

[1] United Kingdom. Department of Health and Social Care. Major conditions strategy: case for change and our strategic framework [Internet]. London: Department of Health and Social Care; 2023 [cited 2025 August 21]. Available from: https://www.gov.uk/government/publications/major-conditions-strategy-case-for-change-and-our-strategic-framework/major-conditions-strategy-case-for-change-and-our-strategic-framework--2

[2] NHS England and NHS Improvement. The Improving Access to Psychological Therapies (IAPT) Pathway for People with Long-term Physical Health Conditions and Medically Unexplained Symptoms [Internet]. London: NHS England; 2018 [cited 2025 August 21]. Available from: https://www.england.nhs.uk/wp-content/uploads/2018/03/improving-access-to-psychological-therapies-long-term-conditions-pathway.pdf

[3] World Health Organization. Mental health and COVID-19: early evidence of the pandemic's impact: scientific brief, 2nd March 2022 [Internet]. Geneva: World Health Organization; 2022 [cited 2025 August 15]. Available from: https://iris.who.int/bitstream/handle/10665/352189/WHO-2019-nCoV-Sci-Brief-Mental-health-2022.1-eng.pdf?sequence=1

[4] Pearson-Stuttard J, Ezzati M, Gregg EW. Multimorbidity – a defining challenge for health systems. The Lancet Public Health [Internet]. 2019 December [cited 2025 August 12]; 4(12): Article number e599-3600. Available from: https://www.thelancet.com/journals/lanpub/article/PIIS2468-2667(19)30222-1/fulltext

[5] Naylor C, Das P, Ross S, Honeyman M, Thompson J, Gilburt H. Bringing together physical and mental health: a new frontier for integrated care [Internet]. London: The King's Fund; 2016 [cited 2025 August 20]. Available from: https://assets.kingsfund.org.uk/f/256914/x/5fa0a562d8/bringing_together_physical_and_mental_health_march_2016.pdf

[6] Mental Health Foundation. Mental health problems cost UK economy at least GBP 188 billion a year – new research [Internet]. London: Mental Health Foundation; 2022 March 2 [cited 2025 August 18]. Available from: https://www.mentalhealth.org.uk/about-us/news/mental-health-problems-cost-uk-economy-least-gbp-118-billion-year-new-research

[7] World Health Organization. World health statistics 2018: monitoring health for the SDGs, sustainable development goals [Internet]. Geneva: World Health Organization; 2018 [cited 2025 August 17]. Available from: https://www.who.int/docs/default-source/gho-documents/world-health-statistic-reports/6-june-18108-world-health-statistics-2018.pdf

[8] World Health Organization. World report on social determinants of health equity [Internet]. Geneva: World Health Organization; 2025 May 6 [cited 2025 August 17]. Available from: https://www.who.int/publications/i/item/9789240107588

[9] Holland E, Matthews K, Macdonald S, Ashworth M, Laidlaw L, Cheung KSY, et al. The impact of living with multiple long-term conditions (multimorbidity) on everyday life – a qualitative evidence synthesis. BMC Public Health [Internet]. 2024 December 18 [cited 2025 September 1]; 24: Article number 3446. Available from: https://bmcpublichealth.biomedcentral.com/articles/10.1186/s12889-024-20763-8#citeas

[10] Porter T, Ong BN, Sanders T. Living with multimorbidity? The lived experience of multiple chronic conditions in later life. Health (London) [Internet] 2020 November [cited 2025 August 18]; 24(6): 701-718. Available from: https://pubmed.ncbi.nlm.nih.gov/30895825/

[11] Carswell C, Brown JVE, Lister J, Ajjan RA, Anderson SL, Balogun-Katung A, et al. The lived experience of severe mental illness and long-term conditions: a qualitative exploration of service user, carer and health professional perspectives on self-managing co-existing mental and physical conditions. BMC Psychiatry [Internet]. 2022 July 19 [cited 2025 August 17]; 22: Article number 479. Available from: https://doi.org/10.1186/s12888-022-04117-5

[12] Shefer G, Henderson, C, Howard LM, Murray J, Thornicroft G. Diagnostic overshadowing and other challenges involved in the diagnostic process of patients with mental illness who present in emergency departments with physical symptoms – a qualitative study. PLos One [Internet]. 2015 November 4th [cited 2025 August 13]; 9(11): Article number 111682. Available from: https://doi.org/10.1371/journal.pone.0111682

[13] National Health Service. The NHS Long Term Plan [Internet]. London: National Health Service; 2019 January [cited 2025 November 11]. Available from: https://webarchive.nationalarchives.gov.uk/ukgwa/20230418155402/https:/www.longtermplan.nhs.uk/publication/nhs-long-term-plan/

[14] Department of Health and Social Care. Fit for the future: 10 Year Health Plan for England [Internet]. London: Department of Health and Social Care; 2025 July 3rd [cited 2025 August 13]. Available from:

https://assets.publishing.service.gov.uk/media/6888a0b1a11f859994409147/fit-for-the-future-10-year-health-plan-for-england.pdf

[15] van de Velde D, de Zutter F, Satink T, Costa U, Janquart S, Senn D, de Vriendt P. Delineating the concept of self-management in chronic conditions: a concept analysis. BMJ Open [Internet]. 2018 [cited 2025 August 14]; 9(7): Article number e027775. Available from: https://doi.org/10.1136/bmjopen-2018-027775

[16] National Institute for Health and Care Research. Multiple long-term conditions (multimorbidity) and inequality – addressing the challenge: insights from research [Internet]. NIHR Evidence; 2023 September 20 [cited 2025 August 13]. Available from: https://doi.org/10.3310/nihrevidence_59977

[17] Spiers G, Boulton E, Corner L, Craig D, Parker S, Todd, C, Hanratty B. What matters to people with multiple long-term conditions and their carers? Postgraduate Medical Journal [Internet]. 2021 December 17 [cited 2025 August 18]; 99(1169): 159-165. Available from: https://doi.org/10.1136/postgradmedj-2021-140825

[18] Woodward A, Davies N, Walters K, Nimmons D, Stevenson F, Protheroe J, et al. Self-management of multiple long-term conditions: A systematic review of the barriers and facilitators amongst people experiencing socioeconomic deprivation. PLoS One [Internet]. 2023 February 21 [cited 2025 August 17]; 18(2): Article number e0282036. Available from: https://doi.org/10.1371/journal.pone.0282036

[19] World Health Organization. Mental health [Internet]. Geneva: World Health Organization; 2025 October [cited 2026 May 19]. Available from: https://www.who.int/news-room/fact-sheets/detail/mental-health-strengthening-our-response

[20] National Health Service. Low mood, sadness and depression [Internet]. London: National Health Service; 2022 January [cited 2026 May 19]. Available from: https://www.nhs.uk/mental-health/feelings-symptoms-behaviours/feelings-and-symptoms/low-mood-sadness-depression/

[21] National Health Service. Generalised anxiety disorder (GAD) [Internet]. London: National Health Service; 2024 October [cited 2026 May 19]. Available from: https://www.nhs.uk/mental-health/conditions/generalised-anxiety-disorder-gad/

[22] Mental Health Foundation. Self-management of mental ill-health [Internet]. London: Mental Health Foundation; 2022 February [cited 2026 May 19]. Available from: https://www.mentalhealth.org.uk/explore-mental-health/a-z-topics/self-management-mental-ill-health#paragraph-56807

[23] National Institute for Health and Care Excellence. Mental health guidelines [Internet]. London: NICE; 2026 [cited 2026 May 19]. Available from: https://www.nice.org.uk/guidance/indevelopment/gid-hub10005

[24] Smith SM, Wallace E, Clyne B, Boland F, Fortin M. Interventions for improving outcomes in patients with multimorbidity in primary care and community setting: a systematic review. Systematic Reviews [Internet}. 2021 October 20 [cited 2025 August 20]; 10: Article number 271. Available from: https://doi.org/10.1186/s13643-021-01817-z

[25] Breckner A, Roth C, Glassen K, Wensing M. Self-management perspectives of elderly patients with multimorbidity and practitioners - status, challenges and further support needed? BMC Primary Care [Internet] 2021 November 26 [cited 2025 August 20]; 22: Article number 238. Available from: https://doi.org/10.1186/s12875-021-01584-9

[26] Smith L, Simpson G, Holt S, Dambha-Miller H. Digital applications to support self-management of multimorbidity: A scoping review. International Journal of Medical Informatics [Internet]. 2025 October [cited 2025 August 20]; 202: Article number 105988. Available from: https://doi.org/10.1016/j.ijmedinf.2025.105988

[27] Balogun-Katung A, Carswell C, Brown JVE, Coventry P, Ajjan R, Alderson A. Exploring the facilitators, barriers and strategies for self-management in adults living with severe mental illness, with and without long-term conditions: A qualitative evidence synthesis. PLOS One [Internet]. 2021 October 26 [cited 2025 September 5]; 16(10): Article number e0258937. Available from: https://doi.org/10.1371/journal.pone.0258937

[28] Coventry PA, Young B, Balogun-Katung A, Taylor J, Brown JVE, Kitchen C. Determinants of physical health self-management behaviours in adults with serious mental illness: A systematic review. Frontiers in Psychiatry [Internet]. 2021 August 18 [cited 2025 September 5]; 12: Article number 723962. Available from: https://doi.org/10.3389/fpsyt.2021.723962

[29] Beaudin J, Chouinard MC, Hudon E, Hudon C. Factors and strategies influencing integrated self-management support for people with chronic diseases and common mental disorders: A qualitative study of Canadian primary care nurses' experiences. Journal of Advanced Nursing Qualitative [Internet]. 2025 March 10 [cited 025 September 5]; 0: 1-16. Available from: https://doi.org/10.1111/jan.16892

[30] Kappelin C, Sandlund C, Westman J, Wachtler C. Dancing with the patient: a qualitative study of general practitioners' experiences of managing patients with multimorbidity and common mental health problems. BMC Primary Care [Internet].

2023 April 20 [cited 2025 September 5]; 24: Article number 104. Available from: https://doi.org/10.1186/s12875-023-02056-y

[31] MIND. The Big Mental Health Report. London: MIND. 2025 [cited 2025 December 8]. Available from: https://www.mind.org.uk/media/zianu2rn/mind_the-big-mental-health-report_digital4.pdf

[32] United Nations. Transforming our world: the 2030 agenda for sustainable development. New York: United Nations. 2015 [cited 2025 December 8]. Available from: https://sdgs.un.org/2030agenda

[33] World Health Organization and Calouste Gulbenkian Foundation. Policy options on mental health: a WHO-Gulbenkian Mental Health Platform collaboration. Geneva: World Health Organization. 2017 [cited 2025 December 8]. Available from: https://iris.who.int/server/api/core/bitstreams/9d896b3e-78f8-48f5-bcbd-bb2bc8f77c75/content

[34] Morgan HM, Entwistle VA, Cribb A, Christmas S, Owens J, Skea ZC, Watt IS. We need to talk about purpose: a critical interpretative synthesis of health and social care professionals' approaches to self-management support for people with long-term conditions. Health Expectations [Internet] 2016 April 14 [cited 2025 August 12]; 20(2): 243-259. Available from: https://doi.org/10.1111/hex.12453

[35] National Institute for Health and Care Research. Multiple long-term conditions (multimorbidity): making sense of the evidence [Internet]. NIHR Evidence. 2021 March 30 [cited 2025 August 13]. Available from: https://doi.org/10.3310/collection_45881

[36] NHS England. Improving the physical health of people living with severe mental illness [Internet]. London: NHS; 2024 January 10 [updated 2025 May 7th; cited 2025

August 12]. Available from: https://www.england.nhs.uk/long-read/improving-the-physical-health-of-people-living-with-severe-mental-illness/

[37] Smith JA, Flower P, Larkin M. Interpretative Phenomenological Analysis: Theory, Method and Research. London: SAGE Publications; 2009.

[38] Smith JA, Flower P, Larkin M. Interpretative Phenomenological Analysis: Theory, Method and Research. 2nd edition. London: SAGE Publications; 2022.

[39] Creswell JW, Poth CN. Qualitative Inquiry and Research Design: Choosing Among Five Approaches. 5th edition. Los Angeles: SAGE Publications; 2024

[40] Pope C, Mays N. Qualitative Research in Health Care. 4th edition. Chichester: John Wiley and Sons Ltd; 2019.

[41] Ahmed SK. The pillars of trustworthiness in qualitative research. *Journal of Medicine, Surgery, and Public Health [Internet]* 2024 April [cited 2025 November 11]; 2(1). Available from: https://doi.org/10.1016/j.glmedi.2024.100051

[42] Lincoln SY, Guba EG. Naturalistic inquiry. Thousand Oaks, California: Sage; 1985

[43] Tong A, Sainsbury P, Craig J. Consolidated criteria for reporting qualitative research (COREQ): a 32-item checklist for interviews and focus groups. International Journal for Quality in Health Care [Internet]. 2007 September 14 [cited 2025 August 13]; 19(6): 349-357. Available from: https://doi.org/10.1093/intqhc/mzm042

[44] Green J, Thorogood N, Melendez-Torres GJ. Qualitative Methods for Health Research. 5th edition. Los Angeles: SAGE Publications; 2025.

[45] Peat G, Rodriguez A, Smith J. Interpretative phenomenological analysis applied to healthcare research. Evidence Based Nursing [Internet] 2019 January [cited 2025

November 11]; 22(1): 7-9. Available from: https://doi.org/10.1136/ebnurs-2018-103017

[46] Malterud K, Siersma V, Guassora AD. Information power: sample content and size in qualitative studies. In: Camid PM, editor. Qualitative research in psychology: Expanding perspectives in methodology and design. 2nd edition. Washington: American Psychological Association, 2021. 67-81 p.

[47] Saunders B, Sim J, Kingstone T, Baker S, Waterfield J, Bartlam B, et al. Saturation in qualitative research: exploring its conceptualization and operationalization. Quality and Quantity [Internet] 2017 September 14 [cited 2025 November 7]; 52(4): 1893-1907. Available from: https://doi.org/10.1007/s11135-017-0574-8

[48] UK Public General Acts. Data Protection Act. 2018 [cited 2025 August 13]. Available from: https://www.legislation.gov.uk/ukpga/2018/12/contents/enacted

[49] Staniszewska S, Brett J, Simera L, Seers K, Mockford C, Goodlad S, et al. GRIPP2 reporting checklists: tools to improve reporting of patient and public involvement in research. British Medical Journal [Internet]. 2017 April 2nd [cited 2025 August 17]; 358 Article number 3453. Available from: https://doi.org/10.1136/bmj.j3453

[50] Allen RES, Wiles JL. A rose by any other name: participants choosing research pseudonyms. Qualitative Research in Psychology [Internet]

[51] Charlick S, Pincombe J, McKellar L, Fielder A. Making Sense of Participant Experiences: Interpretative Phenomenological Analysis in Midwifery Research. International Journal of Doctoral Studies [Internet]. 2016 January [cited 2025 November 17]; 11: 205-216. Available from: https://doi.org/10.28945/3486

[52] Shinebourne P. The theoretical underpinnings of Interpretative Phenomenological Analysis (IPA). Existential Analysis [Internet]. 2011 [cited 2025 November 17]: 22(1): 16-31. Available from: https://psycnet.apa.org/record/2011-05339-003

[53] Willig C, Rogers WS, Eatough V, Smith JA. Interpretative Phenomenological Analysis. In: Willig C, Rogers WS, editors. The SAGE Handbook of Qualitative Research in Psychology. 2nd edition. Los Angeles: SAGE Publications Ltd; 2017 [cited 2025 November 17]. p. 193-211. Available from: https://doi.org/10.4135/9781526405555.n12

[54] Lloyd N, Hyett N, Kenny A. To Member Check or not to Member Check? An Evaluation of Member Checking in an Interpretative Descriptive Study. International Journal of Qualitative Methods [Internet]. 2024 November 15 [cited 2025 August 12]; 23. Available from: https://doi.org/10.1177/160940692413013

[55] Smith, J. A. (2015) *Qualitative Psychology: A practical guide to research methods*. 3rd edition. London: SAGE Publications.

[56] World Medical Association [WMA]. WMA Declaration of Helsinki–Ethical Principles for Medical Research Involving Human Subjects. 2013 [cited 24 February 2026]. Available: https://www.wma.net/policies-post/wma-declaration-of-helsinki-ethical-principles-for-medical-research-involving-human-subjects/

[57] Smith JA, Pietkiewicz, I. A practical guide to using Interpretative Phenomenological Analysis in qualitative research psychology. Psychological Journal [Internet]. 2014 [cited 2025 August 12]; 20(1): 7-14. Available from: https://doi.org/10.14691/CPPJ.20.1.7

[58] Woodward A, Nimmons D, Davies, N, Walters K, Stevenson FA, Protheroe J, et al. A qualitative exploration of the barriers and facilitators to self-managing multiple long-term conditions amongst people experiencing socioeconomic deprivation. Health Expectations [Internet]. 2024 April 16 [cited 2025 August 13]; 27(2) Article number 14046. Available from: https://doi.org/10.1111/hex.14046

[59] Wyke E, Baxter ER, Chew-Graham CA, Woodward A, Walters K, Davies N, et al. Managing multiple long-term conditions in people experiencing socioeconomic deprivation: a qualitative study of primary care perspectives. British Journal of General Practice [Internet]. 2026 May [cited 2026 June 1]; 76(766): 366-375. Available from: https://doi.org/10.3399/BJGP.2025.0361

[60] Coventry PA, Fisher L, Kenning C, Bee P, Bower P. Capacity, responsibility, and motivation: a critical qualitative evaluation of patient and practitioner views about barriers to self-managment in people with multimorbidity. BMC Health Services Research [Internet]. 2014 October 14 [cited 2026 June 1]; 14 Article number 536. Available from: https://doi.org/10.1186/s12913-014-0536-y

[61] Gobeil-Lavoie A-P, Chouinard M-C, Danish A, Hudon C. Characteristics of self-management among patients with complex health needs: a thematic analysis review. BMC Open [Internet]. 2019 [cited 2025 August 14]; 9(5) Article number 028344. Available from: https://doi.org/10.1136/bmjopen-2018-028344

[62] Joseph L, Krishnan A, Lekha TR, Sasidharan N, Thulaseedhara JV, Valamparampil MJ, et al. Experiences and challenges of people living with multiple long-term conditions in managing their care in primary care settings in Kerala, India: A qualitative study. PLoS One [Internet]. 2024 June 13 [cited 2025 August 13]; 19(6)

Article number 0305430. Available from: https://doi.org/10.1371/journal.pone.0305430 https://doi.org/10.1111/hex.14046

[63] Mooney O, Lambert V, Gallagher P. Self-management of Long-Term Physical Conditions During Emerging Adulthood: A Systematic Review. Emerging Adulthood [Internet]. 2023 September 29 [cited 2025 September 10]; 11(6): 1446-1468. Available from: https://doi.org/10.1177/21676968231203284

[64] Corp N, Bray L, Chew-Graham CA, Polidano K, Fisher T, Garmer AD, et al. Self-directed self-management interventions to prevent or address distress in young people with long-term physical conditions: A rapid review. Health Expectations [Internet]. 2023 August 2 [cited 2025 September 10]; 26(6): 2164-2190. Available from: https://doi.org/10.1111/hex.13845

[65] Wilson C, Stock J. The impact of living with long-term conditions in young adulthood on mental health and identity: What can help? Health Expectations [Internet]. 2019 July 25 [cited 2025 September 10]; 22(5): 1111-1121. Available from: https://doi.org/10.1111/hex.12944

[66] Franklin M, Willis K, Lewis S, Smith L. Chronic condition self-management is a social practice. Journal of Sociology [Internet]. 2021 August 26 [cited 2025 September 10]; 59(1): 215-231. Available from: https://doi.org/10.1177/14407833211038059

[67] Rijken M, Hujala A, van Ginneken E, Melchiorre MG, Groenewegen P, Schelevis F. Managing multimorbidity: Profiles of integrated care approaches targeting people with multiple chronic conditions in Europe. Health Policy [Internet]. 2018 January [cited 2025 September 10]; 122(1): 44-52. Available from: https://doi.org/10.1016/j.healthpol.2017.10.002

[68] Whitty CJM, MacEwen C, Goddard A, Alderson D, Marshall M, Calderwood C, et al. Rising to the challenge of multimorbidity. BMJ [Internet]. 2020 January 6 [cited 2025 September 10]; 368: Article Number I6964. Available from: https://doi.org/10.1136/bmj.l6964

[69] Carswell C, Coventry PA, Brown JVE, Alderson SL, Double K, Gilbody S, et al. Development of a Supported Self-management Intervention for People With Severe Mental Illness and Type 2 Diabetes: Theory and Evidence-Based Co-design Approach. 2023 [cited 2025 August 15]; 12(25) Article number 43597. Available from: https://doi.org/10.2196/43597

[70] Reilly S, Olier I, Planner C, Doran T, Reeves D, Ashcroft DM, Gask L, Kontopantelis E. Inequalities in physical comorbidity: a longitudinal comparative cohort study of people with severe mental illness in the UK. BMJ Open [Internet]. 2015 December 15 [cited 2025 August 13]; 15(5) Article number 3009010. Available from: https://doi.org/10.1136/bmjopen-2015-009010

[71] Sattoe JNT, van Staa A, Hilberink SA. Self-management of Young People with Chronic Conditions: A strength-based approach for empowerment and support. Switzerland: Springer Nature, 2021.

[72] Frantz J, Schoop L, Rhoda A. Self-management in Chronic Illness: Principles, Practice and Empowerment Strategies for Better Health. Switzerland: Springer Nature, 2021.

[73] World Health Organization. Global report on ageism [Internet]. Geneva: World Health Organization; 2021 [cited 2025 September 10]. Available from: https://iris.who.int/bitstream/handle/10665/340208/9789240016866-eng.pdf?sequence=1

[74] National Institute for Health and Care Excellence. Multimorbidity: clinical assessment and management [Internet]. London: NICE; 2016 [cited 2025 August 14]. [NG56]. Available from: https://www.nice.org.uk/guidance/ng56

[75] Moody L, Wood E, Needham A, Booth A, Jimenez-Aranda A, Tindale W. Identifying individual enablers and barriers to the use of digital technology for the self-management of long-term conditions by older adults. Journal of Medical Engineering & Technology [Internet] 2022 June 24 [cited 2026 May 19]; 46(6): 448-461. Available from: https://doi.org/10.1080/03091902.2022.2089249

[76] National Institute for Health and Care Excellence. Older people with social care needs and multiple long-term conditions [Internet]. London: NICE; 2015 [cited 2025 August 14]. [NG22]. Available from: https://www.nice.org.uk/guidance/ng22

[77] Nguyen TNM, Whitehead L, Saunders R, Dermody G. Systematic review of perception of barriers and facilitators to chronic disease self-management among older adults: Implications for evidence-based practice. Worldviews on Evidence-Based Nursing [Internet] 2022 January 15 [cited 2025 August 15]; 19(3): 191-200. Available from: https://doi.org/10.1111/wvn.12563

[78] National Health Service. A blueprint for Young Future hubs [Internet]. London: NHS; 2025 March [cited 2025 November 27]. Available from: https://www.youthaccess.org.uk/sites/default/files/documents/A%20blueprint%20for%20Young%20Futures%20hubs%20by%20Youth%20Access%20and%20partners.pdf

[79] Hidden Disabilities Sunflower Scheme Limited. About the Sunflower [webpage on the internet]. [cited 2025 December 6] Available from: https://hdsunflower.com/about-hidden-disabilities-sunflower

[80] Ahtoy P. Behind the hidden struggles linked to invisible disabilities. Understanding their impact to increase mental health awareness for enhanced professional care. Ethics, Medicine and Public Health [Internet]. 2024 [cited 2025 December 8]; 32: Article number 101016. Available from: https://doi.org/10.1016/j.jemep.2024.101016

[81] Clement S, Schauman O, Graham T, Maggioni F, Evans-Lacko S, Bexborodovs N, et al. What is the impact of mental health-related stigma on help-seeking? A systematic review of quantitative and qualitative studies. Psychological Medicine [Internet]. 2015 [cited 2025 December 8]; 45(1):11-27. Available from: https://doi.org/10.1017/S0033291714000129

[82] Thomas V, Ahmed H, Lindsay S. Unravelling the Complexities of Workplace Disclosure Among Persons with Non‐Visible Disabilities and Illnesses: A Qualitative Meta‐Ethnography. Journal of Occupational Rehabilitation [Internet]. 2022 [cited 2025 December 8]; 32(1): 538-563. Available from: https://doi.org/10.1007/s10926-022-10023-0

[83] Moriña A. When what is unseen does not exist: disclosure, barriers and supports for students with invisible disabilities in higher education. Disability & Society [Internet]. 2024 [cited 2025 December 8]; 39(4): 914-932. Available from: https://doi.org/10.1080/09687599.2022.2113038

[84] Morgan HM, Entwistle VA, Cribb A, Christmas S, Owens J, Skea ZC, et al. We need to talk about purpose: a critical interpretive synthesis of health and social care professionals' approaches to self-management support for people with long-term conditions. Health Expectations [Internet] 2016 April 14 [cited 2025 December 8]; 20(2): 243-259. Available from: https://doi.org/10.1111/hex.12453

[85] Hajat C, Stein E. The global burden of multiple chronic conditions: A narrative review. Preventative Medicine Reports [Internet]. 2018 [cited 2025 December 8]; 12(1): 284-293. Available from: https://doi.org/10.1016/j.pmedr.2018.10.008

[86] Mitchell AJ, Hardy S, Shiers D. Parity of esteem: addressing the inequalities between mental and physical healthcare. BJPsych Advances [Internet]. 2018 January 2 [cited 2025 December 8]; 23(3): 196-205. Available from: https://doi.org/10.1192/apt.bp.114.014266

[87] Department of Health and Social Care. Premature mortality in adults with severe mental illness (SMI). London: GOV.UK; 2018 April 19 [cited 2025 December 8]. Available from: https://www.gov.uk/government/publications/premature-mortality-in-adults-with-severe-mental-illness/premature-mortality-in-adults-with-severe-mental-illness-smi

[88] Lowther-Payne HJ, Ushakova A, Beckwith A, Liberty C, Edge R, Lobban F. Understanding inequalities in access to adult mental health services in the UK: a systematic mapping review. BMC Health Services Research [Internet]. 2023 [cited 2025 December 8]; 23(1): Article number 1042. Available from: https://doi.org/10.1186/s12913-023-10030-8

[89] Wagner EH. Chronic disease management: what will it take to improve care for chronic illness? Effective Clinical Practice [Internet] 1998 September [cited 2025 August 17]; 1(1): 2-4. Available from: https://pubmed.ncbi.nlm.nih.gov/10345255/

[90] Bandura A. Self-efficacy: Toward a unifying theory of behavioral change. Advances in Behaviour Research and Therapy [Internet]. 1978 [cited 2025 August 17]; 1(4): 139-161. Available from: https://doi.org/10.1016/0146-6402(78)90002-4

[91] Schoemaker CG, van Geelen SM, Allewijn M, Fernhout M, van Vliet R, Wulffraat N. Self-management support for young people living with fluctuating chronic diseases. Archives of Disease in Childhood [Internet]. 2022 January 10 [cited 2025 August 17]; 107(7): 658-659. Available from: https://doi.org/10.1136/archdischild-2021-323118

[92] Paterson BL. The Shifting Perspectives Model of Chronic Illness. Journal of Nursing Scholarship [Internet] 2004 April 23 [cited 2025 August 17]; 33(1): 21-26. Available from: https://doi.org/10.1111/j.1547-5069.2001.00021.x

[93] Hardman R, Begg S, Spelten E. What impact do chronic disease self-management support interventions have on health inequity gaps related to socioeconomic status: a systematic review. BMC Health Services Research [Internet] 2020 February 27 [cited 2025 August 14]; 20: Article number 150. Available from: https://bmchealthservres.biomedcentral.com/articles/10.1186/s12913-020-5010-4

[94] Schiøtz ML, Stockmarr A, Høst D, Glümer C, Frølich A. Social disparities in the prevalence of multimorbidity. A register-based population study. BMC Public Health [Internet] 2017 May 10 [cited 2025 August 17]; 17(1): 422. Available from: https://doi.org/10.1186/s12889-017-4314-8

Supporting Information

S1 Checklist. Consolidated Criteria for reporting qualitative studies (COREQ): 32-item checklist.

S2 Checklist. GRIPP2-SF Reporting Checklist.

S1 Fig. Ethical approval.

S2 Fig. Ethical approval.

S1 Table. Interview topic guide.

S2 Table. Stages of Interpretative Phenomenological analysis (extracts from Rosemary’s transcript).

S3 Table. Participant contribution to Group Experiential Themes (GETs)

S1_Fig

Click here to access/download
Supporting Information
S1_Fig.tif

S2_Fig

Click here to access/download
Supporting Information
S2_Fig.tif

Click here to access/download
Supporting Information
S1_Checklist.docx

Click here to access/download
Supporting Information
S2_Checklist.docx

Click here to access/download
Supporting Information
S1_Table.docx

Click here to access/download
Supporting Information
S2_Table.docx

Click here to access/download
Supporting Information
S3_Table.docx

"I want to take the power back": An interpretative phenomenological analysis of self-management in coexisting physical and mental long-term conditions

Catherine Lodwick [1*], Leire Ambrosio [1,2], Mari Carmen Portillo [1,2]

[1] School of Health Sciences, University of Southampton, Southampton, UK

[2] National Institute for Health and Care Research (NIHR) Applied Research Collaboration Wessex, Southampton, UK

* Correspondence concerning this article should be addressed to Catherine Lodwick.

Email: cel1g20@soton.ac.uk (CL)

Authors' Contributions

Catherine Lodwick: Conceptualization, Data curation, Formal analysis, Funding acquisition, Investigation, Methodology, Project administration, Writing – original draft, Writing – reviewing & editing

Leire Ambrosio: Funding acquisition, Supervision, Writing – review & editing

Mari Carmen Portillo: Conceptualization, Formal Analysis, Supervision, Writing – review & editing

Abstract

Introduction: Individuals living with both physical and mental health conditions face compounding barriers to self-management. These challenges arise from complex care needs, interactions between long-term conditions symptoms and inequalities resulting in a high treatment burden. While previous research on coexisting conditions has largely focused on those with severe mental illness, there remains an important gap in understanding the factors that help or hinder self-management among adults living with coexisting physical and common mental health conditions (anxiety/depression) in the community.

Objective: This study aims to explore the facilitators and barriers to self-management for adults living with a physical long-term condition and coexisting anxiety or depression in the community.

Methods: A qualitative study with in-depth semi-structured interviews was conducted between May and August 2025. Eight adults living with coexisting physical and mental health conditions in the UK participated. Interview data was recorded, transcribed and analysed using Interpretative Phenomenological Analysis.

Results: Three main themes were generated; (1) Moving beyond sink or swim: scaffolding support to self-manage (2) Self-managing: a choice or systemic exclusion (3) Unseen struggles: learning to live with my long-term conditions. Participants described the complex, often unsupported reality of self-managing coexisting long-term conditions, where many felt forced to integrate self-management into their lives due to systemic gaps and negative interactions with healthcare professional. Their experiences demonstrated an apparent hinderance to self-management, with age, condition visibility and identity struggles creating compounding barriers. Therefore, a

holistic scaffolded support approach promoting integrated care was suggested to meet both their health and social needs.

Conclusions: The demands of living with coexisting conditions presented substantial barriers to self-management. The study found younger age to be a central inequality within self-management support, which crucially must be addressed by policymakers and healthcare professionals in future research to prevent a widening generational gap in sustainable self-management support options.

Our study provides insight into self-management facilitators and barriers among adults living with coexisting physical-mental health long-term conditions, highlighting substantial barriers to self-management and the need for person-centred, flexible, integrated support. The findings emphasise the need for new care models reflecting evolving presentations and domains of coexisting conditions.

Formatted: Line spacing: Double

~~1.~~ Introduction

In recent decades, the burden of living with long-term conditions (LTCs) has increased markedly. In the United Kingdom one in four adults, equating to 15 million people, live with at least two LTCs which are lifelong and cannot be cured [1]. Alongside this, NHS England and Improvement [2] estimate 30% of those living with LTCs have a coexisting mental health condition. It is plausible this figure has continued to rise, particularly as the World Health Organization [3] reported a 25% global prevalence increase in anxiety and depression following the COVID-19 pandemic thus demonstrating their expanding contribution to the global disease burden, highlighting the significant public health impact of coexisting physical and mental LTCs.

The growth in prevalence of coexisting conditions has far reaching implications not only for individuals but also their families and the wider health and social care systems [4]. In the UK, The Kings Fund [5] estimated the collective cost of coexisting physical and mental LTCs to NHS England to be more than £11 billion annually, with the Mental Health Foundation [6] finding a further £88 billion attributed to loss of productivity. Whilst there is currently no global consensus on the economic impact of coexisting LTCs, the World Health Organization [7,8] recognises that they significantly drive early mortality, raise perceived and actual treatment burden, and reduce productivity. All these factors have their own profound fiscal implications which drive long-term financial strain on national and global public services. Yet beyond the financial costs, living with coexisting physical and mental LTCs can be challenging for the individual as it creates complex, multidimensional care needs due to inequalities and condition interactions. Ultimately the interplay from coexisting

conditions may cause poor care adherence, psychological distress and social isolation, which when combined lead to a significantly lower quality of life and higher healthcare utilisation compared to those living with a single LTC [9,10]. ~~Notwithstanding, it is essential to highlight for this population not all care experiences are positive with many individuals' reporting stigma, diagnostic overshadowing and service fragmentation.~~ Notwithstanding, it is essential to highlight for this population not all care experiences are positive with many individuals' reporting stigma, service fragmentation, and diagnostic overshadowing whereby a service users' symptoms may be attributed to their mental health condition overlooking possible medical causes [11]. This disruption to care adversely impacts their wellbeing and trust in health and social care professionals [12].

In response to these challenges, there has been growing emphasis across clinical policies, practice and public health. Most recently observed in the NHS 10 Year plan, which is deeply focused on empowering individuals to be more active in managing their own health and wellbeing within community settings [1,13,14]. An important pillar of this plan focuses on facilitating the widespread adoption of digital tools, personalised care plans and self-management initiatives among service users and providers, which aligns directly with this study's aim. Self-management refers to a combination of strategies from medication management, clinical treatments and behavioural changes to promote good health outcomes and improve one's quality of life [15]. Although self-management is common amongst the general population, approaches used do not always account for the bidirectional relationship and specific needs of those living with coexisting physical and mental LTCs which produce a spectrum of challenges and contributes to widening health and social inequalities [16-18].

The World Health Organization define mental health as a state of mental wellbeing which enables individuals to cope with life stressors, learn and work well and contribute to their community [19]. Common mental ill health symptoms including low mood, anxiety, fatigue, and difficulties with motivation and concentration may reduce an individual's capacity to self-manage day-to-day aspects of LTCs, including medication adherence, symptom monitoring, lifestyle modification, and engagement with healthcare services [20,21]. To manage these challenges alongside physical LTC, individuals may establish routines, use self-regulation and coping techniques, and draw on both informal support from friends and family and formal support from health and social care professionals [22]. Formal support for anxiety and depression may involve psychological therapies and pharmacological treatments; however, variability in their accessibility, continuity, and perceived effectiveness may further affect sustained self-management behaviours [23]. As recognised in the NHS Fit for the Future [14] plan, these obstacles highlight the need for integrated and person-centred care models which address both physical and mental health needs simultaneously, support continuity across services and recognise the interaction between coexisting LTCs.

Despite an increasing evidence-based on self-management interventions and support programmes for LTCs, the focus primarily remains on single-disease models, physical health conditions with clear biomedical markers and severe mental illnesses [24-26~~19-21~~]. While Balogun-Katung et al. [27~~22~~] and Coventry et al. [28~~23~~] have begun to broadly examine barriers and enablers to self-managing coexisting LTCs, their research remains centred on those living with severe mental illness such as schizophrenia and bipolar disorder. In contrast, recent qualitative studies by Beaudin et al. [29~~24~~] and Kappelin et al. [30~~25~~] focus on common mental health

conditions although from the perspective of healthcare professionals. This research illustrate that self-management support continues to be delivered in silos, despite acknowledging coexisting conditions, with practice continuing to show favour towards physical LTCs. Despite the recognition of integrated care in both international policies and guidelines, including The Big Mental Health Report [31~~26~~] Major Conditions Strategy [1], UN Sustainable Development Goals [32~~27~~], and WHO Policy on Mental Health [33~~28~~]. Their objectives are explicit, stating the need to embed mental health support within physical health services and vice versa, with the aspiration to deliver holistic and coordinated care, yet this clearly remains a persistent challenge.

The current care model does not capture coexisting mental LTCs, considering them as secondary concerns rather than integral components of LTC self-management. Therefore, the way living with coexisting LTCs impacts an individual's systemic, relational and personal contexts are insufficiently understood [34-36~~29-31~~]. As a result, there continues to be a limited awareness of how individuals self-manage the multidimensional and intersecting demands of coexisting physical LTCs and anxiety/depression. This qualitative study offers originality by aiming to explore facilitators and barriers to self-management among adults living with both a physical LTC and anxiety/depression in the community. By addressing this critical knowledge gap, the present study has the potential to inform the design of a person-centred and contextually responsive self-management interventions, thereby, contributing to the reduction of health and social inequalities and enhancing the health and wellbeing outcomes for this underserved population.

Materials and Methods ~~2. Material and methods~~

Study Design ~~2.1. Design~~

This was a qualitative study with interpretative phenomenological analysis (IPA) focused on the self-management experiences of adults living with coexisting physical LTCs and anxiety/depression. The IPA paradigm was deemed appropriate for this subject opposed to other broader qualitative inquiry approaches as its grounding in hermeneutic phenomenology aims to gain an in-depth understanding of an individual's lived experience, alongside its idiographic nature, enabling rigorous interpretation on the meaning of an individual's experience [37,38~~32,33~~]. The interpretative stance of IPA facilitated a double hermeneutic process, an approach more congruent with the study's phenomenological aim opposed to the systematic abstraction of grounded theory or observational immersion of ethnography, which was considered especially relevant for this study [39,40~~34,35~~]. By promoting the researchers to deeply and systematically engage with participant's realities and sense-making, this study's design choice strengthens its trustworthiness [41,42~~36,37~~]. To enhance the quality and rigor of results, the COREQ checklist created by Tong, Sainsbury, and Craig [43~~38~~] was used and can be found in S1 Checklist.

~~2.2.~~ Sampling, study participants, and recruitment

~~Aligning with IPA practices, purposeful sampling was applied as it identifies individuals or groups with knowledge or experiences of a specific phenomenon, thus for this study adults self-managing coexisting physical LTCs with anxiety and/or depression [39,40]. Participants were considered eligible to participate if they lived with coexisting physical LTCs and anxiety and/or depression, lived in Southeast~~

~~England, were aged 18 and over. Those living in inpatient or residential settings were excluded as their self-management may be influenced or carried out by others. Sampling was congruent with the data analysis process and continued until no new ideas emerged; inferring information power had been achieved [41,42].~~

Aligning with IPA practices, purposeful sampling was applied as it identifies individuals or groups with knowledge or experiences relevant to the phenomenon of interest; thus, this study focused on adults self-managing coexisting physical LTCs alongside anxiety and/or depression [44,45]. Participants were considered eligible to participate if they had a history of coexisting physical LTCs and anxiety and/or depression, lived in Southeast England, were aged 18 and over. Participants' LTCs were self-reported to enhance accessibility and reduce participation barriers, including limited feasibility and time to obtain or verify clinical information with healthcare providers. Those living in inpatient or residential settings were excluded as their self-management may be influenced or carried out by others. Sampling was congruent with the data analysis process and continued until no new ideas emerged, inferring information power had been achieved [46,47].

Recruitment for the study commenced on the 3rd of May 2025 and concluded on the 31st of July 2025 and occurred using three approaches. Firstly, recruitment posters were displayed in the Hampshire area. Secondly, social media channels (Instagram) advertised the research study and finally, two charitable organisations (Age UK, Communicare) were contacted, obtaining managerial approval, to publicise recruitment materials to members of their network. Potentially interested participants initially emailed the primary author using contact information provided on the recruitment poster. Upon expressing interest, participants completed a screen questionnaire to ensure eligibility. Where all criteria were met, participant information

sheets and consent forms were sent to participants via email. ~~were sent out.~~ Participants returned informed written consent forms, if they wished to proceed, via SafeSend software to ensure secure transfer of personal information. This was followed by the participant and the author arranging a mutually convenient interview date and time.

~~2.3.~~ Data Collection

Participants were individually interviewed using a semi-structured format with open-ended questions, designed to encourage participants to provide a rich account of their lived experiences with minimal direction from the researcher. Following consultations with the PPIEP group and due to pragmatic considerations, interviews were conducted on Microsoft Teams which were audio and video recorded. The interview topic guide, found in S1 Table, was generated based on the existing literature and feedback from MCP and the study's PPIEP group. All interviews were conducted between May and August 2025 and lasted between 40-70 minutes. Recordings were securely stored on a password protected computer in accordance with the Data Protection Act 2018 [48~~43~~]. The researchers had no therapeutic relationship with study participants.

~~2.4.~~ Participation and Public Involvement, Engagement and Participation (PPIEP)

Two PPIEP members were consulted throughout all stages of the research process to ensure the study remained grounded in service user priorities with lived experience of coexisting LTCs, enhancing rigor and credibility. One member was a W~~w~~hite mental health nurse living with anxiety, depression and several physical

LTCs and the second member was a service user from the BAME (Black, Asian, and Minority Ethnic) community living with urinary and reproductive LTCs alongside anxiety and depression, which provided a range of knowledge and experiences. Meetings were held with the lead researcher (CL) and PPIEP members both in-person and online depending on the individual's availability. Both PPIEP members contributed to the study from designing the scope and direction, topic guide development, theme naming and refinement. Key contributions from discussions between PPIEP members and CL included providing guidance on interview settings as discussions indicated that while in-person interviews may have further supported rapport-building, they were less feasible due to logistical constraints and the fluctuating nature of living with LTCs. As such, an online approach was chosen to accommodate participants' varying needs and circumstances whilst allowing a more personable and interactive experience than telephone calls, which was crucial given the sensitive nature of discussing self-management experiences. Their extended contributions and engagement have been evaluated using the GRIPP2-SF as recommended by Staniszewska et al. [49~~44~~] to ensure reporting transparency, see S2 Checklist.

~~2.5.~~ Data Analysis

Each interview was transcribed verbatim with all personal identifying information removed and pseudonymised, with pseudonyms chosen by participants when arranging the interviews ~~chosen by participants themselves~~ [50~~45~~]. Data analysis began as soon as each interview was transcribed, employing the six step IPA process by Smith et al. [38~~33~~]. No digital software was used to analyse the data as Smith et al. [38~~33~~] encourage use of the paper-pencil method to promote closeness and deep engagement when analysing and critically interpreting the data.

~~Initially, the transcripts were printed and numbered with continuous lines. Transcripts were examined one-by-one to gain a clear picture of the individual's lived self-management experience. The inductive analysis process began by creating exploratory notes focusing on three distinct categories: descriptive, linguistic and conceptual, marked by corresponding coloured pens on the right-hand side of the margins. From the exploratory notes, experiential statements were constructed, written on the left-hand side of the margins, summarising the transcription as well as exploratory notes. The researcher then cut the transcript into these experiential statements, accompanied by the corresponding transcript lines and exploratory notes. These elements were then laid out to systematically identify connections or patterns across statements to generate Personal Experiential Themes (PETs). See S2 Table for further illustration.~~

Initially, the transcripts were printed and numbered with continuous lines. Transcripts were examined one-by-one to gain a clear picture of the individual's lived self-management experience. The inductive analysis process began by creating exploratory notes focusing on three distinct categories: descriptive, linguistic, and conceptual, marked by corresponding coloured pens on the right-hand side of the margins. Descriptive comments focused on the participants’ accounts of key events, experiences, and emotions. Linguistic notes attended to the participants' use of language, such as tone, repetition, pauses, metaphors, emphasis, or pronoun use. Conceptual codes involved a more interpretative level of engagement, exploring underlying assumptions, possible meanings and connections between participant's experiences and broader psychological or experiential concepts. From the exploratory notes, experiential statements were constructed, written on the left-hand side of the margins, summarising the transcription as well as exploratory notes. This

process involved synthesising descriptive, linguistic, and conceptual observations into concise interpretative statements which captured the lived meaning of participant's experiences. The researcher then cut the transcript into these experiential statements, accompanied by the corresponding transcript lines and exploratory notes. These experiential statements were then laid out to visually facilitate a systematic exploration of connections, similarities, differences, and patterns across the data. Through this iterative process, related statements were clustered together to generate Personal Experiential Themes (PETs), capturing how individual participants understood and made meaning of their lived experiences. See S2 Table for further illustration of the process of developing exploratory notes, into experiential statements and subsequent themes.

This process was applied to all eight transcripts after which a summary document was produced for each participant's PETs. Following refinement of all PETs, their summaries were cut and reordered to enable thematic comparison through exploration of patterns and relationships, subsequently informing the development of Group Experiential Themes (GETs). The findings of this study underwent a rigorous analysis and interpretation process, thus resulting in the creation of analytical themes which represent a synthesis of participant accounts, interpretative engagement, and theoretically informed understandings [46]. Following the theoretical underpinnings of IPA, the GETs were increasingly subject to higher levels of abstraction, by applying a theory-informed interpretative stance, to capture both the experiential depth of participant’s lived experiences and the underlying significance of the studied phenomena, further aiding the situation of the current study into existing scholarship [47,48]. To ensure analytical robustness two of the authors, CL and MCP, met regularly to discuss and revise the GETs and subthemes.

~~Member checking was not employed as a validation strategy reflecting recent discourse questioning its suitability within IPA [49]. Instead, credibility was enhanced using peer debriefing, audit trails, and the inclusion of thick descriptions [36,50].~~

This process was applied to all eight transcripts after which a summary document was produced for each participant's PETs. Following refinement of all PETs, theme summaries were reorganised to enable comparison across participants and exploration of patterns and relationships between accounts. This iterative process informed the development of Group Experiential Themes (GETs), representing shared experiential meanings across participants, and were developed when a theme was present in at least half of the accounts. The findings of this study underwent a rigorous analysis and interpretation process, thus resulting in the creation of analytical themes which represent a synthesis of participant accounts, interpretative engagement, and theoretically informed understandings [51]. Consistent with the theoretical and interpretative underpinnings of IPA, the GETs were examined at increasing levels of abstraction through a theory-informed interpretative lens. The aim of this process was not to generate formal theory, but to deepen understanding of participants lived experience and position the findings in relation to existing scholarship [55,53]. To ensure analytical robustness, two of the authors (CL and MCP) corresponded monthly to discuss and revise the GETs and subthemes. Member checking was not employed as a validation strategy reflecting recent discourse questioning its suitability within IPA [54]. Instead, credibility was enhanced using peer debriefing, audit trails, and the inclusion of thick descriptions [41,55].

Formatted: Normal

Formatted: Font: Not Bold, English (United States)

~~2.6.~~ Ethical Considerations

Ethical approval was obtained from the University of Southampton Ethics Committee and the Research Integrity and Governance offices under the reference ERGO 102445, see S1 and S2 Fig. As this research study was unfunded, no financial or material compensation was provided to participants. All the research conforms to the ethical principles for medical research on human beings set out in the Declaration of Helsinki [56~~51~~].

~~3.~~ Results

~~3.1.~~ Study participants

A total of ten potential participants contacted the researcher (CL). However, one did not complete the follow-up, and another withdrew at interview stage. Whilst the concept of information power influenced the overall sampling approach, only eight participants were interviewed and included in the final analysis of this study [46~~41~~]. Despite this, the dataset was deemed sufficient to support meaningful analysis commensurate with IPA philosophy which prioritises depth of insights opposed to frequency to preserve the nuanced interpretation which IPA demands. The characteristics of included participants (Table 1) consisted of seven women and one man, aged between 18 and 34, with physical LTCs encompassing a scope of body systems from hepatic, gastrointestinal, reproductive, musculoskeletal and the nervous system. This small sample is common within IPA studies aligning with recommended ranges of four to ten participants, allowing for more in-depth

interpretation opposed to superficial understanding which may accompany a more diverse sample [5752].

Table 1: Characteristics of interview participants

Participant	Gender	Age	Physical LTCs	Mental LTCs
Rosemary	Female	25-34	Non-Alcoholic Fatty Liver Disease (NAFLD), Irritable Bowel Syndrome (IBS)	Anxiety and Depression
Grace	Female	18-24	Scoliosis, Irritable Bowel Syndrome (IBS)	Anxiety and Depression
Poppy	Female	18-24	Hypermobile Ehlers-Danlos Syndrome (hEDS), Chronic fatigue syndrome (CFS), Chronic migraines	Anxiety and Depression
Ruby	Female	18-24	Chronic constipation, Irritable Bowel Syndrome (IBS), Hypermobility	Anxiety and Depression
Oskar	Male	18-24	Gout	Anxiety
Ashley	Female	18-24	Hypermobile Ehlers-Danlos Syndrome (hEDS), Postural Orthostatic Tachycardia Syndrome (PoTS)	Anxiety and Depression
Laticia	Female	18-24	Irritable Bowel Syndrome (IBS)	Anxiety and Depression
Simone	Female	18-24	Endometriosis, Adenomyosis	Anxiety

Names included are pseudonyms to maintain anonymity.

~~3.2.~~ Themes and subthemes

The analysis process revealed high levels of convergence across the eight interviews which produced three Group Experiential Themes centered on the facilitators and barriers encountered when self-managing coexisting physical and mental LTCs. The themes alongside their associated sub-themes are detailed in

Table 2. Furthermore, each participant's contribution to the development of GETs is illustrated in S3 Table.

Table 2: Description of themes and subthemes

Theme	Description of themes	Subthemes
Moving beyond sink or swim: scaffolding support to self-manage	Articulated the experiences of self-managing coexisting LTCs in the community, through balancing condition demands and calling for more holistic strategies.	"An internal battle": living with coexisting conditions<br>Seeing me, not just my long-term conditions
Self-managing: a choice or systemic exclusion	Focused on a critical exploration of structural barriers in the healthcare system for adults living with coexisting physical and mental LTCs, and how people responded by creating their own individualised self-management meaning.	"You just feel lost": Navigating the healthcare system<br>"Taking control" through self-management
Unseen struggles: learning to live with my long-term conditions	Centred on how participants felt their lived experiences were undermined, either due to age or the invisibility of LTCs leading to internal struggles regarding their identities.	My medical identity vs my social identity<br>Self-managing now for my future

~~3.3.~~ Theme 1. Moving beyond sink or swim: scaffolding support to self-manage

~~3.3.1.~~ "An internal battle": living with coexisting conditions

All participants described a deeply "interlinking" relationship between their physical and mental health LTCs, whereby one condition intensified the other's symptoms often negatively. This bidirectional relationship made addressing coexisting LTCs simultaneously very challenging, frequently resulting in the prioritisation of one

condition with remaining LTCs forced to take a “backseat”. For many, there was a continual shifting of priorities depending on the LTC causing the most prevalent concerns, repeatedly reported as pain or the external effects of mental health on friends and family. This fluidity contributed to a cycle of fatigue, emotional exhaustion, and frustration.

> *“It’s like pushing a boulder up a hill and if I take my hand off that boulder to focus on my mental health and that boulder starts rolling, it’s just like, uh my God. Now we have to start all of this again” (Rosemary)*

Seven participants described managing their mental and physical LTCs concurrently as an ongoing challenge, referred to as an “internal battle” or “double edged blade”. All participants went on to note unlike their physical LTCs which they felt had clear, more tangible self-management strategies, mental health concerns were harder to address and slower to resolve.

> *“I think, I think I personally prioritise my mental health over my physical health just because as long as I’m not like acutely unwell physically I can get back on top of my physical health but sometimes I know that if my mental health suffers it takes longer to get out of that kind of rut than it would if I was physically ill. Um so I do prioritise my mental health just because I know the consequences can be a bit more severe in terms of it I kind of slip on that end of things.” (Ashley)*

This lack of immediate identification and relief often led to reduced motivation to engage in self-management activities, feeding into a “downward spiral”. Overall, participants conveyed that the interplay between LTCs and constant renegotiation of

self-management strategies ~~played~~ had a significant emotional and physical ~~toll~~ ~~in~~impact on their daily lives.

> *"You kind of just don't want to bother with self-managing after that because you're like what's the point if it's, if it's not worked, what's the point? And you sort of, sort of I don't know how to describe it, it's more, it's like a pit you sort of just get stuck in this pit of what's the point, because nothing works and I just have to deal with it but I don't want to deal with it and so you're just stuck in this like little downward spiral almost." (Laticia)*

~~3.3.2.~~ Seeing me, not just my long-term conditions

Despite the complexity of managing coexisting conditions, participants reported a persistent expectation to adhere to standardised models of care. Grace and Simone described these experiences as dehumanising making them feel like "guinea pigs" in a system overly focused on medications. Further supported by a strong rejection of a "one-size-fits-all" approach with participants instead calling for tailored strategies that acknowledge both the individual and specific interplay of their LTCs.

> *"Instead, quite often I'm faced with you need to do this and you need to do that and I'm like, ok, but we're not really thinking about what does and doesn't work. We're kind of trying to put that one-size-fits-all thing." (Rosemary)*

> *"Yeah, the WRAP [Wellness Recovery Action Plan] planning for mental health, I think that was really good in making me identify what kind of like, asking those questions and putting it back to me to kind of tell. Making me kind of work out what helped, like facilitating me to work out what helped me. They, I think it's a lot of like just communicating to me that they were hopeful*

for me to go and often live this like good life whilst self-managing. I think it was just their overall approach of being very like hopeful and realistic and listening to me about what worked and what didn't work. And like when things weren't going so well just involving me and making the decisions to do the things to tailor my treatment or like make a plan like very much involving me in the planning. Whereas like with the GP, they just made the plan and gave it to me." (Ruby)

Although recognised by all participants, this need was especially pronounced among Poppy, Ruby and Oskar who wanted healthcare professionals to acknowledge self-management "from the get go" of their chronic illness "journeys", as they found receiving diagnoses and treatment planning to be overwhelming moments.

"I think it wasn't really, when I was getting my diagnosis it wasn't really emphasised that how controllable this is. I think it's more something that I've learned with time. Um, whereas like I think some reassurance from the start of hey look, this is like this is a very treatable thing, yeah, like, this is a very manageable problem like it would help with not panicking at the, at the start" (Oskar)

Six participants emphasised the importance of early interventions and education on coexisting LTCs, including timely conversations post-diagnosis which explore personalised self-management strategies, reinforcing motivation and helping to establish individualised goals.

"I think as a healthcare professional acknowledging that is so important, like talking about exercise, talking about hobbies, you know, Maslow's hierarchy

> *of needs what's at the bottom part like what's your purpose kind of thing I think is really important' (Simone)*

A consensus among participants was that feeling heard and collaboration with healthcare providers when planning care are critical factors to long-term engagement. However, to achieve this goal there is a need to move away from the current perceived sink or swim model which places sole responsibility on the individual and their personal resources, to a collaborative approach where support is scaffolded over time.

> *"It has just been a lot of throwing them in the deep end and hope that they can swim. It definitely has been an approach of that and so a lot of the stuff that you figure out, you either figure out on by yourself or using your own resources such as paying for counselling." (Laticia)*

Although when planning care, Poppy stressed the value of practical over theoretical tools. This opinion was supported by Ruby, who together highlighted the importance of usable, realistic approaches tailored to the challenges of managing coexisting LTCs, although they hadn't personally found one yet. However, Simone spoke of her "toolkit", whereby self-management strategies are colour-coded green, orange or red to represent corresponding symptom severity. This approach was designed in collaboration with healthcare providers creating a tangible, effective self-management plan which aided her to "kill two birds with one stone" referring to her coexisting LTCs.

> *"We've made a plan. In fact, we've made a plan, here's our number at the bottom we might not get back to you for eight weeks but you know we've got it. I think that gives someone, that puts them in control of their outcomes and*

their own health and do you know what I mean, in like a more palatable way" (Simone)

~~3.4.~~ Theme 2. Self-managing: a choice or systemic exclusion

~~3.4.1.~~ "You just feel lost": Navigating the healthcare system

Whilst most participants wanted the healthcare system to move towards a "holistic route", their current reality was often opposite. Many participants described damaged relationships with healthcare professionals characterised by feeling dismissed, ignored or disbelieved, which was regularly linked to diagnostic overshadowing.

> *"I just think that when you go with a problem they should consider your whole, your whole story instead of, 'cause I've not only had like, it's not just. If I go there and say I'm, you know, I feel sick or whatever, um I think it's quite easy for them to say, oh, it's probably you're probably anxious or whatever when really, well you should actually look into that, because I've also had surgery. So, you could be missing something else type thing, but, yeah, I don't know, I just think they should consider the story as a whole and not. They should take more time before they come to a conclusion, I think" (Grace)*

Enduring these "constant experiences" contributed towards a resentment of healthcare providers among seven participants. These participants withdrew from formal care and turned towards self-management, not as a preference for LTC management but a necessity born out of advocacy fatigue.

"So, it was kind of a it was a bit of a fight with my GP trying to just get like the testing done and, and push for the diagnosis just because then it did entitle me to like it just allowed me to have more understanding about myself." (Ashley)

Instead of traditional services, informal sources of support such as online communities, social media, and support groups were more popular. These spaces offered validation and understanding, regularly missing in clinical encounters, although Poppy noted the absence of professional healthcare involvement could foster uncertainty about the reliability of shared advice creating self-doubt in one's self-management abilities.

"Um I worry that like I'm possibly not doing the right things or there are things I'm missing, because I'm not having any input from people that really know what they're talking about" (Poppy)

Structural barriers within the healthcare system were considered by all participants. They highlighted poor communication, fragmented care pathways, complex referral processes, and geographical constraints as persistent obstacles to self-managing their coexisting LTCs. There was a clear need to streamline services and integrate care to create healthcare systems which are inclusive and responsive opposed to exclusionary and delayed.

~~3.4.2.~~ "Taking control" through self-management

Despite their common negative care experiences, all participants recounted a gradual shift from service reliance to personal agency in self-managing their LTCs. All participants recounted how the meaning of self-management had evolved into

some form of “taking control”, whereby traditional clinical outcomes were replaced with personal definitions such as achieving a sense of normality, independence, happiness, productivity or simply basic functioning. As a method to achieve these goals, knowledge emerged as a key tool, with participants using a variety of strategies to build their understanding including peer learning, trial and error, formal education, and personal research.

> *“I’ve had a lot of time for trial and error. I’ve had a lot of time to do different things, find what works for me and used like different tools to kind of find what works for me.” (Ruby)*

> *“I think my mum looking into how to properly self-manage and all of that and she like gave me these like cliff notes basically that I could like look into myself because at the time where this information was like when my mum was looking into it, I was in the middle of a gout attack. I wasn’t looking up anything for myself. Yeah, that was actually the most helpful thing anyone’s ever done.” (Oskar)*

However, when gathering this information Rosemary and Ashley encountered difficulties accessing relevant condition specific information, especially when unwell which felt like a “backwards” process. Supported by Oskar and Laticia who recounted knowledge finding as an “energy consuming task” which was at odds with trying to meet their basic daily needs. Therefore, recommendations for a reliable “bank” of information from the healthcare systems were idealised to mitigate this barrier.

> *“I understand that GPs are generalists, but it would be nice if they had, you know, sort of a bank. You know how, like GPs, they’ve always got leaflets or*

information sheets, it would be nice if those kind of existed about my condition that they could pass on instead of me being the one going out and doing all of this research" (Rosemary)

Where this information was already available, through charity websites, Poppy expressed a strong desire for information to be presented from a holistic self-management lens rather than a purely biomedical perspective.

"There's some really good like guidance on Ehlers-Danlos, they have on their own website which has some really helpful stuff. But a lot of it, like when I look into it, it's either really fundamental stuff or it's not written in a perspective of self-management at all. It's written in like these are the biological components of hypermobility. And I'm like, ugh, I don't care" (Poppy)

~~3.5.~~ Theme 3. Unseen struggles: learning to live with my long-term conditions

~~3.5.1.~~ My medical identity vs my social identity

A key barrier to self-management expressed by all participants was how ageism and stigma significantly shaped their experiences of care, with their youth causing their concerns to be invalidated or second-guessed.

"Ugh to be honest I just showed up one day, the woman looked at my foot and she said it looks like gout, but you're young so do you have a family history?" (Oskar)

Many felt age created a hierarchy of legitimacy where youth conflicted with perceptions of illness, leaving them to feel invisible or misunderstood. This tension was especially pronounced for Rosemary and Poppy, who revealed this feeling was further complicated by living with LTCs which lacked objective physical signs. The absence of visible symptoms appeared to undermine their credibility in social and clinical settings, causing them to “doubt” their level of illness and intensified feelings of exclusion.

> *“Um, it just feels like everything that I’ve come up against is kind of you have to do it and you have to be the only person in it when that’s just not the way that I’m able to operate you know, like it’s if a person, I don’t know, needed a crutch or a walking stick, but for some reason the GP wants them to walk through the surgery without a walking stick that just feels a bit irritating.” (Rosemary)*

> *“I just have this thought of, like, we are all just tired and like just go to uni and get a grip, and it’s not that big of a deal and actually it’s not normal and it’s, it’s not something everyone deals with um and trying to remember that because I can be really hard on myself and be like, why can’t I just push through? But actually, because it’s a medical condition, you wouldn’t tell someone with like asthma to push through an asthma attack like.” (Poppy)*

While there was a desire for greater recognition and visibility of younger people with less-represented LTCs, Simone feared being boxed in or stereotyped as a result.

> *“I understand that there needs to be visibility with chronic pain conditions and the more we talk about it, but I did wonder about the stigma and the label of labelling myself as someone like if I presume this identity of someone with*

chronic pain what have I, have I actually put myself into a box where I can't, do you know what I mean? It becomes part of your identity... I tried it and realised actually I've just minimised myself more as a person because now I'm just some you know [18-24]-year-old with chronic pain and not someone who likes going out." (Simone)

These experiences contributed to a blurring of identities: where medical needs became entangled with personal identity, overwhelming their sense of self. However, obtaining formal diagnoses was viewed as both gateways to care and burdensome, concurrently validating experiences whilst constraining identity. Balancing medical and social identities was particularly difficult for those in their early twenties who spoke about feeling torn when managing their health and maintaining friendships, relationships and a sense of normality. This created an internal conflict marked by sacrifice and loss as participants struggled to reconcile youth with chronic illness.

"I didn't want to be that like mentally ill friend that was only talking about mental her mental illness. That friend that was always like kind of dragging people down or like, oh, my mental health is rubbish today." (Ashley)

"I love festivals and I love music and I think when my symptoms got out of control and I was like I actually don't, I don't know if I can do this and this is really upsetting." (Simone)

~~3.5.2.~~ Self-managing now for my future

Following the dilemma of being younger and living with LTCs, participants highlighted significant gaps in self-management resources tailored towards younger people. They found information about self-management to have a primary condition

focus or be “catered” towards older generations, failing to reflect the realities of youth. There was clear frustration at the assumption of younger people being uninterested in self-managing, where in fact participants expressed strong motivation to learn and adopt strategies to maintain their health. Poppy, Oskar and Simone wanted “sustainable” solutions that allowed them to participate in social activities like clubbing, drinking or festivals without forfeiting their mental or physical wellbeing.

> *“I also think like a lot of self-management, I see definitely tends to be catered towards like an older generation. Like, it’s like, oh you want to move more? Go out in the garden or play bowls...but the, the like sustainable lifestyle changes don’t often seem to accommodate for being younger and like your social groups looking different and being at university, or because I think they just assume that people aren’t trying to self-manage in those situations. And it, it makes it really difficult because it, it does like when I look into things, it’s like it just doesn’t feel like it’s catered to me and my life at all.” (Poppy)*

A key theme raised was the need for healthcare providers to recognise varying life stages alter how LTCs impact an individual’s life and therefore their self-management approach. Both Grace and Ruby recalled losing the ability to self-manage in childhood due to parental or institutional involvement. These events shaped their views of self-management in adulthood, with Grace wanting to see self-management as a choice not a discipline, whilst Ruby viewed it as a privilege after it was “taken out of my hands”.

> *“So, it’s almost like I work harder to self-manage because I don’t want to experience that again but then also I feel like, it’s like for me part of it is having the power of being able to self-manage myself like it’s such because its*

> *something that I haven't been able to do before, um or like it's more been taken away from me. Because I wasn't in a place to do that, it's, I almost feel like I've got a bit of like, it's a bit of a privilege to be able to do that myself." (Ruby)*

A further barrier was the emotional and practical toll of self-managing as an emerging adult, having to account for future deterioration and LTC progression. Ruby and Poppy noted this outlook as "quite a hard thing to accept" but were resolved to incorporate self-management as a proactive measure to age well. Whereas Simone determined it was best for her to live in the present "I kind of just do what I want and then reap the consequences".

> *"I try my best to make choices that will help me age well, but there's definitely some fear of like, what's my future going to look life if things, things could get progressively worse. So, I have a component of me that's that it's quite scary and then there's just also bits of me that just get annoyed. I'm like, what, for God's sake [laughs], I don't need to like, I don't need to be thinking about this at [18-24] but like also I do. But you know, one of those." (Poppy)*

Other key transitions of aging such as moving between school, university and work arose as vulnerable periods marked by gaps in support, embarrassment around disclosing health needs and uncertainty requesting reasonable adjustments. Ashley and Laticia discussed the lack of employer knowledge of coinciding conditions, and these were evident areas of improvement needed to foster self-management.

> *"The services that you need to go to in your like workplace aren't as obvious as well. Like I didn't know about the basically what is like the DSA [Disabled Students' Allowance] equivalent of working like and I've already forgotten it's*

> *name, but like access to work, I think it's yeah access to work. I didn't know anything about that. No one told me. It's that, that into that's this not the seamless, what's the word, that sort of movement on from university on from education into like working life. Like no one prepares you for that so you don't know what you have available and even when you go looking, it's not that obvious. Like they leave it quite up to you which don't get me wrong is, is good in some parts but at the same time it's I also don't know because it's my first time in this situation and I don't know what I can ask for." (Laticia)*

Despite these barriers, participants offered solution-focused suggestions such as digital profile packs detailing their LTCs and self-management needs to be sent to educational or occupational supervisors, streamlining advocacy processes and clearer support pathways. Their message was clear: young people are willing and ready to self-manage but they need systems whether that be healthcare, higher education or occupational that are designed to support their self-management goals, not assumptions overlooking them.

~~4.~~ Discussion

~~This qualitative study has explored the facilitators and barriers to self-management for adults living with coexisting physical and mental LTCs. Our findings offer an original contribution to the literature by reframing the view that age, particularly among young adults, is a critical determinant of health and social inequalities within self-management. Previous studies exploring inequalities in multiple LTCs research have predominantly centred on socioeconomic status, education and health literacy as primary determinants of disparity [53-55]. However, our findings suggest that~~

~~young age is an overlooked axis of inequality, with emerging adults experiencing distinct, under-recognised challenges in self-management compared to other age groups. These findings contest dominant narratives found within current self-management frameworks, underscoring the need to reconsider how age-related developmental factors intersect with chronic illness management in early adulthood [56-58].~~

This qualitative study has explored the facilitators and barriers to self-management for adults living with coexisting physical and mental LTCs. Our findings add a new perspective to the literature by evidencing how developmental and life-stage factors may shape experiences of self-management and contribute to health and social inequalities. Previous studies exploring inequalities in multiple LTCs research have predominantly centred on socioeconomic status [58,59], education and health literacy [60-62] as primary determinants of disparity. While these factors remain important, our findings suggest that age and life-experiences may also heavily influence how individuals navigate the complexities of managing coexisting conditions. Although our sample was heavily weighted towards younger adults and women, which may have influenced the analysis and limits the transferability of the results, our findings suggest that young age represents an overlooked axis of inequality with emerging adults experiencing distinct, under-recognised challenges in self-management compared to other age groups. These findings contest dominant narratives found within current self-management frameworks, underscoring the need to reconsider how age-related developmental factors intersect with LTC management [63-65].

~~The findings from our study offer a novel perspective by providing detailed accounts of participants shared and complex yet varied self-management experiences of~~

coexisting physical and mental LTCs. Their experiences revealed a range of developmental and psychosocial challenges associated with self-managing coexisting LTCs during early adulthood. Participants recalled constant negative interactions with healthcare professionals and services often attributed to their age or the complexity of living with coexisting LTCs. Our data showed these experiences detrimentally impacted their trust and view of traditional healthcare services thus prompting a growing reliance on peer networks and social media platforms as alternative sources of self-management support, signalling a notable shift in both the locus and modality of self-management behaviours among younger adults. Furthermore, the analysis indicated pronounced significant health and social care disparities including the absence of tailored guidance for coexisting LTCs and varying life stages; difficulties navigating evolving medical and social identities; and systematic barriers encountered through key transitional phases from education to employment. Collectively, these findings demonstrate the critical role of young age as a structural factor in shaping self-management capacity, which must be integrated into policy and practice to prevent further health and social inequalities.

The findings from our study offer a conceptual leap by providing detailed accounts of participants shared, complex, and varied experiences of self-managing coexisting physical and mental LTCs. Participants recalled constant negative interactions with healthcare professionals and services which they attributed to either their age or the complexity of living with coexisting LTCs. Our data showed these experiences detrimentally impacted their trust and view of traditional healthcare services thus prompting a growing reliance on peer networks and social media platforms as alternative sources of self-management support, signalling a notable shift in both the locus and modality of self-management behaviours. Furthermore, the

analysis indicated pronounced healthcare disparities owing to the visibility or invisibility of their coexisting conditions, which shaped how their care needs were recognised and responded to by others. Moreover, participants highlighted a lack of scaffolded self-management support specifically tailored to coexisting conditions, reflecting broader gaps within both healthcare provision and support resources regarding integrated self-management for concurrent mental and physical LTCs. Collectively, these findings demonstrate the critical reform required in both professional and societal understandings of living with and self-managing coexisting conditions to better support this population to live well. These insights must be integrated into policy and practice to help prevent the further widening of health and social inequalities.

Compared with previous literature, our research both aligns with and diverges from dominant perspectives of self-management [66-68 ~~59-61~~]. Whilst existing literature has addressed aspects of systematic facilitators and barriers to self-management for adults living with coexisting LTCS [27,69,70 ~~22,62,63~~], concurrent with our findings, few papers have explicitly isolated younger age as a central concept to health and social inequalities within the self-management of coexisting LTCs. Despite being routinely collected, age is seldom critically examined beyond the context of aging in self-management. As demonstrated in our results, young adults living with coexisting LTCs often experience stigma both from healthcare professionals and peers when discussing their LTCs, leading to experiences of self-doubt, resentment and feeling both medically and socially excluded. The youth-age dichotomy often constructs these life stages as oppositional, reinforcing assumptions of youth equating health, adaptability and resilience. However, this overlooks the profound self-management challenges faced by younger adults, which whilst different in nature are equally as

significant as those faced by older populations [71,72 64,65]. Although the World Health Organization [8,7366] recognises age as a social determinant of health, national and international guidelines [1,14,74 14,67] remain largely age-neutral, failing to account for the developmental and lifestyle factors which shape self-management experiences across the life course. While older and working-age adults are likely to experience distinct barriers to self-management [75], these challenges are more commonly represented within existing research and policy discourse, whereas the experiences of younger adults remain comparatively underexplored. shaping self-management in younger populations. Recent evidence, including the NHS Fit for the Future Plan [14] demonstrates that LTC prevalence is no longer driven solely by an aging population, with increasing numbers of working-age and young adults affected. Nonetheless, policy responses continue to prioritise older adults, framed through deficit-based narratives of cognitive decline or low health and digital literacy [76,77 68,69]. Whilst emerging initiatives, such as Young Future Hubs, remain limited in scope supporting only those under twenty-five and focusing primarily on mental health and social needs [78 70]. This leaves a significant care gap for emerging adults living with coexisting LTCs who require tailored and integrated support for their physical, mental, and social care needs. Our study challenges the prevailing assumption that younger age implies a lesser need or greater capacity for self-management. Such assumptions marginalise emerging adults living with coexisting LTCs whose health and social care needs may fall outside of normative expectations across health, academic and occupational settings. These findings indicate current care pathways must be adapted to better support young adults with coexisting LTCs through integrated, developmentally appropriate self-management strategies.

~~Our~~ Additionally, our findings further showed participants who lived with non-visible LTCs such as hypermobility spectrum disorder, irritable bowel syndrome, or mental health conditions faced significant barriers to professional recognition and self-management support. Although non-visible chronic illnesses have gained increasing public recognition in recent years, for example through initiatives such as the sunflower lanyard [79~~71~~], individuals with non-visible LTCs continue to experience stigma and challenges to illness legitimacy in both medical and social settings [80~~72~~]. Participants attributed these difficulties to care professionals' prioritisation of visible or objective indicators such as appearance, use of mobility aids, and abnormal diagnostic markers over lived experience and reported impacts on quality of life. This lack of recognition contributed to feelings of exclusion and stigma [12,81 ~~73,74~~] reinforcing barriers to accessing care and wider support, including reasonable adjustments in education and employment [82,83 ~~75,76~~]. Requiring individuals to prove the credibility of their ill health exemplifies epistemic injustice, distorts clinical encounters and undermines the therapeutic relationships essential to effective practice. These obstacles were compounded by self-management interventions narrowly tailored towards physical LTCs with clear biomedical markers and prescribed behaviours such as exercise, diet, and medication management [84,85 ~~77,78~~]. Consequently, this focus marginalises individuals with invisible, fluctuating or complex LTCs, particularly those with coexisting mental health conditions. Moreover, the limited or superficial integration of mental health support was misaligned with participants lived experiences, reducing engagement and intervention effectiveness. Participants reported current interventions failed to accommodate their coexisting LTCs often forcing them to prioritise one over another, despite MLTCs increasingly reflected in routine clinical practice. Without embedding mental health as a core

component of LTC management, rather than an adjunct, adults with coexisting LTCs will continue to experience unmet needs, poorer health outcomes, and inequitable access to support [86-88 ~~79-81~~]. To prevent further widening of health inequalities, self-management interventions must recognise invisible LTCs, incorporate subjective symptoms, and embed mental health as a foundational element.

Our findings illustrated that participants perceived a lack of tailored self-management support~~ relevant to both their age and combinations of LTCs~~. Existing strategies were experienced as biomedical in focus; catered towards older adults; and insufficiently inclusive of different lifestyles. This was evidenced within the literature by a paucity of self-management frameworks targeted towards both emerging adults and those with coexisting physical-mental LTCs. Dominant care models underpinning self-management interventions such as the Chronic Care Model [89~~82~~] and Bandura's self-efficacy theory [90~~83~~], have limited applicability for these groups given their focus on single physical conditions and their presupposition for a level of autonomy, cognitive maturity, and life stability [91~~84~~]. As such, participants described uncertainty about managing their LTCs cohesively without compromising social participation and relationships, which they identified as essential to maintaining mental wellbeing. In contrast, Paterson's Shifting Perspective Model of Chronic Illness [92~~85~~], which conceptualises chronic illness as a dynamic and evolving experience across the life course, offers a more appropriate framework for coexisting LTC self-management. The recognition that individuals move between periods of illness and wellness resonated strongly with participant narratives. Although our findings extend this model by suggesting that illness and wellbeing may coexist simultaneously rather than as alternating states. This reframing underlines the need for sustainable self-management resources, which enable individuals to prioritise

wellbeing and social participation even during periods of illness. Notwithstanding, to avoid exacerbating health and social inequalities, the design of such resources must account for socioeconomic and cultural contexts as these factors have a moderating effect on self-management support [93,94 ~~86,87~~]. Overall, our findings illustrate a conceptual leap for new self-management frameworks which consider managing coexisting LTC as a developmental process evolving alongside identity formation and illness trajectories.

~~Our findings provide an original contribution to this field of research by emphasising the complex interplay between age, identity and system navigation, offering a new conceptual lens to understand the facilitators and barriers to self-management of coexisting physical and mental LTCs. Acknowledging age, especially emerging adulthood, as a primary determinant of inequality in health and social care demands a shift in both conceptual and practical approaches. Young adults with coexisting LTCs are presently expected to self-manage with no established scaffolding. Without creating developmentally attuned, multisectoral responses for younger adults with coexisting LTCs will continue to be underrepresented and underserved, despite the growing prevalence of LTCs in this age group [88-90]. Our results underline the urgent need to address this demographic disparity, moving from age-neutral to age-responsive strategies to prevent adverse health outcomes and improve quality of life among this population.~~

Our findings provide an original contribution to this field of research by emphasising the complex interplay between age, identity and system navigation, offering a new conceptual lens to understand the facilitators and barriers to self-management of coexisting physical and mental LTCs. Rather than acting as a standalone determinant, age appears to interact with broader structural and individual factors,

including identity formation and service organisation, shaping how individuals experience and engage with self-management practices. The accounts of emerging adults suggest that developmental context may be relevant to understand these processes across adulthood. Overall, the results emphasise the importance of developing age-sensitive self-management strategies, improving the visibility of coexisting LTCs within care provision, and ensuring mental health is recognised as a central and integral component of self-management alongside physical health needs.

~~4.1.~~ Implications for practice, policy and research

~~4.1.1.~~ Clinical practice and policy

Our study stresses the need to create age-sensitive self-management frameworks reflecting the distinctive biopsychosocial domains within young adulthood. As traditionally self-management models of care have focused on compliance and structured interventions tailored towards older generations which may be less effective for individuals still forming their identity and autonomy. Therefore, our findings offer a rationale for policymakers and practice providers to co-create a self-management framework which incorporates age-related barriers to engagement and explicitly embeds mental health support as a core component. Incorporating the priories and lived experiences of younger adults into clinical guidelines may enhance sustained engagement in both short and long-term self-management behaviours.

~~4.1.2.~~ Future research

~~Future research is warranted to examine how young adulthood and intersecting sociological factors such as social media use, peer support, and unstructured~~

lifestyles shape self-management intervention uptake. Moreover, quantitative expansion of this work could employ longitudinal surveys to systematically examine how specific facilitators (social support, technology, integrated care) and barriers (lack of tailored guidance, care discontinuity, social stigma) influence how self-management behaviours evolve in emerging adults living with coexisting LTCs at set time-points. Based upon the results, there is potential to codesign targeted self-management interventions for emerging adults, that integrate both medical and social needs, ensuring relevance and effectiveness for this underserved group.

Future research is warranted to examine how young adulthood and intersecting sociological factors such as social media use, peer support, and unstructured lifestyles shape self-management intervention uptake. Moreover, quantitative expansion of this work could employ longitudinal surveys to systematically examine how key facilitators (social support, technology, integrated care) and barriers (lack of tailored guidance, care discontinuity, social stigma) influence the development and evolution of self-management behaviours across the life course in adults living with coexisting physical-mental LTCs. A crucial element of this approach would be ensuring maximum variation in sampling to achieve representation across key demographic variables such as age, gender, socioeconomic status and types of LTC combination alongside differing levels of digital engagement and healthcare access. This would enable more robust comparative analyses across groups and strengthen the validity and transferability of findings. Based upon the results, there is potential to codesign targeted self-management interventions that integrate both medical and social needs, ensuring relevance and effectiveness for this underserved group.

Formatted: Font: (Default) Arial

Formatted: Font: (Default) Arial, English (United Kingdom)

Formatted: Line spacing: Double

~~4.2.~~ Strengths and Limitations

~~A key strength of this study lies in its use of interpretative phenomenological analysis. This methodology prioritises gathering in-depth insight of participants' experiences although the small sample size, whilst consistent with IPA's idiographic focus, may have limited the breadth of experiences captured. Nonetheless, the recurrence of similar themes infers a degree of convergence and robustness. A further strength was the peer debriefing between two authors, strengthening the validation of both the analytical process and emerging themes, supporting the credibility of findings. Finally, the study's recruitment strategy relied on participants' digital skills, which may have unintentionally excluded those from underserved groups or older populations. Additionally, gathering comprehensive demographic data (socioeconomic status, ethnicity, digital skills) could have enabled further analysis of relationships between individual contexts and emergent themes.~~

A key strength of this study lies in its use of Interpretative Phenomenological Analysis which enables in-depth exploration of participants lived experiences. Although the small sample size, consistent with IPA's idiographic focus, may have limited the breadth of experiences captured; the recurrence of similar themes suggests a degree of convergence and robustness. Peer debriefing between two authors further strengthened the analytical process and supported the credibility of the findings. However, there were several limitations within this study to note. Firstly, the recruitment strategy relied heavily on digital access and skills, which may have excluded underserved or older populations. Additionally, the researchers did not formally track which recruitment methods yielded participants, limiting the ability to evaluate the effectiveness of recruitment streams and inform future research. Secondly, the sample size was relatively small and skewed towards

female participants and younger adults, potentially reflecting self-selection bias and limiting transferability of the results. Finally, the absence of socioeconomic, educational, and employment data represents a key limitation, as collecting these demographic factors could have enabled a more nuanced interpretation of the findings.

5. Conclusion

This qualitative study examined the facilitators and barriers to self-management for adults living with physical LTCs and coexisting anxiety/depression. Our findings have offered a unique and unexpected contribution to the field by promoting younger age as a significant inequality in the self-management of coexisting LTCs. By centering on the voices of emerging adults and exploring their self-management experiences, this study lays a foundation for more inclusive, responsive and age-sensitive support to be developed. Addressing this age disparity is crucial: adapting health and social care services to allow for more comprehensive consideration of younger age will prevent a widening generational gap in the treatment and self-management of coexisting LTCs.

This qualitative study examined the facilitators and barriers to self-management for adults living with physical LTCs and coexisting anxiety/depression. Overall, the study contributes to a more nuanced understanding of the lived experience of managing coexisting physical and mental LTCs, emphasising the need for person-cantered, flexible, and age-appropriate responses to self-management support. While our study centred on the voices of emerging adults, the study provides a foundation for further research comparing self-management experiences across different age groups of adults living with coexisting conditions. Such work would support the

development of life-course informed approaches to self-management that better reflect the developmental context and evolving needs of individuals across adulthood, which may inform the design of more tailored and effective self-management strategies.

Acknowledgements

The authors would like to acknowledge and thank the participants and PPIEP members who took the time and effort to take part in this research and share their experiences.

References

[1] United Kingdom. Department of Health and Social Care. Major conditions strategy: case for change and our strategic framework [Internet]. London: Department of Health and Social Care; 2023 [cited 2025 August 21]. Available from: https://www.gov.uk/government/publications/major-conditions-strategy-case-for-change-and-our-strategic-framework/major-conditions-strategy-case-for-change-and-our-strategic-framework--2

[2] NHS England and NHS Improvement. The Improving Access to Psychological Therapies (IAPT) Pathway for People with Long-term Physical Health Conditions and Medically Unexplained Symptoms [Internet]. London: NHS England; 2018 [cited 2025 August 21]. Available from: https://www.england.nhs.uk/wp-content/uploads/2018/03/improving-access-to-psychological-therapies-long-term-conditions-pathway.pdf

[3] World Health Organization. Mental health and COVID-19: early evidence of the pandemic's impact: scientific brief, 2nd March 2022 [Internet]. Geneva: World Health Organization; 2022 [cited 2025 August 15]. Available from: https://iris.who.int/bitstream/handle/10665/352189/WHO-2019-nCoV-Sci-Brief-Mental-health-2022.1-eng.pdf?sequence=1

[4] Pearson-Stuttard J, Ezzati M, Gregg EW. Multimorbidity – a defining challenge for health systems. The Lancet Public Health [Internet]. 2019 December [cited 2025 August 12]; 4(12): Article number e599-3600. Available from: https://www.thelancet.com/journals/lanpub/article/PIIS2468-2667(19)30222-1/fulltext

[5] Naylor C, Das P, Ross S, Honeyman M, Thompson J, Gilburt H. Bringing together physical and mental health: a new frontier for integrated care [Internet]. London: The King's Fund; 2016 [cited 2025 August 20]. Available from: https://assets.kingsfund.org.uk/f/256914/x/5fa0a562d8/bringing_together_physical_and_mental_health_march_2016.pdf

[6] Mental Health Foundation. Mental health problems cost UK economy at least GBP 188 billion a year – new research [Internet]. London: Mental Health Foundation; 2022 March 2 [cited 2025 August 18]. Available from: https://www.mentalhealth.org.uk/about-us/news/mental-health-problems-cost-uk-economy-least-gbp-118-billion-year-new-research

[7] World Health Organization. World health statistics 2018: monitoring health for the SDGs, sustainable development goals [Internet]. Geneva: World Health Organization; 2018 [cited 2025 August 17]. Available from:

https://www.who.int/docs/default-source/gho-documents/world-health-statistic-reports/6-june-18108-world-health-statistics-2018.pdf

[8] World Health Organization. World report on social determinants of health equity [Internet]. Geneva: World Health Organization; 2025 May 6 [cited 2025 August 17]. Available from: https://www.who.int/publications/i/item/9789240107588

[9] Holland E, Matthews K, Macdonald S, Ashworth M, Laidlaw L, Cheung KSY, et al. The impact of living with multiple long-term conditions (multimorbidity) on everyday life – a qualitative evidence synthesis. BMC Public Health [Internet]. 2024 December 18 [cited 2025 September 1]; 24: Article number 3446. Available from: https://bmcpublichealth.biomedcentral.com/articles/10.1186/s12889-024-20763-8#citeas

[10] Porter T, Ong BN, Sanders T. Living with multimorbidity? The lived experience of multiple chronic conditions in later life. Health (London) [Internet] 2020 November [cited 2025 August 18]; 24(6): 701-718. Available from: https://pubmed.ncbi.nlm.nih.gov/30895825/

[11] Carswell C, Brown JVE, Lister J, Ajjan RA, Anderson SL, Balogun-Katung A, et al. The lived experience of severe mental illness and long-term conditions: a qualitative exploration of service user, carer and health professional perspectives on self-managing co-existing mental and physical conditions. BMC Psychiatry [Internet]. 2022 July 19 [cited 2025 August 17]; 22: Article number 479. Available from: https://doi.org/10.1186/s12888-022-04117-5

[12] Shefer G, Henderson, C, Howard LM, Murray J, Thornicroft G. Diagnostic overshadowing and other challenges involved in the diagnostic process of patients with mental illness who present in emergency departments with physical symptoms –

a qualitative study. PLos One [Internet]. 2015 November 4th [cited 2025 August 13]; 9(11): Article number 111682. Available from: https://doi.org/10.1371/journal.pone.0111682

[13] National Health Service. The NHS Long Term Plan [Internet]. London: National Health Service; 2019 January [cited 2025 November 11]. Available from: https://webarchive.nationalarchives.gov.uk/ukgwa/20230418155402/https:/www.longtermplan.nhs.uk/publication/nhs-long-term-plan/

[14] Department of Health and Social Care. Fit for the future: 10 Year Health Plan for England [Internet]. London: Department of Health and Social Care; 2025 July 3rd [cited 2025 August 13]. Available from: https://assets.publishing.service.gov.uk/media/6888a0b1a11f859994409147/fit-for-the-future-10-year-health-plan-for-england.pdf

[15] van de Velde D, de Zutter F, Satink T, Costa U, Janquart S, Senn D, de Vriendt P. Delineating the concept of self-management in chronic conditions: a concept analysis. BMJ Open [Internet]. 2018 [cited 2025 August 14]; 9(7): Article number e027775. Available from: https://doi.org/10.1136/bmjopen-2018-027775

[16] National Institute for Health and Care Research. Multiple long-term conditions (multimorbidity) and inequality – addressing the challenge: insights from research [Internet]. NIHR Evidence; 2023 September 20 [cited 2025 August 13]. Available from: https://doi.org/10.3310/nihrevidence_59977

[17] Spiers G, Boulton E, Corner L, Craig D, Parker S, Todd, C, Hanratty B. What matters to people with multiple long-term conditions and their carers? Postgraduate Medical Journal [Internet]. 2021 December 17 [cited 2025 August 18]; 99(1169): 159-165. Available from: https://doi.org/10.1136/postgradmedj-2021-140825

[18] Woodward A, Davies N, Walters K, Nimmons D, Stevenson F, Protheroe J, et al. Self-management of multiple long-term conditions: A systematic review of the barriers and facilitators amongst people experiencing socioeconomic deprivation. PLoS One [Internet]. 2023 February 21 [cited 2025 August 17]; 18(2): Article number e0282036. Available from: https://doi.org/10.1371/journal.pone.0282036

[19] World Health Organization. Mental health [Internet]. Geneva: World Health Organization; 2025 October [cited 2026 May 19]. Available from: https://www.who.int/news-room/fact-sheets/detail/mental-health-strengthening-our-response

[20] National Health Service. Low mood, sadness and depression [Internet]. London: National Health Service; 2022 January [cited 2026 May 19]. Available from: https://www.nhs.uk/mental-health/feelings-symptoms-behaviours/feelings-and-symptoms/low-mood-sadness-depression/

[21] National Health Service. Generalised anxiety disorder (GAD) [Internet]. London: National Health Service; 2024 October [cited 2026 May 19]. Available from: https://www.nhs.uk/mental-health/conditions/generalised-anxiety-disorder-gad/

[22] Mental Health Foundation. Self-management of mental ill-health [Internet]. London: Mental Health Foundation; 2022 February [cited 2026 May 19]. Available from: https://www.mentalhealth.org.uk/explore-mental-health/a-z-topics/self-management-mental-ill-health#paragraph-56807

[23] National Institute for Health and Care Excellence. Mental health guidelines [Internet]. London: NICE; 2026 [cited 2026 May 19]. Available from: https://www.nice.org.uk/guidance/indevelopment/gid-hub10005

[24~~19~~] Smith SM, Wallace E, Clyne B, Boland F, Fortin M. Interventions for improving outcomes in patients with multimorbidity in primary care and community setting: a systematic review. Systematic Reviews [Internet}. 2021 October 20 [cited 2025 August 20]; 10: Article number 271. Available from: https://doi.org/10.1186/s13643-021-01817-z

[25~~20~~] Breckner A, Roth C, Glassen K, Wensing M. Self-management perspectives of elderly patients with multimorbidity and practitioners - status, challenges and further support needed? BMC Primary Care [Internet] 2021 November 26 [cited 2025 August 20]; 22: Article number 238. Available from: https://doi.org/10.1186/s12875-021-01584-9

[26~~21~~] Smith L, Simpson G, Holt S, Dambha-Miller H. Digital applications to support self-management of multimorbidity: A scoping review. International Journal of Medical Informatics [Internet]. 2025 October [cited 2025 August 20]; 202: Article number 105988. Available from: https://doi.org/10.1016/j.ijmedinf.2025.105988

[27~~22~~] Balogun-Katung A, Carswell C, Brown JVE, Coventry P, Ajjan R, Alderson A. Exploring the facilitators, barriers and strategies for self-management in adults living with severe mental illness, with and without long-term conditions: A qualitative evidence synthesis. PLOS One [Internet]. 2021 October 26 [cited 2025 September 5]; 16(10): Article number e0258937. Available from: https://doi.org/10.1371/journal.pone.0258937

[28~~23~~] Coventry PA, Young B, Balogun-Katung A, Taylor J, Brown JVE, Kitchen C. Determinants of physical health self-management behaviours in adults with serious mental illness: A systematic review. Frontiers in Psychiatry [Internet]. 2021 August

18 [cited 2025 September 5]; 12: Article number 723962. Available from: https://doi.org/10.3389/fpsyt.2021.723962

[2924] Beaudin J, Chouinard MC, Hudon E, Hudon C. Factors and strategies influencing integrated self-management support for people with chronic diseases and common mental disorders: A qualitative study of Canadian primary care nurses' experiences. Journal of Advanced Nursing Qualitative [Internet]. 2025 March 10 [cited 025 September 5]; 0: 1-16. Available from: https://doi.org/10.1111/jan.16892

[3025] Kappelin C, Sandlund C, Westman J, Wachtler C. Dancing with the patient: a qualitative study of general practitioners' experiences of managing patients with multimorbidity and common mental health problems. BMC Primary Care [Internet]. 2023 April 20 [cited 2025 September 5]; 24: Article number 104. Available from: https://doi.org/10.1186/s12875-023-02056-y

[3126] MIND. The Big Mental Health Report. London: MIND. 2025 [cited 2025 December 8]. Available from: https://www.mind.org.uk/media/zianu2rn/mind_the-big-mental-health-report_digital4.pdf

[3227] United Nations. Transforming our world: the 2030 agenda for sustainable development. New York: United Nations. 2015 [cited 2025 December 8]. Available from: https://sdgs.un.org/2030agenda

[3328] World Health Organization and Calouste Gulbenkian Foundation. Policy options on mental health: a WHO-Gulbenkian Mental Health Platform collaboration. Geneva: World Health Organization. 2017 [cited 2025 December 8]. Available from: https://iris.who.int/server/api/core/bitstreams/9d896b3e-78f8-48f5-bcbd-bb2bc8f77c75/content

[3429] Morgan HM, Entwistle VA, Cribb A, Christmas S, Owens J, Skea ZC, Watt IS. We need to talk about purpose: a critical interpretative synthesis of health and social care professionals' approaches to self-management support for people with long-term conditions. Health Expectations [Internet] 2016 April 14 [cited 2025 August 12]; 20(2): 243-259. Available from: https://doi.org/10.1111/hex.12453

[3530] National Institute for Health and Care Research. Multiple long-term conditions (multimorbidity): making sense of the evidence [Internet]. NIHR Evidence. 2021 March 30 [cited 2025 August 13]. Available from: https://doi.org/10.3310/collection_45881

[3631] NHS England. Improving the physical health of people living with severe mental illness [Internet]. London: NHS; 2024 January 10 [updated 2025 May 7th; cited 2025 August 12]. Available from: https://www.england.nhs.uk/long-read/improving-the-physical-health-of-people-living-with-severe-mental-illness/

[3732] Smith JA, Flower P, Larkin M. Interpretative Phenomenological Analysis: Theory, Method and Research. London: SAGE Publications; 2009.

[3833] Smith JA, Flower P, Larkin M. Interpretative Phenomenological Analysis: Theory, Method and Research. 2nd edition. London: SAGE Publications; 2022.

[3934] Creswell JW, Poth CN. Qualitative Inquiry and Research Design: Choosing Among Five Approaches. 5th edition. Los Angeles: SAGE Publications; 2024

[4035] Pope C, Mays N. Qualitative Research in Health Care. 4th edition. Chichester: John Wiley and Sons Ltd; 2019.

[41~~36~~] Ahmed SK. The pillars of trustworthiness in qualitative research. *Journal of Medicine, Surgery, and Public Health [Internet]* 2024 April [cited 2025 November 11]; 2(1). Available from: https://doi.org/10.1016/j.glmedi.2024.100051

[42~~37~~] Lincoln SY, Guba EG. Naturalistic inquiry. Thousand Oaks, California: Sage; 1985

[43~~38~~] Tong A, Sainsbury P, Craig J. Consolidated criteria for reporting qualitative research (COREQ): a 32-item checklist for interviews and focus groups. International Journal for Quality in Health Care [Internet]. 2007 September 14 [cited 2025 August 13]; 19(6): 349-357. Available from: https://doi.org/10.1093/intqhc/mzm042

[44~~39~~] Green J, Thorogood N, Melendez-Torres GJ. Qualitative Methods for Health Research. 5th edition. Los Angeles: SAGE Publications; 2025.

[45~~40~~] Peat G, Rodriguez A, Smith J. Interpretative phenomenological analysis applied to healthcare research. Evidence Based Nursing [Internet] 2019 January [cited 2025 November 11]; 22(1): 7-9. Available from: https://doi.org/10.1136/ebnurs-2018-103017

[46~~41~~] Malterud K, Siersma V, Guassora AD. Information power: sample content and size in qualitative studies. In: Camid PM, editor. Qualitative research in psychology: Expanding perspectives in methodology and design. 2nd edition. Washington: American Psychological Association, 2021. 67-81 p.

[47~~42~~] Saunders B, Sim J, Kingstone T, Baker S, Waterfield J, Bartlam B, et al. Saturation in qualitative research: exploring its conceptualization and operationalization. Quality and Quantity [Internet] 2017 September 14 [cited 2025 November 7]; 52(4): 1893-1907. Available from: https://doi.org/10.1007/s11135-017-0574-8

[48~~43~~] UK Public General Acts. Data Protection Act. 2018 [cited 2025 August 13]. Available from: https://www.legislation.gov.uk/ukpga/2018/12/contents/enacted

[49~~44~~] Staniszewska S, Brett J, Simera L, Seers K, Mockford C, Goodlad S, et al. GRIPP2 reporting checklists: tools to improve reporting of patient and public involvement in research. British Medical Journal [Internet]. 2017 April 2nd [cited 2025 August 17]; 358 Article number 3453. Available from: https://doi.org/10.1136/bmj.j3453

[50~~45~~] Allen RES, Wiles JL. A rose by any other name: participants choosing research pseudonyms. Qualitative Research in Psychology [Internet]

[51~~46~~] Charlick S, Pincombe J, McKellar L, Fielder A. Making Sense of Participant Experiences: Interpretative Phenomenological Analysis in Midwifery Research. International Journal of Doctoral Studies [Internet]. 2016 January [cited 2025 November 17]; 11: 205-216. Available from: https://doi.org/10.28945/3486

[52~~47~~] Shinebourne P. The theoretical underpinnings of Interpretative Phenomenological Analysis (IPA). Existential Analysis [Internet]. 2011 [cited 2025 November 17]: 22(1): 16-31. Available from: https://psycnet.apa.org/record/2011-05339-003

[53~~48~~] Willig C, Rogers WS, Eatough V, Smith JA. Interpretative Phenomenological Analysis. In: Willig C, Rogers WS, editors. The SAGE Handbook of Qualitative Research in Psychology. 2nd edition. Los Angeles: SAGE Publications Ltd; 2017 [cited 2025 November 17]. p. 193-211. Available from: https://doi.org/10.4135/9781526405555.n12

[54~~49~~] Lloyd N, Hyett N, Kenny A. To Member Check or not to Member Check? An Evaluation of Member Checking in an Interpretative Descriptive Study. International

Journal of Qualitative Methods [Internet]. 2024 November 15 [cited 2025 August 12]; 23. Available from: https://doi.org/10.1177/160940692413013

[55~~50~~] Smith, J. A. (2015) *Qualitative Psychology: A practical guide to research methods*. 3rd edition. London: SAGE Publications.

[56~~51~~] World Medical Association [WMA]. WMA Declaration of Helsinki–Ethical Principles for Medical Research Involving Human Subjects. 2013 [cited 24 February 2026]. Available: https://www.wma.net/policies-post/wma-declaration-of-helsinki-ethical-principles-for-medical-research-involving-human-subjects/

[57~~52~~] Smith JA, Pietkiewicz, I. A practical guide to using Interpretative Phenomenological Analysis in qualitative research psychology. Psychological Journal [Internet]. 2014 [cited 2025 August 12]; 20(1): 7-14. Available from: https://doi.org/10.14691/CPPJ.20.1.7

[58] Woodward A, Nimmons D, Davies, N, Walters K, Stevenson FA, Protheroe J, et al. A qualitative exploration of the barriers and facilitators to self-managing multiple long-term conditions amongst people experiencing socioeconomic deprivation. Health Expectations [Internet]. 2024 April 16 [cited 2025 August 13]; 27(2) Article number 14046. Available from: https://doi.org/10.1111/hex.14046

[59] Wyke E, Baxter ER, Chew-Graham CA, Woodward A, Walters K, Davies N, et al. Managing multiple long-term conditions in people experiencing socioeconomic deprivation: a qualitative study of primary care perspectives. British Journal of General Practice [Internet]. 2026 May [cited 2026 June 1]; 76(766): 366-375. Available from: https://doi.org/10.3399/BJGP.2025.0361

[60] Coventry PA, Fisher L, Kenning C, Bee P, Bower P. Capacity, responsibility, and motivation: a critical qualitative evaluation of patient and practitioner views about

Formatted: Font: (Default) Arial, No underline

barriers to self-managment in people with multimorbidity. BMC Health Services Research [Internet]. 2014 October 14 [cited 2026 June 1]; 14 Article number 536. Available from: https://doi.org/10.1186/s12913-014-0536-y

[6153] Gobeil-Lavoie A-P, Chouinard M-C, Danish A, Hudon C. Characteristics of self-management among patients with complex health needs: a thematic analysis review. BMC Open [Internet]. 2019 [cited 2025 August 14]; 9(5) Article number 028344. Available from: https://doi.org/10.1136/bmjopen-2018-028344

[6254] Joseph L, Krishnan A, Lekha TR, Sasidharan N, Thulaseedhara JV, Valamparampil MJ, et al. Experiences and challenges of people living with multiple long-term conditions in managing their care in primary care settings in Kerala, India: A qualitative study. PLoS One [Internet]. 2024 June 13 [cited 2025 August 13]; 19(6) Article number 0305430. Available from: https://doi.org/10.1371/journal.pone.0305430

~~[55] Woodward A, Nimmons D, Davies, N, Walters K, Stevenson FA, Protheroe J, et al. A qualitative exploration of the barriers and facilitators to self-managing multiple long-term conditions amongst people experiencing socioeconomic deprivation. Health Expectations [Internet]. 2024 April 16 [cited 2025 August 13]; 27(2) Article number 14046. Available from: https://doi.org/10.1111/hex.14046~~

[6356] Mooney O, Lambert V, Gallagher P. Self-management of Long-Term Physical Conditions During Emerging Adulthood: A Systematic Review. Emerging Adulthood [Internet]. 2023 September 29 [cited 2025 September 10]; 11(6): 1446-1468. Available from: https://doi.org/10.1177/21676968231203284

[6457] Corp N, Bray L, Chew-Graham CA, Polidano K, Fisher T, Garmer AD, et al. Self-directed self-management interventions to prevent or address distress in young

Formatted: Font: (Default) Arial

Formatted: Font: (Default) Arial, 12 pt, Font color: Auto

people with long-term physical conditions: A rapid review. Health Expectations [Internet]. 2023 August 2 [cited 2025 September 10]; 26(6): 2164-2190. Available from: https://doi.org/10.1111/hex.13845

[65~~58~~] Wilson C, Stock J. The impact of living with long-term conditions in young adulthood on mental health and identity: What can help? Health Expectations [Internet]. 2019 July 25 [cited 2025 September 10]; 22(5): 1111-1121. Available from: https://doi.org/10.1111/hex.12944

[66~~59~~] Franklin M, Willis K, Lewis S, Smith L. Chronic condition self-management is a social practice. Journal of Sociology [Internet]. 2021 August 26 [cited 2025 September 10]; 59(1): 215-231. Available from: https://doi.org/10.1177/14407833211038059

[67~~60~~] Rijken M, Hujala A, van Ginneken E, Melchiorre MG, Groenewegen P, Schelevis F. Managing multimorbidity: Profiles of integrated care approaches targeting people with multiple chronic conditions in Europe. Health Policy [Internet]. 2018 January [cited 2025 September 10]; 122(1): 44-52. Available from: https://doi.org/10.1016/j.healthpol.2017.10.002

[68~~61~~] Whitty CJM, MacEwen C, Goddard A, Alderson D, Marshall M, Calderwood C, et al. Rising to the challenge of multimorbidity. BMJ [Internet]. 2020 January 6 [cited 2025 September 10]; 368: Article Number l6964. Available from: https://doi.org/10.1136/bmj.l6964

[69~~62~~] Carswell C, Coventry PA, Brown JVE, Alderson SL, Double K, Gilbody S, et al. Development of a Supported Self-management Intervention for People With Severe Mental Illness and Type 2 Diabetes: Theory and Evidence-Based Co-design

Approach. 2023 [cited 2025 August 15]; 12(25) Article number 43597. Available from: https://doi.org/10.2196/43597

[7063] Reilly S, Olier I, Planner C, Doran T, Reeves D, Ashcroft DM, Gask L, Kontopantelis E. Inequalities in physical comorbidity: a longitudinal comparative cohort study of people with severe mental illness in the UK. BMJ Open [Internet]. 2015 December 15 [cited 2025 August 13]; 15(5) Article number 3009010. Available from: https://doi.org/10.1136/bmjopen-2015-009010

[7164] Sattoe JNT, van Staa A, Hilberink SA. Self-management of Young People with Chronic Conditions: A strength-based approach for empowerment and support. Switzerland: Springer Nature, 2021.

[7265] Frantz J, Schoop L, Rhoda A. Self-management in Chronic Illness: Principles, Practice and Empowerment Strategies for Better Health. Switzerland: Springer Nature, 2021.

[7366] World Health Organization. Global report on ageism [Internet]. Geneva: World Health Organization; 2021 [cited 2025 September 10]. Available from: https://iris.who.int/bitstream/handle/10665/340208/9789240016866-eng.pdf?sequence=1

[7467] National Institute for Health and Care Excellence. Multimorbidity: clinical assessment and management [Internet]. London: NICE; 2016 [cited 2025 August 14]. [NG56]. Available from: https://www.nice.org.uk/guidance/ng56

[75] Moody L, Wood E, Needham A, Booth A, Jimenez-Aranda A, Tindale W. Identifying individual enablers and barriers to the use of digital technology for the self-management of long-term conditions by older adults. Journal of Medical

Engineering & Technology [Internet] 2022 June 24 [cited 2026 May 19]; 46(6): 448-461. Available from: https://doi.org/10.1080/03091902.2022.2089249

[7668] National Institute for Health and Care Excellence. Older people with social care needs and multiple long-term conditions [Internet]. London: NICE; 2015 [cited 2025 August 14]. [NG22]. Available from: https://www.nice.org.uk/guidance/ng22

[7769] Nguyen TNM, Whitehead L, Saunders R, Dermody G. Systematic review of perception of barriers and facilitators to chronic disease self-management among older adults: Implications for evidence-based practice. Worldviews on Evidence-Based Nursing [Internet] 2022 January 15 [cited 2025 August 15]; 19(3): 191-200. Available from: https://doi.org/10.1111/wvn.12563

[7870] National Health Service. A blueprint for Young Future hubs [Internet]. London: NHS; 2025 March [cited 2025 November 27]. Available from: https://www.youthaccess.org.uk/sites/default/files/documents/A%20blueprint%20for%20Young%20Futures%20hubs%20by%20Youth%20Access%20and%20partners.pdf

[7971] Hidden Disabilities Sunflower Scheme Limited. About the Sunflower [webpage on the internet]. [cited 2025 December 6] Available from: https://hdsunflower.com/about-hidden-disabilities-sunflower

[8072] Ahtoy P. Behind the hidden struggles linked to invisible disabilities. Understanding their impact to increase mental health awareness for enhanced professional care. Ethics, Medicine and Public Health [Internet]. 2024 [cited 2025 December 8]; 32: Article number 101016. Available from: https://doi.org/10.1016/j.jemep.2024.101016

[81~~73~~] Clement S, Schauman O, Graham T, Maggioni F, Evans-Lacko S, Bexborodovs N, et al. What is the impact of mental health-related stigma on help-seeking? A systematic review of quantitative and qualitative studies. Psychological Medicine [Internet]. 2015 [cited 2025 December 8]; 45(1):11-27. Available from: https://doi.org/10.1017/S0033291714000129

~~[74] Shefer G, Henderson C, Howard LM, Murray J, Thornicroft G. Diagnostic Overshadowing and Other Challenges Involved in the Diagnostic Process of Patients with Mental Illness Who Present in Emergency Departments with Physical Symptoms – A Qualitative Study. PLOS One [Internet]. 2014 November 4 [cited 2025 December 8]; 9(11): Article number e111682. Available from: https://doi.org/10.1371/journal.pone.0111682~~

[82~~75~~] Thomas V, Ahmed H, Lindsay S. Unravelling the Complexities of Workplace Disclosure Among Persons with Non‐Visible Disabilities and Illnesses: A Qualitative Meta‐Ethnography. Journal of Occupational Rehabilitation [Internet]. 2022 [cited 2025 December 8]; 32(1): 538-563. Available from: https://doi.org/10.1007/s10926-022-10023-0

[83~~76~~] Moriña A. When what is unseen does not exist: disclosure, barriers and supports for students with invisible disabilities in higher education. Disability & Society [Internet]. 2024 [cited 2025 December 8]; 39(4): 914-932. Available from: https://doi.org/10.1080/09687599.2022.2113038

[84~~77~~] Morgan HM, Entwistle VA, Cribb A, Christmas S, Owens J, Skea ZC, et al. We need to talk about purpose: a critical interpretive synthesis of health and social care professionals' approaches to self-management support for people with long-

term conditions. Health Expectations [Internet] 2016 April 14 [cited 2025 December 8]; 20(2): 243-259. Available from: https://doi.org/10.1111/hex.12453

[85~~78~~] Hajat C, Stein E. The global burden of multiple chronic conditions: A narrative review. Preventative Medicine Reports [Internet]. 2018 [cited 2025 December 8]; 12(1): 284-293. Available from: https://doi.org/10.1016/j.pmedr.2018.10.008

[86~~79~~] Mitchell AJ, Hardy S, Shiers D. Parity of esteem: addressing the inequalities between mental and physical healthcare. BJPsych Advances [Internet]. 2018 January 2 [cited 2025 December 8]; 23(3): 196-205. Available from: https://doi.org/10.1192/apt.bp.114.014266

[87~~80~~] Department of Health and Social Care. Premature mortality in adults with severe mental illness (SMI). London: GOV.UK; 2018 April 19 [cited 2025 December 8]. Available from: https://www.gov.uk/government/publications/premature-mortality-in-adults-with-severe-mental-illness/premature-mortality-in-adults-with-severe-mental-illness-smi

[88~~81~~] Lowther-Payne HJ, Ushakova A, Beckwith A, Liberty C, Edge R, Lobban F. Understanding inequalities in access to adult mental health services in the UK: a systematic mapping review. BMC Health Services Research [Internet]. 2023 [cited 2025 December 8]; 23(1): Article number 1042. Available from: https://doi.org/10.1186/s12913-023-10030-8

[89~~82~~] Wagner EH. Chronic disease management: what will it take to improve care for chronic illness? Effective Clinical Practice [Internet] 1998 September [cited 2025 August 17]; 1(1): 2-4. Available from: https://pubmed.ncbi.nlm.nih.gov/10345255/

[90~~83~~] Bandura A. Self-efficacy: Toward a unifying theory of behavioral change. Advances in Behaviour Research and Therapy [Internet]. 1978 [cited 2025 August 17]; 1(4): 139-161. Available from: https://doi.org/10.1016/0146-6402(78)90002-4

[91~~84~~] Schoemaker CG, van Geelen SM, Allewijn M, Fernhout M, van Vliet R, Wulffraat N. Self-management support for young people living with fluctuating chronic diseases. Archives of Disease in Childhood [Internet]. 2022 January 10 [cited 2025 August 17]; 107(7): 658-659. Available from: https://doi.org/10.1136/archdischild-2021-323118

[92~~85~~] Paterson BL. The Shifting Perspectives Model of Chronic Illness. Journal of Nursing Scholarship [Internet] 2004 April 23 [cited 2025 August 17]; 33(1): 21-26. Available from: https://doi.org/10.1111/j.1547-5069.2001.00021.x

[93~~86~~] Hardman R, Begg S, Spelten E. What impact do chronic disease self-management support interventions have on health inequity gaps related to socioeconomic status: a systematic review. BMC Health Services Research [Internet] 2020 February 27 [cited 2025 August 14]; 20: Article number 150. Available from: https://bmchealthservres.biomedcentral.com/articles/10.1186/s12913-020-5010-4

[94~~87~~] Schiøtz ML, Stockmarr A, Høst D, Glümer C, Frølich A. Social disparities in the prevalence of multimorbidity. A register-based population study. BMC Public Health [Internet] 2017 May 10 [cited 2025 August 17]; 17(1): 422. Available from: https://doi.org/10.1186/s12889-017-4314-8

~~[88] Smith MD, Wesselbaum D. Well-Being Inequality Among Adolescents and Young Adults. Journal of Adolescent Health [Internet]. 2024 April [cited 2025 August~~

15]; 74(4): 703-712. Available from: https://www.jahonline.org/article/S1054-139X(23)00547-5/fulltext

[89] Shah R, Hagell A, Cheung R. International comparisons of health and wellbeing in adolescence and early adulthood. [Internet] London: Nuffield Trust and Association for Young People's Health; 2019 [cited 2025 August 17]. Available from: https://www.nuffieldtrust.org.uk/sites/default/files/2019-02/1550657729_nt-ayph-adolescent-health-report-web.pdf

[93] Watt T, Raymond A, Rachet-Jacquet L, Head A, Kypridemos C, Kelly E, et al. Health in 2040: projected patterns of illness in England. The Health Foundation [Internet]. 2023 July [cited 2025 November 24]. Available from: https://www.health.org.uk/reports-and-analysis/reports/health-in-2040-projected-patterns-of-illness-in-england

Supporting Information

S1 Checklist. Consolidated Criteria for reporting qualitative studies (COREQ): 32-item checklist.

S2 Checklist. GRIPP2-SF Reporting Checklist.

S1 Fig. Ethical approval.

S2 Fig. Ethical approval.

S1 Table. Interview topic guide.

S2 Table. Stages of Interpretative Phenomenological analysis (extracts from Rosemary's transcript).

S3 Table. Participant contribution to Group Experiential Themes (GETs)

Dear Editor-in-Chief,

Thank you for giving us the opportunity to submit a revised draft of our manuscript titled '"I want to take the power back": An interpretative phenomenological analysis of self-management in coexisting physical and mental long-term condition' to PLOS One. We appreciate the time and effort that the editors and the reviewers have dedicated to providing valuable feedback on the manuscript. We are grateful to the reviewers for their insightful comments on the paper. We have been able to incorporate changes to reflect most of the suggestions provided and these changes are highlighted within the manuscript.

Here is a point-by-point response to the journal, academic editor and reviewers' comments and concerns.

Journal Requirements

Comment 1: Please ensure that your manuscript meets PLOS ONE's style requirements, including those for file naming.

Response: We have carefully reviewed the manuscript and revised the submission to ensure full compliance with PLOS One style requirements, including formatting and file naming conventions. All files have been checked and updated accordingly.

Comment 2: Thank you for uploading your study's underlying data set. Unfortunately, the repository you have noted in your Data Availability statement does not qualify as an acceptable data repository according to PLOS's standards. At this time, please upload the minimal data set necessary to replicate your study's findings to a stable, public repository (such as figshare or Dryad) and provide us with the relevant URLs, DOIs, or accession numbers that may be used to access these data.

Response: We thank the journal for this important comment. In line with institutional requirements and approved ethical procedures relating to participant confidentiality and data governance, the dataset is securely held within the University of Southampton's institutional repository, PURE. An updated Data Availability Statement has now been completed to clarify the location of the data, and a letter from the University of Southampton Data Management Team has been attached detailing institutional procedures.

Comment 3: Please amend either the abstract on the online submission form (via Edit Submission) or the abstract in the manuscript so that they are identical.

Response: We thank the journal for this comment. We have carefully reviewed and amended the abstract in both the manuscript and online submission system to ensure that both versions are now identical.

Comment 4: We note that there is identifying data in the Supporting Information file <S3 Table. Participant contribution to GETs>. Due to the inclusion of these potentially identifying data, we have removed this file from your file inventory. Prior to sharing human research participant data, authors should consult with an ethics committee to ensure data are shared in accordance with participant consent and all applicable local laws.

Response: We thank the journal for this comment. The names included in 'S3 Table. Participant contribution to GETs' were participant pseudonyms rather than identifiable personal information, as stated within the manuscript. To further support clarity regarding confidentiality procedures, we have added a statement to the S3 Table explicitly confirming that all names are pseudonyms used to protect participant anonymity. The revised S3 Table has been updated and reuploaded accordingly.

Comments from Editor

Comment 1: Funding statement – please add funding/grant number.

Response: We thank the Editor for this comment. We have updated the Funding Statement to include the relevant funding source and grant number. The grant: NIHR304046 – Inspiring Students into Research (R1 2023) was awarded to the lead researcher (CL) which covered tuition fees and a stipend. This funding was not allocated to the specific research study.

Comment 2: Sample size is relatively small and heavily skewed (Male 1, female 8). This should be factored in the discussion and perhaps presented as part of study weakness.

Response: We thank the Editor for highlighting this issue. We agree that the sample size and gender imbalance are limitations of the study. This has now been acknowledged in the revised discussion section (Page 29, Lines 652-654) and included explicitly as a study limitation (Page 36, Lines 819-820, 828-830).

Comment 3: Socioeconomic, education/employment status are missing. These are critical determinants of self-management and inequality. Please attribute this to study weakness

Response: We thank the Editor for this important observation. We agree that socioeconomic status, education and employment are key determinants of self-management and health inequality. These variables were not collected in this study, and this limitation has now been explicitly stated as a study weakness (Page 36, Lines 830-833).

Comment 4: It is unclear how interpretations moved from codes to themes and to theory. Please elaborate on this.

Response: We thank the Editor for this helpful comment. We have now elaborated on the analytic process to clarify how interpretations developed from initial coding through to theme development. In line with an Interpretative Phenomenological Analysis (IPA) approach, analysis involved close iterative, in-depth engagement with each transcript, beginning with detailed exploratory notes (descriptive, linguistic, and conceptual). These exploratory notes then informed the development of experiential statements, which were subsequently clustered into Personal Experiential Themes (PETs) for each participant. Cross-case analysis then enabled the development of Group Experiential Themes (GETs), which represent shared patterns of meaning across participants. We also clarify that IPA is not a theory-generating methodology, but an approach aiming at producing an interpretative account of lived experiences. This clarification has been added to the Methods section (Pages 11-13, Lines 260-298).

Comments from Reviewer 1

Comment 1: Line 177 - please resolve or complete this sentence to make it easy for readers to understand. e.g. thus for this study, adults self-managing coexisting physical LTCs with anxiety and/or depression was the phenomenon of interest/focus.

Response: We thank the reviewer for this comment. We have revised the sentence to improve clarity and readability, specifying that the phenomenon of interest in this study was adults self-managing coexisting physical long-term conditions alongside anxiety and/or depression (Page 8, Lines 194-195).

Comment 2: Line 178 - had a history or previously diagnosed with coexisting...

Response: We thank the reviewer for pointing this out. We have revised the wording for clarity and grammatical precision. This has been updated in the manuscript (Page 8-9, Lines 195-198).

Comment 3: Line 186 - Was the recruitment post disseminated via social media channels? which of the channels of dissemination yielded the highest number of participants?

Response: We thank the reviewer for this important point. The recruitment poster was disseminated via social media, and we have now specified the platform (Instagram) in the revised Methods section (Page 9, Line 206). However, recruitment sources were not systematically recorded by dissemination channels, as such it is not possible to determine which platform yielded the highest number of participants. This has been noted as a procedural limitation in the manuscript (Page 36, Lines 825-828).

Comment 4: Line 193 – sent out how? What platform/channel of transmission?

Response: We thank the reviewer for this comment. Participant consent forms were distributed to participants via email and once completed, participants returned them electronically via a secure online platform called SafeSend, prior to participation in the study. We have now clarified the consent procedure in the revised Methods section (Page 9, Lines 212-215).

Comment 5: Line 205 - Is there a reason for the inconsistency in dates? earlier you mentioned 3rd May - 31st July. Although you used the word between, but if possible, main the month of July for the sake of consistency.

Response: We thank the reviewer for pointing this out. Recruitment of participants took place between May and July, with a small number of interviews conducted in August due to participant availability and scheduling constraints. This provides clarification regarding the apparent inconsistency in the reporting.

Comment 6: Line 217 - Meetings with who? or between who?

Response: We thank this reviewer for this helpful comment. The meetings were held between the lead researcher (CL) and members of the PPIEP group. We have clarified the wording in the revised manuscript to avoid ambiguity (Page 10, Lines 238).

Comment 7: Any reference to support this or was it just a result from the discussions between PPIEP members?

Response: We thank the reviewer for this helpful comment. The changes to study design were informed by structured discussions with members of the PPIEP group,

rather than a specific external reference. This has been clarified within the revised Methods section (Page 10, Lines 241-242).

Comment 8: Line 232 - what was chosen by the participants? the pseudo-names?

Response: We thank the reviewer for this clarification comment. This refers to participants selecting their own pseudonyms to support anonymity. The manuscript has been revised to make this explicit (Page 11, Lines 253-255).

Comment 9: Thanks for including this. I was interested in learning why your study did not include any participant 35 years and above, and also why you had more females than males.

Response: We thank the reviewer for this insightful comment. The age range and female to male distribution reflect the characteristics of the eligible population who responded to the recruitment strategy and met the study inclusion criteria. No upper age limit of 35 years was set, and the sample composition may reflect self-selection bias. This has now been acknowledged in the Discussion section as a study limitation (Page 36, Lines 828-830).

Comment 10: On another note, thank you for suggesting future possible research. Would the authors consider incorporating strategies to capture a more representative demographic sample? This might allow for comparisons across age groups and gender, where appropriate. However, I recognize that such an expansion could become a separate research inquiry on its own.

Response: We thank the reviewer for this thoughtful suggestion. We agree that future research could benefit from recruiting a more demographically representative sample to facilitate comparisons across characteristics such as age and gender, where appropriate. In response to this comment, we have incorporated this recommendation into the Future Research section (Page 35, Lines 807-812), noting that future studies could employ a maximum variation sampling strategy during recruitment to capture a broader range of demographic and experiential characteristics.

<u>Comments from Reviewer 2</u>

Comment 1: I felt the description of mental health conditions could be thicker. Which mental health symptoms specifically affect self-management? How do they manage their mental health conditions? Does psycho-/pharmacotherapy help?

Response: We thank the reviewer for this insightful comment and respond to the points raised below:

- ***Mental health symptoms:*** We agree that providing a more detailed description of mental health symptomology strengthens the manuscript. The Introduction has been expanded to specify key symptoms (e.g. low mood, anxiety, fatigue, and reduced motivation) that may affect an individuals' capacity to self-manage alongside an added definition of mental health from the World Health Organization (2025) (Pages 5-6, Lines 125-131)
- ***Self-managing mental health conditions:*** We have further elaborated in the Introduction section on how individuals may self-manage mental health conditions, highlighting the complexity of managing coexisting physical and mental health conditions (Page 6, Lines 131-134; 137-141).
- ***Psycho/pharmacotherapy:*** We have also expanded the Introduction to reflect the role of psychological and pharmacological therapies in supporting self-management, while acknowledging that their effectiveness and accessibility may vary between individuals (Page 6, Lines 134-137). These additions strengthen the conceptual framing of mental health within the manuscript.

Comment 2: I felt it a bit odd that Discussion focuses on age a lot, even though it does not come up in the Introduction, Methods, or the earlier parts of the Results. Though the authors do mention the potential unintentional exclusion of older populations, it would be better to discuss this earlier and more extensively.

Response: We thank the reviewer for this thoughtful comment. We acknowledge that the emphasis on age in the Discussion may have appeared disproportionate given its more limited framing in earlier sections of the manuscript. This reflects the fact that the study was not designed with age as a primary focus, and therefore younger age was not foregrounded in the earlier sections of the manuscript.

However, the final sample comprised only younger adults, which may reflect limitations in the recruitment process. This became a defining feature of the dataset, and in turn, shaped the interpretation of the findings. We therefore considered this an important and unanticipated outcome, particularly given this represents a significant gap in the existing literature, as younger adults with coexisting physical and mental health long-term conditions remain underrepresented in self-management research. As such the prominence of younger participants with coexisting physical and mental health long-term conditions is thus presented as a novel contribution arising from the data rather than an a priori focus of the study.

In response to the reviewer's comment, we have revised the Discussion section to improve balance across themes, while retaining appropriate emphasis on age as a key contextual and novel feature of the findings. We believe these revisions improve

the coherence of the manuscript (Pages 29-34, Lines 643-659; 660-679, 697-701; 774-785).

Comment 3: Similarly, I feel that the conclusion of this manuscript ("The study found younger age to be a central inequality within self-management support, which crucially must be addressed…") may be too strong given that older populations were not included in the study. While it is true that young people face unique barriers as described in the manuscript, they probably also have advantages older people do not possess. A more balanced interpretation might be more appropriate.

Response: We thank the reviewer for this important comment. We agree that the original concluding statement was stronger than warranted given the study did not include participants from older age groups and therefore cannot support direct comparisons across the life course. In response, we have revised the Conclusion in both the manuscript (Page 37, Lines 834-846) and abstract (Page 3, Lines 57-62) to provide a more balanced interpretation of the findings. The revised Conclusion now emphasises that, while our findings highlight age-related factors may influence self-management among emerging adults living with coexisting physical and mental health long-term conditions, further research is needed to examine how these experiences may differ across age groups. This revision better reflects the scope of the data while retaining the novel contribution of the study.

Comment 4: The provided link for data seems inaccessible (it says "DOI Not Found"). Please double check.

Response: We thank the reviewer for highlighting the issue. We have checked the link and confirm that the DOI is not currently resolving. This is because, in line with the University of Southampton data management procedures, the dataset DOI is reserved during submission stage but is not formally minted or made publicly active until the dataset is finalised following manuscript acceptance.

To provide clarification, we have included a letter from the University's Research Data Management team within the submission materials outlining this process. This ensures transparency regarding the status of the DOI and the associated data governance procedures.

Comment 5: Did the participants receive any compensation?

Response: We thank the reviewer for this question. The research study activities were unfunded, and as such participants did not receive any financial or material compensation for their participation. This has now been clearly stated in the revised Methods section of the manuscript (Page 13, Lines 307-308).

Comment 6: Line 109 - Explanation on “diagnostic overshadowing” might be beneficial for some readers

Response: We thank the reviewer for this helpful suggestion. We agree that a brief explanation of “diagnostic overshadowing” would improve clarity for readers. We have therefore added a concise definition in the revised manuscript (Page 5, Lines 107-108), describing diagnostic overshadowing as the tendency for symptoms of physical health conditions to be misattributed to an existing mental health diagnosis, potentially leading to under-recognition or delayed diagnosis of physical illness.

Comment 7: Line 178 - What criteria were used for anxiety/depression? Who diagnosed?

Response: We thank the reviewer for this important comment. Eligibility for anxiety and/or depression was based on participants’ self-reported diagnosis. This approach was used to enhance accessibility and inclusivity, recognising variability in access to formal diagnostic services and differences in diagnostic recording within NHS pathways. This has now been clarified in the revised Methods section (Page 9, Lines 198-200).

Comment 8: Line 585 – What is DSA?

Response: We thank the reviewer for this clarification comment. We have now expanded the acronym of DSA in brackets next to the quotation (Page 28, Lines 624-625). DSA refers to Disabled Students’ Allowance, a UK government-funded support scheme to help students in higher education who have a disability, long-term health condition, mental health condition, or specific learning difficulty. It is not a cash payment to the student, rather it provides funding for extra study-related support and equipment.

Additional Clarifications

In addition to the above comments, all spelling and grammatical errors pointed out by the reviewers have been corrected.

We look forward to hearing from you regarding our submission and to respond to any further questions and comments you may have.

Sincerely,

Catherine Lodwick

03.06.2026

Click here to download Data Review URL
http://eprints.soton.ac.uk/512196/
