# Supplementary material for: "I want to take the power back": An interpretative phenomenological analysis of self-management in coexisting physical and mental long-term conditions
Source: PLoS One. 2026 Sep 25;21(9):e0359157. doi: 10.1371/journal.pone.0359157 (PMC13614564; doi:10.1371/journal.pone.0359157)
Supplement: Supplementary file 3 [file pone.0359157.s001.docx]

**S1 Checklist. Consolidated Criteria for Reporting Qualitative Studies (COREQ): 32-item checklist**

| **No** | **Item** | **Guide questions/description** | **Answer** |
| --- | --- | --- | --- |
| **Domain 1: Research team and reflexivity** | | | |
| ***Personal characteristics*** | | | |
| 1 | Interview/facilitator | Which author/s conducted the interview or focus group? | The first author conducted all of the interviews. |
| 2 | Credentials | What were the researcher’s credentials? E.g. PhD, MD | First author (CL): MNurs MRes.  Middle author (LA): PhD MSc BSc.  Last author (MCP): PhD MSc BSc FEANS. |
| 3 | Occupation | What was their occupation at the time of the study? | First author: Master Student.  Middle author (LA): Lecturer.  Last author: Professor. |
| 4 | Gender | Was the researcher male or female? | First author: Female. Not specified in the article. |
| 5 | Experience and training | What experience or training did the researcher have? | The researcher has previous academic experience in research. |
| ***Relationships with participants*** | | | |
| 6 | Relationship established | Was a relationship established prior to study commencement? | Only first contact to recruit participants and arrange the interview (via email). There is no therapeutic relationship between researchers and participants. |
| 7 | Participant knowledge of the interviewer | What did the participants know about the researcher? e.g. personal goals, reasons for doing the research | The participant information sheet explained the research in detail and was provided prior to arranging and conducting the interviews, with opportunities to ask further questions via email. |
| 8 | Interviewer characteristics | What characteristics were reported about the interviewer/facilitator? e.g. Bias, assumptions, reasons and interests in the research topic | The interest in the research topic was established in the participant information sheet. |
| **Domain 2: Study design** | | | |
| ***Theoretical framework*** | | | |
| 9 | Methodological orientation and theory | What methodological orientation was stated to underpin the study? e.g. grounded theory, discourse analysis, ethnography, phenomenology, content analysis | An interpretative phenomenological analysis approach was used to underpin the study. |
| ***Participant selection*** | | | |
| 10 | Purposive sampling was used to recruit participants to the study. | Purposive sampling was used to recruit participants to the study. | Purposive sampling was used to recruit participants to the study. |
| 11 | Social media, charitable organisations and recruitment posters in community hubs within the catchment areas (libraries and coffee shops. | Social media, charitable organisations and recruitment posters in community hubs within the catchment areas (libraries and coffee shops. | Social media, charitable organisations and recruitment posters in community hubs within the catchment areas (libraries and coffee shops). |
| 12 | 8 participants completed their interviews and were included in the study. | 8 participants completed their interviews and were included in the study. | 8 participants completed their interviews and were included in the study. |
| 13 | 1 participant did not attend their scheduled interview, and 1 participant did not follow up after initial correspondence and screening completion. | 1 participant did not attend their scheduled interview, and 1 participant did not follow up after initial correspondence and screening completion. | 1 participant did not attend their scheduled interview, and 1 participant did not follow up after initial correspondence and screening completion. |
| ***Setting*** | | | |
| 14 | Setting of data collection | Where was the data collected? e.g. home, clinic, workplace | Data was collected using an online platform – Microsoft Teams. Most of the participants were at home at the time of the interview. |
| 15 | Presence of non-participants | Was anyone else present besides the participants and researchers? | Both before and at the beginning of the interview it was recommended to be in a quiet, private space where neither the participant or researcher would be overheard and where minimal disruptions would be present to prevent risk of recording others who had not consented to being recorded. No other people were seen during the interviews. |
| 16 | Description of sample | What are the important characteristics of the sample? e.g. demographic data, date | Characteristics of participants that were collected included: age, physical long-term condition and mental long-term conditions. A table was created to visually illustrate to show these characteristics. |
| ***Data collection*** | | | |
| 17 | Interview guides | Were questions, prompts, guides provided by the authors? Was it pilot tested? | The questions were created by the first author, but feedback was sought and was used to refine the final questions from PPIEP participants and the researcher’s supervisor MCP. The questions were not provided to interview participants in advance. Prompts were used to ask additional open-ended questions to encourage detailed responses and assist with data collection. |
| 18 | Repeat interviews | Were repeat interviews carried out? If yes, how many? | No - all interviews were conducted once. |
| 19 | Audio/visual recording | Did the research use audio or visual recording to collect the data? | The interviews were audio and video recorded which was stored securely on a password protected computer and deleted upon completion of the transcription. |
| 20 | Field notes | Were field notes made during and/or after the interview or focus group? | No field notes were made during the interviews. |
| 21 | Duration | What was the duration of the interviews or focus group? | Interviews lasted between 40-70 minutes. |
| 22 | Data saturation | Was data saturation discussed? | Data saturation was not discussed. Information power was used and this is specified in the article. |
| 23 | Transcripts returned | Were transcripts returned to participants for comment and/or correction? | The transcripts were not returned to participants for comment and/or correction. |
| **Domain 3: Analysis and findings** | | | |
| ***Data analysis*** | | | |
| 24 | Number of data coders | How many data coders coded the data? | The first author coded all of the data. MCP provided feedback upon the analysis process and results from the first transcription to guide the analysis of other data. Authors met regularly to discuss GET development and ideas/changes were suggested where appropriate. |
| 25 | Description of the coding tree | Did authors provide a description of the coding tree? | No. |
| 26 | Derivation of themes | Were themes identified in advance or derived from the data? | Themes were derived and generated from the data. No themes were identified in advance. |
| 27 | Software | What software, if applicable, was used to manage the data? | No software was used to manage the data – the paper and pencil method was employed. Referred to in the article. |
| 28 | Participant checking | Did participants provide feedback on the findings? | No participant provided feedback on the research findings.  Referred to in the article. |
| ***Reporting*** | | | |
| 29 | Quotations presented | Were participant quotations presented to illustrate the themes/findings? Was each quotation identified? e.g. participant number | Quotations have been presented throughout the results section and participants have been anonymised using pseudonyms. |
| 30 | Data and findings consistent | Was there consistency between the data presented and the findings? | There is consistency between the data presented and the research findings. |
| 31 | Clarity of major themes | Were major themes clearly presented in the findings? | Presented in a table. |
| 32 | Clarity of minor themes | Is there a description of diverse cases or discussion of minor themes? | Presented in a table. |
