# Supplementary material for: "I want to take the power back": An interpretative phenomenological analysis of self-management in coexisting physical and mental long-term conditions
Source: PLoS One. 2026 Sep 25;21(9):e0359157. doi: 10.1371/journal.pone.0359157 (PMC13614564; doi:10.1371/journal.pone.0359157)
Supplement: Supplementary file 4 [file pone.0359157.s002.docx]

**S2 Checklist. GRIPP2_SF Reporting Checklist.**

| **Selection and topic** | **Item** |
| --- | --- |
| **1: Aim**  Report the aim of the study. | The aim of this qualitative study was to explore the facilitators and barriers to self-management for adults living with a physical long-term condition and coexisting anxiety or depression in the community. The aim of PPIEP in the study was to ensure the lived experiences of individuals living with coexisting physical long-term conditions and anxiety/depression were represented and informed the research design, data collection and interpretation of the findings with the hope to enhance the studies relevance and credibility. |
| **2: Methods**  Provide a clear description of the methods used for PPI in the study. | Two public contributors were recruited to assist at all stages of development for this research study. One member was a qualified mental health nurse who also had lived experiences of coexisting physical and mental health LTCs and the other is a service user from the BAME community who lives with coexisting physical and mental health LTCS providing a range of knowledge and experiences which contributed to the design and interpretation of the study.  The lead author met on a quarterly basis with each individual. These meetings were held both in-person and using online platforms to accommodate the members needs and preferences. The meets were planned to ask questions regarding the study design and to share findings from the study and gather feedback on the insights generated. These discussions were not focused on the individual’s diagnosis or medical history, and they were not required to disclose any personal information if they did not want to. Where required, follow-up conversations were held to refine materials or ask additional questions. Upon conclusion of the study informal feedback was sought on suggestions to mobilise knowledge and improving/refinement of future studies. This feedback was then incorporated into the current report in line with the principles of PPIEP. |
| **3: Results**  Outcomes – report the results of PPI in the study, including both the positive and negative outcomes. | PPIEP members contributed to the study in the following ways:   - Refining the focus and wording of the research question and study title. - Feedback on the methods for data collection e.g. face-to-face vs online platform vs telephone calls, thus influencing study design and subsequent ethics applications. - Comments provided on recruitment materials e.g. language, colours, layout, and location to advertise materials. - Guidance on topic guide development (language used in the questions, order of questions). - Interpretation of results and findings e.g. theme names, meaning behind the findings. These discussions informed the second iteration of theme names and order in which they appeared in the paper. - Providing feedback on knowledge mobilisation activities and future study directions to ensure sustainability of the research findings. |
| **4: Discussion**  Outcomes – comment on the extent to which PPI influenced the study overall. Describe the positive and negative effects. | Patient and public contributors for this study were very effective, offering important insights into all areas of the research process. For example, their feedback led to clearer participant-facing information and more accessible language within the study documents. A clear influence was the removal of a question from the interview topic guide which initially asked participants about their current health status. PPIEP members noted the interviews were often the first time visually meeting the researcher and therefore this many not have been an appropriate question to begin the interview with, ultimately influencing the rapport building and how open participants were throughout the interview. PPIEP members further enhanced the interpretation of findings by providing contextual insight based on their lived experiences, helping the research to remain grounded in real-world implications of the results and avoid over-medicalised wording in the theme names and subthemes. Some challenges were encountered during PPIEP involvement such as waiting on feedback if PPIEP members were provided documents to reflect and give feedback on. At times this extended certain stages of the study, as revisions required additional review and approval. In addition, there was a risk that only having two PPIEP contributors may not have fully represented the diversity of the wider population. Overall, while members involvement introduced some complexities to the study, its benefits in improving relevance and credibility of the study were considered to outweigh the challenges. |
| **5: Reflections**  Critical perspective – comment critically on the study, reflecting on the things that went well and those that did not so others can learn from this experience. | Reflecting on the PPIEP process, several strengths and limitations were identified. PPIEP contributors brough distinct and complementary perspectives to the study, shaped by both personal lived experiences and professional backgrounds. The inclusion of one white British and one BAME members strengthened the study by introducing cultural and experiential viewpoints, supporting a more inclusive consideration of study materials and interpretations. Early involvement of PPIEP members was a key strength as this enabled meaningful input on the study design and methodology at a formative stage of the project. However, as this project had no dedicated funding, the scope of PPIEP involvement was somewhat limited. Financial and geographical constraints meant one PPIEP member contributed remotely while the other engaged mainly in-person. While both members offered valuable insights, this separation limited the opportunities for interaction between members. In-person collaboration may have facilitated more dynamic discussions and produced different ideas particularly when exploring complex or sensitive aspects of the research. Future studies would benefit from including PPIEP members that could meet together, either in person or through online sessions, as well as being able to offer financial remuneration for their time and work. Overall, despite the limitations, the early and meaningful PPIEP involvement positively influenced the study, while highlighting the importance of adequate funding and logistical planning to maximise the impact of PPIEP perspectives. |
