# Supplementary material for: "I want to take the power back": An interpretative phenomenological analysis of self-management in coexisting physical and mental long-term conditions
Source: PLoS One. 2026 Sep 25;21(9):e0359157. doi: 10.1371/journal.pone.0359157 (PMC13614564; doi:10.1371/journal.pone.0359157)
Supplement: Supplementary file 5 [file pone.0359157.s005.docx]

**S1 Table. Interview Topic Guide.**

**Background**

- What are the current long-term conditions that you are living with?
- In your own words, what does the term self-management mean to you?

**Experiences of living with and self-managing coexisting long-term conditions**

- How does having coexisting physical and mental long-term conditions impact your health and wellbeing?
- What self-management activities do you do day-to-day to help manage your physical and mental long-term conditions?

**What helps and what hinders self-management abilities**

- What helps you to self-manage your coexisting long-term conditions?
- What limits/stops you from self-managing your coexisting long-term conditions?

**Changes to current self-management support**

- If you could, what changes you would like to make to current self-management support to better help you live with coexisting physical and mental long-term conditions?

**Round up and summary**

- An opportunity to add or share anything about your self-management experiences of living with long-term conditions that we haven’t spoken about yet?
