# Supplementary material for: "I want to take the power back": An interpretative phenomenological analysis of self-management in coexisting physical and mental long-term conditions
Source: PLoS One. 2026 Sep 25;21(9):e0359157. doi: 10.1371/journal.pone.0359157 (PMC13614564; doi:10.1371/journal.pone.0359157)
Supplement: Supplementary file 6 [file pone.0359157.s006.docx]

**S2 Table. Stages of Interpretative Phenomenological Analysis.**

| **Familiarity and engagement (key words/phrases)** | **Exploratory notes** | **Experiential statements** | **Personal Experiential Subthemes** | **Personal Experiential Themes** |
| --- | --- | --- | --- | --- |
| ‘I recognise wherever I’m going to one or the other that their expertise are in that area, which I understand, but I am not just anxious, I am not just fatty liver, I am a whole person with both of those things and many other things.’ | ‘Understand’ = expertise but not satisfied. ‘But I am…’ = conditions forming part of identity, not wanting to separate them into individual categories. Recognise individuals, beyond their conditions, can’t ignore one or the other – need holistic/integrated care to support all long-term conditions | Silo approach to self-management | Invisible illnesses | **I am a whole person** |
| ‘They did sort of talk about like the, the mental side of things. It was like a one, one of the weeks was dedicated to that but it all felt a little bit sort of tiptoeing around the subject, so I didn’t find it to be particularly good or effective at that or acknowledging that.’ | Whilst they did incorporate mental health ‘sort of’ suggests not well or effectively. Mental side – meaning ?separation. Indirect focus/not substantial enough for coexisting needs. Use of the word ‘tiptoeing’ infers hesitation/discomfort discussing it – use of the word ‘subject’ helps participants to feel detached form the topic? Needs explicit discussion, group leaders trained in mental health – improve confidence and discussion  facilitation. | Lack of existing and substantial support  Stigma | Devaluing mental health long-term conditions |  |
