# Supplementary material for: "I want to take the power back": An interpretative phenomenological analysis of self-management in coexisting physical and mental long-term conditions
Source: PLoS One. 2026 Sep 25;21(9):e0359157. doi: 10.1371/journal.pone.0359157 (PMC13614564; doi:10.1371/journal.pone.0359157)
Supplement: Supplementary file 7 [file pone.0359157.s007.docx]

**S3 Table. Participant contribution to Group Experiential Themes (GETs).**

| **Group Experiential Themes (GETs)** | | | | | | |
| --- | --- | --- | --- | --- | --- | --- |
|  | **Moving beyond sink or swim: scaffolding support to self-manage** | | **Self-managing: a choice or systemic exclusion** | | **Unseen struggles: learning to live with LTCs** | |
|  | ***“An internal battle”: Living with coexisting conditions*** | ***Seeing me, not just my long-term conditions*** | ***“You just feel lost”: Navigating the healthcare system*** | ***“Taking control” through self-management*** | ***My medical vs my social identity*** | ***Self-managing now for my future*** |
| **Rosemary** | X | X |  | X | X | X |
| **Grace** | X |  | X |  | X | X |
| **Poppy** | X | X | X | X | X | X |
| **Ruby** | X | X | X | X | X | X |
| **Oskar** | X | X |  | X | X | X |
| **Ashley** | X |  | X | X | X | X |
| **Laticia** | X | X | X | X | X | X |
| **Simone** | X | X | X | X | X | X |
| ***Names included are pseudonyms to maintain anonymity.*** | | | | | | |
