# Supplementary material for: "I want to take the power back": An interpretative phenomenological analysis of self-management in coexisting physical and mental long-term conditions
Source: PLoS One. 2026 Sep 25;21(9):e0359157. doi: 10.1371/journal.pone.0359157 (PMC13614564; doi:10.1371/journal.pone.0359157)
Supplement: Supplementary file 8 [file pone.0359157.s011.docx]

Dear Editor-in-Chief,

Thank you for giving us the opportunity to submit a revised draft of our manuscript titled ‘"I want to take the power back": An interpretative phenomenological analysis of self-management in coexisting physical and mental long-term condition’ to PLOS One. We appreciate the time and effort that the editors and the reviewers have dedicated to providing valuable feedback on the manuscript. We are grateful to the reviewers for their insightful comments on the paper. We have been able to incorporate changes to reflect most of the suggestions provided and these changes are highlighted within the manuscript.

Here is a point-by-point response to the journal, academic editor and reviewers’ comments and concerns.

**Journal Requirements**

**Comment 1:** Please ensure that your manuscript meets PLOS ONE's style requirements, including those for file naming.

**Response:** We have carefully reviewed the manuscript and revised the submission to ensure full compliance with PLOS One style requirements, including formatting and file naming conventions. All files have been checked and updated accordingly.

**Comment 2:** Thank you for uploading your study's underlying data set. Unfortunately, the repository you have noted in your Data Availability statement does not qualify as an acceptable data repository according to PLOS's standards. At this time, please upload the minimal data set necessary to replicate your study's findings to a stable, public repository (such as figshare or Dryad) and provide us with the relevant URLs, DOIs, or accession numbers that may be used to access these data.

**Response:** We thank the journal for this important comment. In line with institutional requirements and approved ethical procedures relating to participant confidentiality and data governance, the dataset is securely held within the University of Southampton’s institutional repository, PURE. An updated Data Availability Statement has now been completed to clarify the location of the data, and a letter from the University of Southampton Data Management Team has been attached detailing institutional procedures.

**Comment 3:** Please amend either the abstract on the online submission form (via Edit Submission) or the abstract in the manuscript so that they are identical.

**Response:** We thank the journal for this comment. We have carefully reviewed and amended the abstract in both the manuscript and online submission system to ensure that both versions are now identical.

**Comment 4:** We note that there is identifying data in the Supporting Information file <S3 Table. Participant contribution to GETs>. Due to the inclusion of these potentially identifying data, we have removed this file from your file inventory. Prior to sharing human research participant data, authors should consult with an ethics committee to ensure data are shared in accordance with participant consent and all applicable local laws.

**Response:** We thank the journal for this comment. The names included in ‘S3 Table. Participant contribution to GETs’ were participant pseudonyms rather than identifiable personal information, as stated within the manuscript. To further support clarity regarding confidentiality procedures, we have added a statement to the S3 Table explicitly confirming that all names are pseudonyms used to protect participant anonymity. The revised S3 Table has been updated and reuploaded accordingly.

**Comments from Editor**

**Comment 1:** Funding statement – please add funding/grant number.

**Response:** We thank the Editor for this comment. We have updated the Funding Statement to include the relevant funding source and grant number. The grant: NIHR304046 – Inspiring Students into Research (R1 2023) was awarded to the lead researcher (CL) which covered tuition fees and a stipend. This funding was not allocated to the specific research study.

**Comment 2:** Sample size is relatively small and heavily skewed (Male 1, female 8). This should be factored in the discussion and perhaps presented as part of study weakness.

**Response:** We thank the Editor for highlighting this issue. We agree that the sample size and gender imbalance are limitations of the study. This has now been acknowledged in the revised discussion section (Page 29, Lines 652-654) and included explicitly as a study limitation (Page 36, Lines 819-820, 828-830).

**Comment 3:** Socioeconomic, education/employment status are missing. These are critical determinants of self-management and inequality. Please attribute this to study weakness

**Response:** We thank the Editor for this important observation. We agree that socioeconomic status, education and employment are key determinants of self-management and health inequality. These variables were not collected in this study, and this limitation has now been explicitly stated as a study weakness (Page 36, Lines 830-833).

**Comment 4:** It is unclear how interpretations moved from codes to themes and to theory. Please elaborate on this.

**Response:** We thank the Editor for this helpful comment. We have now elaborated on the analytic process to clarify how interpretations developed from initial coding through to theme development. In line with an Interpretative Phenomenological Analysis (IPA) approach, analysis involved close iterative, in-depth engagement with each transcript, beginning with detailed exploratory notes (descriptive, linguistic, and conceptual). These exploratory notes then informed the development of experiential statements, which were subsequently clustered into Personal Experiential Themes (PETs) for each participant. Cross-case analysis then enabled the development of Group Experiential Themes (GETs), which represent shared patterns of meaning across participants. We also clarify that IPA is not a theory-generating methodology, but an approach aiming at producing an interpretative account of lived experiences. This clarification has been added to the Methods section (Pages 11-13, Lines 260-298).

**Comments from Reviewer 1**

**Comment 1:** Line 177 - please resolve or complete this sentence to make it easy for readers to understand. e.g. thus for this study, adults self-managing coexisting physical LTCs with anxiety and/or depression was the phenomenon of interest/focus.

**Response:** We thank the reviewer for this comment. We have revised the sentence to improve clarity and readability, specifying that the phenomenon of interest in this study was adults self-managing coexisting physical long-term conditions alongside anxiety and/or depression (Page 8, Lines 194-195).

**Comment 2:** Line 178 - had a history or previously diagnosed with coexisting...

**Response:** We thank the reviewer for pointing this out. We have revised the wording for clarity and grammatical precision. This has been updated in the manuscript (Page 8-9, Lines 195-198).

**Comment 3:** Line 186 - Was the recruitment post disseminated via social media channels? which of the channels of dissemination yielded the highest number of participants?

**Response:** We thank the reviewer for this important point. The recruitment poster was disseminated via social media, and we have now specified the platform (Instagram) in the revised Methods section (Page 9, Line 206). However, recruitment sources were not systematically recorded by dissemination channels, as such it is not possible to determine which platform yielded the highest number of participants. This has been noted as a procedural limitation in the manuscript (Page 36, Lines 825-828).

**Comment 4:** Line 193 – sent out how? What platform/channel of transmission?

**Response:** We thank the reviewer for this comment. Participant consent forms were distributed to participants via email and once completed, participants returned them electronically via a secure online platform called SafeSend, prior to participation in the study. We have now clarified the consent procedure in the revised Methods section (Page 9, Lines 212-215).

**Comment 5:** Line 205 - Is there a reason for the inconsistency in dates? earlier you mentioned 3rd May - 31st July. Although you used the word between, but if possible, main the month of July for the sake of consistency.

**Response:** We thank the reviewer for pointing this out. Recruitment of participants took place between May and July, with a small number of interviews conducted in August due to participant availability and scheduling constraints. This provides clarification regarding the apparent inconsistency in the reporting.

**Comment 6:** Line 217 - Meetings with who? or between who?

**Response:** We thank this reviewer for this helpful comment. The meetings were held between the lead researcher (CL) and members of the PPIEP group. We have clarified the wording in the revised manuscript to avoid ambiguity (Page 10, Lines 238).

**Comment 7:** Any reference to support this or was it just a result from the discussions between PPIEP members?

**Response:** We thank the reviewer for this helpful comment. The changes to study design were informed by structured discussions with members of the PPIEP group, rather than a specific external reference. This has been clarified within the revised Methods section (Page 10, Lines 241-242).

**Comment 8:** Line 232 - what was chosen by the participants? the pseudo-names?

**Response:** We thank the reviewer for this clarification comment. This refers to participants selecting their own pseudonyms to support anonymity. The manuscript has been revised to make this explicit (Page 11, Lines 253-255).

**Comment 9:** Thanks for including this. I was interested in learning why your study did not include any participant 35 years and above, and also why you had more females than males.

**Response:** We thank the reviewer for this insightful comment. The age range and female to male distribution reflect the characteristics of the eligible population who responded to the recruitment strategy and met the study inclusion criteria. No upper age limit of 35 years was set, and the sample composition may reflect self-selection bias. This has now been acknowledged in the Discussion section as a study limitation (Page 36, Lines 828-830).

**Comment 10:** On another note, thank you for suggesting future possible research. Would the authors consider incorporating strategies to capture a more representative demographic sample? This might allow for comparisons across age groups and gender, where appropriate. However, I recognize that such an expansion could become a separate research inquiry on its own.

Response: We thank the reviewer for this thoughtful suggestion. We agree that future research could benefit from recruiting a more demographically representative sample to facilitate comparisons across characteristics such as age and gender, where appropriate. In response to this comment, we have incorporated this recommendation into the Future Research section (Page 35, Lines 807-812), noting that future studies could employ a maximum variation sampling strategy during recruitment to capture a broader range of demographic and experiential characteristics.

**Comments from Reviewer 2**

**Comment 1:** I felt the description of mental health conditions could be thicker. Which mental health symptoms specifically affect self-management? How do they manage their mental health conditions? Does psycho-/pharmacotherapy help?

**Response:** We thank the reviewer for this insightful comment and respond to the points raised below:

- ***Mental health symptoms:*** We agree that providing a more detailed description of mental health symptomology strengthens the manuscript. The Introduction has been expanded to specify key symptoms (e.g. low mood, anxiety, fatigue, and reduced motivation) that may affect an individuals’ capacity to self-manage alongside an added definition of mental health from the World Health Organization (2025) (Pages 5-6, Lines 125-131)
- ***Self-managing mental health conditions:*** We have further elaborated in the Introduction section on how individuals may self-manage mental health conditions, highlighting the complexity of managing coexisting physical and mental health conditions (Page 6, Lines 131-134; 137-141).
- ***Psycho/pharmacotherapy:*** We have also expanded the Introduction to reflect the role of psychological and pharmacological therapies in supporting self-management, while acknowledging that their effectiveness and accessibility may vary between individuals (Page 6, Lines 134-137). These additions strengthen the conceptual framing of mental health within the manuscript.

**Comment 2:** I felt it a bit odd that Discussion focuses on age a lot, even though it does not come up in the Introduction, Methods, or the earlier parts of the Results. Though the authors do mention the potential unintentional exclusion of older populations, it would be better to discuss this earlier and more extensively.

**Response:** We thank the reviewer for this thoughtful comment. We acknowledge that the emphasis on age in the Discussion may have appeared disproportionate given its more limited framing in earlier sections of the manuscript. This reflects the fact that the study was not designed with age as a primary focus, and therefore younger age was not foregrounded in the earlier sections of the manuscript.

However, the final sample comprised only younger adults, which may reflect limitations in the recruitment process. This became a defining feature of the dataset, and in turn, shaped the interpretation of the findings. We therefore considered this an important and unanticipated outcome, particularly given this represents a significant gap in the existing literature, as younger adults with coexisting physical and mental health long-term conditions remain underrepresented in self-management research. As such the prominence of younger participants with coexisting physical and mental health long-term conditions is thus presented as a novel contribution arising from the data rather than an a priori focus of the study.

In response to the reviewer’s comment, we have revised the Discussion section to improve balance across themes, while retaining appropriate emphasis on age as a key contextual and novel feature of the findings. We believe these revisions improve the coherence of the manuscript (Pages 29-34, Lines 643-659; 660-679, 697-701; 774-785).

**Comment 3:** Similarly, I feel that the conclusion of this manuscript (“The study found younger age to be a central inequality within self-management support, which crucially must be addressed…”) may be too strong given that older populations were not included in the study. While it is true that young people face unique barriers as described in the manuscript, they probably also have advantages older people do not possess. A more balanced interpretation might be more appropriate.

**Response:** We thank the reviewer for this important comment. We agree that the original concluding statement was stronger than warranted given the study did not include participants from older age groups and therefore cannot support direct comparisons across the life course. In response, we have revised the Conclusion in both the manuscript (Page 37, Lines 834-846) and abstract (Page 3, Lines 57-62) to provide a more balanced interpretation of the findings. The revised Conclusion now emphasises that, while our findings highlight age-related factors may influence self-management among emerging adults living with coexisting physical and mental health long-term conditions, further research is needed to examine how these experiences may differ across age groups. This revision better reflects the scope of the data while retaining the novel contribution of the study.

**Comment 4:** The provided link for data seems inaccessible (it says "DOI Not Found"). Please double check.

**Response:** We thank the reviewer for highlighting the issue. We have checked the link and confirm that the DOI is not currently resolving. This is because, in line with the University of Southampton data management procedures, the dataset DOI is reserved during submission stage but is not formally minted or made publicly active until the dataset is finalised following manuscript acceptance.

To provide clarification, we have included a letter from the University’s Research Data Management team within the submission materials outlining this process. This ensures transparency regarding the status of the DOI and the associated data governance procedures.

**Comment 5:** Did the participants receive any compensation?

**Response:** We thank the reviewer for this question. The research study activities were unfunded, and as such participants did not receive any financial or material compensation for their participation. This has now been clearly stated in the revised Methods section of the manuscript (Page 13, Lines 307-308).

**Comment 6:** Line 109 - Explanation on “diagnostic overshadowing” might be beneficial for some readers

**Response:** We thank the reviewer for this helpful suggestion. We agree that a brief explanation of “diagnostic overshadowing” would improve clarity for readers. We have therefore added a concise definition in the revised manuscript (Page 5, Lines 107-108), describing diagnostic overshadowing as the tendency for symptoms of physical health conditions to be misattributed to an existing mental health diagnosis, potentially leading to under-recognition or delayed diagnosis of physical illness.

**Comment 7:** Line 178 - What criteria were used for anxiety/depression? Who diagnosed?

**Response:** We thank the reviewer for this important comment. Eligibility for anxiety and/or depression was based on participants’ self-reported diagnosis. This approach was used to enhance accessibility and inclusivity, recognising variability in access to formal diagnostic services and differences in diagnostic recording within NHS pathways. This has now been clarified in the revised Methods section (Page 9, Lines 198-200).

**Comment 8:** Line 585 – What is DSA?

**Response:** We thank the reviewer for this clarification comment. We have now expanded the acronym of DSA in brackets next to the quotation (Page 28, Lines 624-625). DSA refers to Disabled Students’ Allowance, a UK government-funded support scheme to help students in higher education who have a disability, long-term health condition, mental health condition, or specific learning difficulty. It is not a cash payment to the student, rather it provides funding for extra study-related support and equipment.

**Additional Clarifications**

In addition to the above comments, all spelling and grammatical errors pointed out by the reviewers have been corrected.

We look forward to hearing from you regarding our submission and to respond to any further questions and comments you may have.

Sincerely,

Catherine Lodwick

03.06.2026
