# Supplementary material for: "I want to take the power back": An interpretative phenomenological analysis of self-management in coexisting physical and mental long-term conditions
Source: PLoS One. 2026 Sep 25;21(9):e0359157. doi: 10.1371/journal.pone.0359157 (PMC13614564; doi:10.1371/journal.pone.0359157)
Supplement: Supplementary file 9 [file pone.0359157.s012.docx]

Dear Editor-in-Chief,

Thank you for the opportunity to submit a revised version of our manuscript, “I want to take the power back”: An Interpretative Phenomenological Analysis of Self-Management in Coexisting Physical and Mental Long-Term Conditions, to PLOS ONE. We sincerely appreciate the time and effort invested by the editors and reviewers in evaluating our work and providing constructive feedback. Their insightful comments have been invaluable in strengthening the manuscript. We have carefully considered the recommendations and have incorporated most of the suggested revisions. All changes are highlighted within the revised manuscript. Below, we provide a detailed, point-by-point response to the reviewers’ comments.

**Comments from Reviewer 1**

**Comment 1:** Line 61 - 'theneed' appearing in the online portal abstract

**Response:** We thank the reviewer for identifying this typo error. The term 'theneed' in the abstract on the online portal has been corrected to 'the need' (Page 3, Line 60).

**Comment 2:** Line 106 - Please delete this single quotation mark on "individuals'".

**Response:** We thank the reviewer for pointing this out. The single quotation mark has been deleted (Page 5, Line 106).

**Comment 3:** Line 112-114 - "Most recently observed..." Is this sentence complete?

**Response:** We thank the reviewer for highlighting this issue. The sentence has been revised for clarity and completeness (Page 5, Lines 112-114).

**Comment 4:** Line 153-156 - "Despite the recognition..." Do you think the full stop should be placed here? It makes the sentence read as incomplete.

Response: We thank the reviewer for this observation. We agree that the full stop resulted in an incomplete sentence. The text has been revised to ensure grammatical completeness and improve readability (Page 7, Lines 153-159).

**Comment 5:** Line 169 – To remove the 'a' before person-centred and contextually responsive.

**Response:** We thank the reviewer for pointing this out. The "a" has been removed to improve grammatical accuracy (Page 7, Line 169).

**Comment 6:** Line 170 - "self-management interventions" Do you think this needs to be in singular or plural form?

**Response:** We thank this reviewer for this helpful observation. We agree that the singular form is more appropriate in this context, this has now been rectified (Page 7, Line 170).

**Comment 7:** Line 326 - "allowing for more in-depth interpretation opposed to superficial understanding" Should this be as opposed to?

**Response:** We thank the reviewer for this suggestion. We agree that the phrase "as opposed to" provides a clearer and more grammatically complete comparison in this context. The text has been amended accordingly (Page 14, Line 326).

**Comment 8:** Line 741 - "MLTCs" I think this is the first time I am coming across this acronym. Can you please define this before using it? I see it appears only 2ce in the entire document...

**Response:** We thank the reviewer for this clarification comment. As the term appears only twice within the manuscript, the acronym has been removed, and multiple long-term conditions is now written in full throughout for improved clarity and readability (Page 34, Line 741).

**Comment 9:** Line 818 - should there be an inverted coma after the letter s' on "participants"

**Response:** We thank the reviewer for this comment. The apostrophe has now been corrected to "participants' lived experiences" (Page 38, Line 819).

**Comment 10:** Line 823-830 - should this be Secondly? Thirdly?

**Response:** We thank the reviewer for highlighting this inconsistency. The structure has now been revised to ensure consistency throughout the strengths and limitations paragraph (Page 38, Lines 824-837).

**Comment 11:** Line 838 - typo "person-cantered"

**Response:** We thank the reviewer for identifying this typo. The term "person-cantered" has been corrected to "person-centred" in the conclusion (Page 39, Line 842).

**Comments from Reviewer 2**

**Comment 1:** My intention was more about whether your interview data provide insight into these issues. I don't feel very strongly about this, but please kindly add findings in this area if you have any. If not, please leave it as is. I also appreciate your added description in the Introduction.

**Response:** We thank the reviewer for this important and insightful comment. We agree that this is a valuable consideration and has important implications for future research in this area. However, the current study did not specifically collect data relating to this aspect, as such we are unable to explore it further within the present manuscript. We have therefore retained the original text but will incorporate this consideration into the design of future studies in this area where it may help to further develop understanding of the topic.

**Comment 2:** Thank you for the clarification. Is it self-report of diagnosis made by health workers, or does this include self-diagnosis? In other words, "I was diagnosed with depression (by a health worker)" or "(I think) I have depression"? I think this detail is important to know more accurately about the participant population. If the latter, it should probably be noted as a limitation, as it is harder to ascertain the nature of mental health conditions of the participants.

**Response:** Thank you for this important observation. Participation eligibility was based on self-diagnosis/identification of a mental health condition. As such, participants may have either received a diagnosis from a healthcare professional or identified themselves as having anxiety and/or depression without formal diagnosis. We relied on participants self-reports and did not independently confirm diagnostic status. To improve clarity the terminology has been changed from self-reported to self-diagnosis (Page 9, Line 198), and this has now been acknowledged as a study limitation in the manuscript (Page 38, Line 831-834).

**Comment 3:** Line 838 - typo "person-cantered"

**Response:** We thank the reviewer for identifying this typo. The term "person-cantered" has been corrected to "person-centred" in the conclusion (Page 39, Line 842).

We look forward to hearing from you regarding our submission and to respond to any further questions and comments you may have.

Sincerely,

Catherine Lodwick

07.08.2026
