# Supplementary material for: "I want to take the power back": An interpretative phenomenological analysis of self-management in coexisting physical and mental long-term conditions
Source: PLoS One. 2026 Sep 25;21(9):e0359157. doi: 10.1371/journal.pone.0359157 (PMC13614564; doi:10.1371/journal.pone.0359157)
Supplement: Peer review history

# Peer review: Decision Letter 0

**DOI:** 10.1371/journal.pone.0359157.r001

**Authors:** Ibrahim Jahun

24 Apr 2026

Dear Dr. Lodwick,

- Funding statement: Please add funding/grant number
- Technical observations:Sample size is relatively small and heavily skewed (Male 1, female 8). This should be factored in the discussion and perhaps presented as part of study weakness
- Socioeconomic, education/employment status are missing. These are critical determinants of self-management and inequality. Please attribute this to study weakness
- It is unclear how interpretations moved from codes to themes and to theory. Please elaborate on this.

==============================

Please submit your revised manuscript by Jun 08 2026 11:59PM. If you will need more time than this to complete your revisions, please reply to this message or contact the journal office at plosone@plos.org. When you’re ready to submit your revision, log on to https://www.editorialmanager.com/pone/ and select the 'Submissions Needing Revision' folder to locate your manuscript file.

Please include the following items when submitting your revised manuscript:

- A letter that responds to each point raised by the academic editor and reviewer(s). You should upload this letter as a separate file labeled 'Response to Reviewers'.
- A marked-up copy of your manuscript that highlights changes made to the original version. You should upload this as a separate file labeled 'Revised Manuscript with Track Changes'.
- An unmarked version of your revised paper without tracked changes. You should upload this as a separate file labeled 'Manuscript'.

If you would like to make changes to your financial disclosure, please include your updated statement in your cover letter. Guidelines for resubmitting your figure files are available below the reviewer comments at the end of this letter.

As the corresponding author, your ORCID iD is verified in the submission system and will appear in the published article. PLOS supports the use of ORCID, and we encourage all coauthors to register for an ORCID iD and use it as well. Please encourage your coauthors to verify their ORCID iD within the submission system before final acceptance, as unverified ORCID iDs will not appear in the published article. *Only*  the individual author can complete the verification step; PLOS staff *cannot*  verify ORCID iDs on behalf of authors.

We look forward to receiving your revised manuscript.

Kind regards,

Ibrahim Jahun, MD, MSC, PhD

Academic Editor

PLOS One

**Journal Requirements:**

1. When submitting your revision, we need you to address these additional requirements.

Please ensure that your manuscript meets PLOS ONE’s style requirements, including those for file naming. The PLOS ONE style templates can be found at

https://journals.plos.org/plosone/s/file?id=wjVg/PLOSOne_formatting_sample_main_body.pdf and

https://journals.plos.org/plosone/s/file?id=ba62/PLOSOne_formatting_sample_title_authors_affiliations.pdf

2. Thank you for uploading your study’s underlying data set. Unfortunately, the repository you have noted in your Data Availability statement does not qualify as an acceptable data repository according to PLOS’s standards.

At this time, please upload the minimal data set necessary to replicate your study’s findings to a stable, public repository (such as figshare or Dryad) and provide us with the relevant URLs, DOIs, or accession numbers that may be used to access these data. For a list of recommended repositories and additional information on PLOS standards for data deposition, please see https://journals.plos.org/plosone/s/recommended-repositories.

3. Please amend either the abstract on the online submission form (via Edit Submission) or the abstract in the manuscript so that they are identical.

4. We note that there is identifying data in the Supporting Information file <S3 Table. Participant contribution to GETs>. Due to the inclusion of these potentially identifying data, we have removed this file from your file inventory. Prior to sharing human research participant data, authors should consult with an ethics committee to ensure data are shared in accordance with participant consent and all applicable local laws.

Data sharing should never compromise participant privacy. It is therefore not appropriate to publicly share personally identifiable data on human research participants. The following are examples of data that should not be shared:

-Name, initials, physical address

-Ages more specific than whole numbers

-Internet protocol (IP) address

-Specific dates (birth dates, death dates, examination dates, etc.)

-Contact information such as phone number or email address

-Location data

-ID numbers that seem specific (long numbers, include initials, titled “Hospital ID”) rather than random (small numbers in numerical order)

Data that are not directly identifying may also be inappropriate to share, as in combination they can become identifying. For example, data collected from a small group of participants, vulnerable populations, or private groups should not be shared if they involve indirect identifiers (such as sex, ethnicity, location, etc.) that may risk the identification of study participants.

Additional guidance on preparing raw data for publication can be found in our Data Policy (https://journals.plos.org/plosone/s/data-availability#loc-human-research-participant-data-and-other-sensitive-data) and in the following article: http://www.bmj.com/content/340/bmj.c181.long.

Please remove or anonymize all personal information (<specific identifying information in file to be removed>), ensure that the data shared are in accordance with participant consent, and re-upload a fully anonymized data set. Please note that spreadsheet columns with personal information must be removed and not hidden as all hidden columns will appear in the published file.

5. If the reviewer comments include a recommendation to cite specific previously published works, please review and evaluate these publications to determine whether they are relevant and should be cited. There is no requirement to cite these works unless the editor has indicated otherwise. 

6. Please review your reference list to ensure that it is complete and correct. If you have cited papers that have been retracted, please include the rationale for doing so in the manuscript text, or remove these references and replace them with relevant current references. Any changes to the reference list should be mentioned in the rebuttal letter that accompanies your revised manuscript. If you need to cite a retracted article, indicate the article’s retracted status in the References list and also include a citation and full reference for the retraction notice.

[Note: HTML markup is below. Please do not edit.]

Reviewers' comments:

Reviewer’s Responses to Questions

**Comments to the Author**

1. Is the manuscript technically sound, and do the data support the conclusions?

Reviewer #1: Yes

Reviewer #2: Partly

**********

2. Has the statistical analysis been performed appropriately and rigorously?

Reviewer #1: N/A

Reviewer #2: N/A

**********

3. Have the authors made all data underlying the findings in their manuscript fully available?

The PLOS Data policy

Reviewer #1: Yes

Reviewer #2: No

**********

4. Is the manuscript presented in an intelligible fashion and written in standard English?

Reviewer #1: Yes

Reviewer #2: Yes

**********

**Reviewer #1:** The topic is highly relevant, and I truly appreciate the authors for the thorough work put into this paper. The writing style is consistent, the flow is smooth, and the content is easy to read. I noted a few minor corrections and small typos that, once addressed, will further improve the reader’s experience. My comments and questions are included directly in the attached document. You’ll find them marked in the color‑highlighted sections for easy reference.

On another note, thank you for suggesting future possible research. Would the authors consider incorporating strategies to capture a more representative demographic sample? This might allow for comparisons across age groups and gender, where appropriate. However, I recognize that such an expansion could become a separate research inquiry on its own.

Overall, I have no major concerns, this is a strong piece.

**Reviewer #2:** Thank you for the opportunity to review this manuscript. It focuses on self-management in coexisting physical and mental long-term conditions, which has not been given enough attention previously.

The use of interpretive phenomenological analysis is appropriate for the aim of gaining in-depth understanding of lived experience, and the method is described in detail. Results are also carefully elaborated. Overall, the manuscript adds to the understanding of self-management in this population. I feel with minor revision this manuscript will be suitable for publication.

I would like to provide some suggestions to potentially improve the manuscript.

- I felt the description of mental health conditions could be thicker. Which mental health symptoms specifically affect self-management? How do they manage their mental health conditions? Does psycho-/pharmacotherapy help?

- I felt it a bit odd that Discussion focuses on age a lot, even though it does not come up in the Introduction, Methods, or the earlier parts of the Results. Though the authors do mention the potential unintentional exclusion of older populations, it would be better to discuss this earlier and more extensively.

- Similarly, I feel that the conclusion of this manuscript (“The study found younger age to be a central inequality within self-management support, which crucially must be addressed…”) may be too strong given that older populations were not included in the study. While it is true that young people face unique barriers as described in the manuscript, they probably also have advantages older people do not possess. A more balanced interpretation might be more appropriate.

Other minor points:

- The provided link for data seems inaccessible (it says "DOI Not Found"). Please double check

https://doi.org/10.5258/SOTON/D3861

- Did the participants receive any compensation?

109: Explanation on “diagnostic overshadowing” might be beneficial for some readers

125: “Despite an increasing evidence-based on self-management interventions” – typo?

178: What criteria were used for anxiety/depression? Who diagnosed?

215: spell out BAME

317: “All participants went onto note” – typo?

349: spell out WRAP (probably in brackets)

585: what is DSA?

**********

what does this mean?). If published, this will include your full peer review and any attached files.

If you choose “no”, your identity will remain anonymous but your review may still be made public.

**Do you want your identity to be public for this peer review?**  For information about this choice, including consent withdrawal, please see our Privacy Policy

Reviewer #1: **Yes:** Ezinne Sylvia Melikam

Reviewer #2: No

**********

[NOTE: If reviewer comments were submitted as an attachment file, they will be attached to this email and accessible via the submission site. Please log into your account, locate the manuscript record, and check for the action link "View Attachments". If this link does not appear, there are no attachment files.]

To ensure your figures meet our technical requirements, please review our figure guidelines: https://journals.plos.org/plosone/s/figures

You may also use PLOS’s free figure tool, NAAS, to help you prepare publication quality figures: https://journals.plos.org/plosone/s/figures#loc-tools-for-figure-preparation.

NAAS will assess whether your figures meet our technical requirements by comparing each figure against our figure specifications.

Attachment Submitted filename: PONE-D-26-09215_ESM_reviewed.pdf

# Author comment: Author response to Decision Letter 1

**DOI:** 10.1371/journal.pone.0359157.r002

24 Jun 2026

Comments from Editor

Comment 1: Funding statement – please add funding/grant number.

Response: We thank the Editor for this comment. We have updated the Funding Statement to include the relevant funding source and grant number. The grant: NIHR304046 – Inspiring Students into Research (R1 2023) was awarded to the lead researcher (CL) which covered tuition fees and a stipend. This funding was not allocated to the specific research study.

Comment 2: Sample size is relatively small and heavily skewed (Male 1, female 8). This should be factored in the discussion and perhaps presented as part of study weakness.

Response: We thank the Editor for highlighting this issue. We agree that the sample size and gender imbalance are limitations of the study. This has now been acknowledged in the revised discussion section (Page 29, Lines 652-654) and included explicitly as a study limitation (Page 36, Lines 819-820, 828-830).

Comment 3: Socioeconomic, education/employment status are missing. These are critical determinants of self-management and inequality. Please attribute this to study weakness

Response: We thank the Editor for this important observation. We agree that socioeconomic status, education and employment are key determinants of self-management and health inequality. These variables were not collected in this study, and this limitation has now been explicitly stated as a study weakness (Page 36, Lines 830-833).

Comment 4: It is unclear how interpretations moved from codes to themes and to theory. Please elaborate on this.

Response: We thank the Editor for this helpful comment. We have now elaborated on the analytic process to clarify how interpretations developed from initial coding through to theme development. In line with an Interpretative Phenomenological Analysis (IPA) approach, analysis involved close iterative, in-depth engagement with each transcript, beginning with detailed exploratory notes (descriptive, linguistic, and conceptual). These exploratory notes then informed the development of experiential statements, which were subsequently clustered into Personal Experiential Themes (PETs) for each participant. Cross-case analysis then enabled the development of Group Experiential Themes (GETs), which represent shared patterns of meaning across participants. We also clarify that IPA is not a theory-generating methodology, but an approach aiming at producing an interpretative account of lived experiences. This clarification has been added to the Methods section (Pages 11-13, Lines 260-298).

Comments from Reviewer 1

Comment 1: Line 177 - please resolve or complete this sentence to make it easy for readers to understand. e.g. thus for this study, adults self-managing coexisting physical LTCs with anxiety and/or depression was the phenomenon of interest/focus.

Response: We thank the reviewer for this comment. We have revised the sentence to improve clarity and readability, specifying that the phenomenon of interest in this study was adults self-managing coexisting physical long-term conditions alongside anxiety and/or depression (Page 8, Lines 194-195).

Comment 2: Line 178 - had a history or previously diagnosed with coexisting...

Response: We thank the reviewer for pointing this out. We have revised the wording for clarity and grammatical precision. This has been updated in the manuscript (Page 8-9, Lines 195-198).

Comment 3: Line 186 - Was the recruitment post disseminated via social media channels? which of the channels of dissemination yielded the highest number of participants?

Response: We thank the reviewer for this important point. The recruitment poster was disseminated via social media, and we have now specified the platform (Instagram) in the revised Methods section (Page 9, Line 206). However, recruitment sources were not systematically recorded by dissemination channels, as such it is not possible to determine which platform yielded the highest number of participants. This has been noted as a procedural limitation in the manuscript (Page 36, Lines 825-828).

Comment 4: Line 193 – sent out how? What platform/channel of transmission?

Response: We thank the reviewer for this comment. Participant consent forms were distributed to participants via email and once completed, participants returned them electronically via a secure online platform called SafeSend, prior to participation in the study. We have now clarified the consent procedure in the revised Methods section (Page 9, Lines 212-215).

Comment 5: Line 205 - Is there a reason for the inconsistency in dates? earlier you mentioned 3rd May - 31st July. Although you used the word between, but if possible, main the month of July for the sake of consistency.

Response: We thank the reviewer for pointing this out. Recruitment of participants took place between May and July, with a small number of interviews conducted in August due to participant availability and scheduling constraints. This provides clarification regarding the apparent inconsistency in the reporting.

Comment 6: Line 217 - Meetings with who? or between who?

Response: We thank this reviewer for this helpful comment. The meetings were held between the lead researcher (CL) and members of the PPIEP group. We have clarified the wording in the revised manuscript to avoid ambiguity (Page 10, Lines 238).

Comment 7: Any reference to support this or was it just a result from the discussions between PPIEP members?

Response: We thank the reviewer for this helpful comment. The changes to study design were informed by structured discussions with members of the PPIEP group, rather than a specific external reference. This has been clarified within the revised Methods section (Page 10, Lines 241-242).

Comment 8: Line 232 - what was chosen by the participants? the pseudo-names?

Response: We thank the reviewer for this clarification comment. This refers to participants selecting their own pseudonyms to support anonymity. The manuscript has been revised to make this explicit (Page 11, Lines 253-255).

Comment 9: Thanks for including this. I was interested in learning why your study did not include any participant 35 years and above, and also why you had more females than males.

Response: We thank the reviewer for this insightful comment. The age range and female to male distribution reflect the characteristics of the eligible population who responded to the recruitment strategy and met the study inclusion criteria. No upper age limit of 35 years was set, and the sample composition may reflect self-selection bias. This has now been acknowledged in the Discussion section as a study limitation (Page 36, Lines 828-830).

Comment 10: On another note, thank you for suggesting future possible research. Would the authors consider incorporating strategies to capture a more representative demographic sample? This might allow for comparisons across age groups and gender, where appropriate. However, I recognize that such an expansion could become a separate research inquiry on its own.

Response: We thank the reviewer for this thoughtful suggestion. We agree that future research could benefit from recruiting a more demographically representative sample to facilitate comparisons across characteristics such as age and gender, where appropriate. In response to this comment, we have incorporated this recommendation into the Future Research section (Page 35, Lines 807-812), noting that future studies could employ a maximum variation sampling strategy during recruitment to capture a broader range of demographic and experiential characteristics.

Comments from Reviewer 2

Comment 1: I felt the description of mental health conditions could be thicker. Which mental health symptoms specifically affect self-management? How do they manage their mental health conditions? Does psycho-/pharmacotherapy help?

Response: We thank the reviewer for this insightful comment and respond to the points raised below:

Mental health symptoms: We agree that providing a more detailed description of mental health symptomology strengthens the manuscript. The Introduction has been expanded to specify key symptoms (e.g. low mood, anxiety, fatigue, and reduced motivation) that may affect an individuals’ capacity to self-manage alongside an added definition of mental health from the World Health Organization (2025) (Pages 5-6, Lines 125-131)

Self-managing mental health conditions: We have further elaborated in the Introduction section on how individuals may self-manage mental health conditions, highlighting the complexity of managing coexisting physical and mental health conditions (Page 6, Lines 131-134; 137-141).

Psycho/pharmacotherapy: We have also expanded the Introduction to reflect the role of psychological and pharmacological therapies in supporting self-management, while acknowledging that their effectiveness and accessibility may vary between individuals (Page 6, Lines 134-137). These additions strengthen the conceptual framing of mental health within the manuscript.

Comment 2: I felt it a bit odd that Discussion focuses on age a lot, even though it does not come up in the Introduction, Methods, or the earlier parts of the Results. Though the authors do mention the potential unintentional exclusion of older populations, it would be better to discuss this earlier and more extensively.

Response: We thank the reviewer for this thoughtful comment. We acknowledge that the emphasis on age in the Discussion may have appeared disproportionate given its more limited framing in earlier sections of the manuscript. This reflects the fact that the study was not designed with age as a primary focus, and therefore younger age was not foregrounded in the earlier sections of the manuscript.

However, the final sample comprised only younger adults, which may reflect limitations in the recruitment process. This became a defining feature of the dataset, and in turn, shaped the interpretation of the findings. We therefore considered this an important and unanticipated outcome, particularly given this represents a significant gap in the existing literature, as younger adults with coexisting physical and mental health long-term conditions remain underrepresented in self-management research. As such the prominence of younger participants with coexisting physical and mental health long-term conditions is thus presented as a novel contribution arising from the data rather than an a priori focus of the study.

In response to the reviewer’s comment, we have revised the Discussion section to improve balance across themes, while retaining appropriate emphasis on age as a key contextual and novel feature of the findings. We believe these revisions improve the coherence of the manuscript (Pages 29-34, Lines 643-659; 660-679, 697-701; 774-785).

Comment 3: Similarly, I feel that the conclusion of this manuscript (“The study found younger age to be a central inequality within self-management support, which crucially must be addressed…”) may be too strong given that older populations were not included in the study. While it is true that young people face unique barriers as described in the manuscript, they probably also have advantages older people do not possess. A more balanced interpretation might be more appropriate.

Response: We thank the reviewer for this important comment. We agree that the original concluding statement was stronger than warranted given the study did not include participants from older age groups and therefore cannot support direct comparisons across the life course. In response, we have revised the Conclusion in both the manuscript (Page 37, Lines 834-846) and abstract (Page 3, Lines 57-62) to provide a more balanced interpretation of the findings. The revised Conclusion now emphasises that, while our findings highlight age-related factors may influence self-management among emerging adults living with coexisting physical and mental health long-term conditions, further research is needed to examine how these experiences may differ across age groups. This revision better reflects the scope of the data while retaining the novel contribution of the study.

Comment 4: The provided link for data seems inaccessible (it says "DOI Not Found"). Please double check.

Response: We thank the reviewer for highlighting the issue. We have checked the link and confirm that the DOI is not currently resolving. A DOI has been reserved for this dataset, which is present in the updated Data Availability Statement, and will be formally minted by the University of Southampton Data Management Team upon acceptance of the manuscript. The DOI will resolve to the below URL once active.

The dataset has now been made publicly accessible via the University of Southampton repository. This dataset can be accessed at the following URL: https://eprints.soton.ac.uk/512196/

To provide further clarification, we have included a letter from the University’s Research Data Management team within the submission materials outlining this process. This ensures transparency regarding the status of the DOI and the associated data governance procedures.

Comment 5: Did the participants receive any compensation?

Response: We thank the reviewer for this question. The research study activities were unfunded, and as such participants did not receive any financial or material compensation for their participation. This has now been clearly stated in the revised Methods section of the manuscript (Page 13, Lines 307-308).

Comment 6: Line 109 - Explanation on “diagnostic overshadowing” might be beneficial for some readers

Response: We thank the reviewer for this helpful suggestion. We agree that a brief explanation of “diagnostic overshadowing” would improve clarity for readers. We have therefore added a concise definition in the revised manuscript (Page 5, Lines 107-108), describing diagnostic overshadowing as the tendency for symptoms of physical health conditions to be misattributed to an existing mental health diagnosis, potentially leading to under-recognition or delayed diagnosis of physical illness.

Comment 7: Line 178 - What criteria were used for anxiety/depression? Who diagnosed?

Response: We thank the reviewer for this important comment. Eligibility for anxiety and/or depression was based on participants’ self-reported diagnosis. This approach was used to enhance accessibility and inclusivity, recognising variability in access to formal diagnostic services and differences in diagnostic recording within NHS pathways. This has now been clarified in the revised Methods section (Page 9, Lines 198-200).

Comment 8: Line 585 – What is DSA?

Response: We thank the reviewer for this clarification comment. We have now expanded the acronym of DSA in brackets next to the quotation (Page 28, Lines 624-625). DSA refers to Disabled Students’ Allowance, a UK government-funded support scheme to help students in higher education who have a disability, long-term health condition, mental health condition, or specific learning difficulty. It is not a cash payment to the student, rather it provides funding for extra study-related support and equipment.

Additional Clarifications

In addition to the above comments, all spelling and grammatical errors pointed out by the reviewers have been corrected.

We look forward to hearing from you regarding our submission and to respond to any further questions and comments you may have.

Attachment Submitted filename: Response to Reviewers.docx

# Peer review: Decision Letter 1

**DOI:** 10.1371/journal.pone.0359157.r003

**Authors:** Ibrahim Jahun

28 Jul 2026

Dear Dr.  Lodwick,

plosone@plos.org

- A letter that responds to each point raised by the academic editor and reviewer(s). You should upload this letter as a separate file labeled 'Response to Reviewers'.
- A marked-up copy of your manuscript that highlights changes made to the original version. You should upload this as a separate file labeled 'Revised Manuscript with Track Changes'.
- An unmarked version of your revised paper without tracked changes. You should upload this as a separate file labeled 'Manuscript'.

If you would like to make changes to your financial disclosure, please include your updated statement in your cover letter. Guidelines for resubmitting your figure files are available below the reviewer comments at the end of this letter.

As the corresponding author, your ORCID iD is verified in the submission system and will appear in the published article. PLOS supports the use of ORCID, and we encourage all coauthors to register for an ORCID iD and use it as well. Please encourage your coauthors to verify their ORCID iD within the submission system before final acceptance, as unverified ORCID iDs will not appear in the published article. *Only*  the individual author can complete the verification step; PLOS staff *cannot*  verify ORCID iDs on behalf of authors.

We look forward to receiving your revised manuscript.

Kind regards,

Ibrahim Jahun, MD, MSC, PhD

Academic Editor

PLOS One

Journal Requirements:

1. If the reviewer comments include a recommendation to cite specific previously published works, please review and evaluate these publications to determine whether they are relevant and should be cited. There is no requirement to cite these works unless the editor has indicated otherwise.

2. Please review your reference list to ensure that it is complete and correct. If you have cited papers that have been retracted, please include the rationale for doing so in the manuscript text, or remove these references and replace them with relevant current references. Any changes to the reference list should be mentioned in the rebuttal letter that accompanies your revised manuscript. If you need to cite a retracted article, indicate the article’s retracted status in the References list and also include a citation and full reference for the retraction notice.

Additional Editor Comments:

Thank you for resubmitting the revised manuscript. Reviewer 2 has raised minor comments relating to their previous comments that you haven't fully addressed. Please review carefully these comments and address them accordingly.

[Note: HTML markup is below. Please do not edit.]

Reviewer’s Responses to Questions

**Comments to the Author**

Reviewer #1: (No Response)

Reviewer #2: (No Response)

**********

2. Is the manuscript technically sound, and do the data support the conclusions?

Reviewer #1: Yes

Reviewer #2: Yes

**********

3. Has the statistical analysis been performed appropriately and rigorously?

Reviewer #1: N/A

Reviewer #2: N/A

**********

4. Have the authors made all data underlying the findings in their manuscript fully available?

The PLOS Data policy

Reviewer #1: Yes

Reviewer #2: Yes

**********

5. Is the manuscript presented in an intelligible fashion and written in standard English?

Reviewer #1: Yes

Reviewer #2: Yes

**********

Reviewer #1: Thank you for undertaking this important study. Your team gleaned valuable insights from the SSIs, offering meaningful contributions to understanding the lived experiences of individuals managing mental health conditions alongside long‑term conditions. The findings highlight critical gaps that warrant attention in designing more inclusive and responsive healthcare systems.

I also appreciate the clear and detailed description of your analytic procedures. The discussion section was well‑structured, and the themes were logically connected, demonstrating strong coherence. I have provided a few minor recommendations, mainly related to punctuation, paraphrasing, and completing sentences, which are highlighted in the document. Overall, this is a thoughtful and original contribution to the field. Congratulations to the authors.

Reviewer #2: Thank you for your comprehensive revision. I believe the manuscript has now substantially improved.

I'd like to note a few minor additional points:

- Regarding my previous comment:

Comment 1: I felt the description of mental health conditions could be thicker. Which

mental health symptoms specifically affect self-management? How do they manage

their mental health conditions? Does psycho-/pharmacotherapy help?

My intention was more about whether your interview data provide insight into these issues. I don't feel very strongly about this, but please kindly add findings in this area if you have any. If not, please leave it as is. I also appreciate your added description in the Introduction.

- Regarding this comment and response:

Comment 7: Line 178 - What criteria were used for anxiety/depression? Who

diagnosed?

Response: We thank the reviewer for this important comment. Eligibility for anxiety

and/or depression was based on participants’ self-reported diagnosis. This approach

was used to enhance accessibility and inclusivity, recognising variability in access to

formal diagnostic services and differences in diagnostic recording within NHS

pathways. This has now been clarified in the revised Methods section (Page 9, Lines

198-200).

Thank you for the clarification. Is it self-report of diagnosis made by health workers, or does this include self-diagnosis? In other words, "I was diagnosed with depression (by a health worker)" or "(I think) I have depression"? I think this detail is important to know more accurately about the participant population. If the latter, it should probably be noted as a limitation, as it is harder to ascertain the nature of mental health conditions of the participants.

- Line 838: “person-cantered” - probably a typo

**********

what does this mean?). If published, this will include your full peer review and any attached files.

If you choose “no”, your identity will remain anonymous but your review may still be made public.

**Do you want your identity to be public for this peer review?**  For information about this choice, including consent withdrawal, please see our Privacy Policy

Reviewer #1: No

Reviewer #2: No

**********

[NOTE: If reviewer comments were submitted as an attachment file, they will be attached to this email and accessible via the submission site. Please log into your account, locate the manuscript record, and check for the action link "View Attachments". If this link does not appear, there are no attachment files.]

To ensure your figures meet our technical requirements, please review our figure guidelines: https://journals.plos.org/plosone/s/figures

You may also use PLOS’s free figure tool, NAAS, to help you prepare publication quality figures: https://journals.plos.org/plosone/s/figures#loc-tools-for-figure-preparation.

NAAS will assess whether your figures meet our technical requirements by comparing each figure against our figure specifications.

Attachment Submitted filename: PONE-D-26-09215_R1_ESM.pdf

# Author comment: Author response to Decision Letter 2

**DOI:** 10.1371/journal.pone.0359157.r004

9 Aug 2026

Comments from Reviewer 1

Comment 1: Line 61 - 'theneed' appearing in the online portal abstract

Response: We thank the reviewer for identifying this typo error. The term 'theneed' in the abstract on the online portal has been corrected to 'the need' (Page 3, Line 60).

Comment 2: Line 106 - Please delete this single quotation mark on "individuals'".

Response: We thank the reviewer for pointing this out. The single quotation mark has been deleted (Page 5, Line 106).

Comment 3: Line 112-114 - "Most recently observed..." Is this sentence complete?

Response: We thank the reviewer for highlighting this issue. The sentence has been revised for clarity and completeness (Page 5, Lines 112-114).

Comment 4: Line 153-156 - "Despite the recognition..." Do you think the full stop should be placed here? It makes the sentence read as incomplete.

Response: We thank the reviewer for this observation. We agree that the full stop resulted in an incomplete sentence. The text has been revised to ensure grammatical completeness and improve readability (Page 7, Lines 153-159).

Comment 5: Line 169 – To remove the 'a' before person-centred and contextually responsive.

Response: We thank the reviewer for pointing this out. The "a" has been removed to improve grammatical accuracy (Page 7, Line 169).

Comment 6: Line 170 - "self-management interventions" Do you think this needs to be in singular or plural form?

Response: We thank this reviewer for this helpful observation. We agree that the singular form is more appropriate in this context, this has now been rectified (Page 7, Line 170).

Comment 7: Line 326 - "allowing for more in-depth interpretation opposed to superficial understanding" Should this be as opposed to?

Response: We thank the reviewer for this suggestion. We agree that the phrase "as opposed to" provides a clearer and more grammatically complete comparison in this context. The text has been amended accordingly (Page 14, Line 326).

Comment 8: Line 741 - "MLTCs" I think this is the first time I am coming across this acronym. Can you please define this before using it? I see it appears only 2ce in the entire document...

Response: We thank the reviewer for this clarification comment. As the term appears only twice within the manuscript, the acronym has been removed, and multiple long-term conditions is now written in full throughout for improved clarity and readability (Page 34, Line 741).

Comment 9: Line 818 - should there be an inverted coma after the letter s' on "participants"

Response: We thank the reviewer for this comment. The apostrophe has now been corrected to "participants' lived experiences" (Page 38, Line 819).

Comment 10: Line 823-830 - should this be Secondly? Thirdly?

Response: We thank the reviewer for highlighting this inconsistency. The structure has now been revised to ensure consistency throughout the strengths and limitations paragraph (Page 38, Lines 824-837).

Comment 11: Line 838 - typo "person-cantered"

Response: We thank the reviewer for identifying this typo. The term "person-cantered" has been corrected to "person-centred" in the conclusion (Page 39, Line 842).

Comments from Reviewer 2

Comment 1: My intention was more about whether your interview data provide insight into these issues. I don't feel very strongly about this, but please kindly add findings in this area if you have any. If not, please leave it as is. I also appreciate your added description in the Introduction.

Response: We thank the reviewer for this important and insightful comment. We agree that this is a valuable consideration and has important implications for future research in this area. However, the current study did not specifically collect data relating to this aspect, as such we are unable to explore it further within the present manuscript. We have therefore retained the original text but will incorporate this consideration into the design of future studies in this area where it may help to further develop understanding of the topic.

Comment 2: Thank you for the clarification. Is it self-report of diagnosis made by health workers, or does this include self-diagnosis? In other words, "I was diagnosed with depression (by a health worker)" or "(I think) I have depression"? I think this detail is important to know more accurately about the participant population. If the latter, it should probably be noted as a limitation, as it is harder to ascertain the nature of mental health conditions of the participants.

Response: Thank you for this important observation. Participation eligibility was based on self-diagnosis/identification of a mental health condition. As such, participants may have either received a diagnosis from a healthcare professional or identified themselves as having anxiety and/or depression without formal diagnosis. We relied on participants self-reports and did not independently confirm diagnostic status. To improve clarity the terminology has been changed from self-reported to self-diagnosis (Page 9, Line 198), and this has now been acknowledged as a study limitation in the manuscript (Page 38, Line 831-834).

Comment 3: Line 838 - typo "person-cantered"

Response: We thank the reviewer for identifying this typo. The term "person-cantered" has been corrected to "person-centred" in the conclusion (Page 39, Line 842).

Attachment Submitted filename: Response_to_reviewers_auresp_2.docx

# Peer review: Decision Letter 2

**DOI:** 10.1371/journal.pone.0359157.r005

**Authors:** Ibrahim Jahun

11 Sep 2026

"I want to take the power back": An interpretative phenomenological analysis of self-management in coexisting physical and mental long-term conditions

PONE-D-26-09215R2

Dear Dr. Lodwick,

We’re pleased to inform you that your manuscript has been judged scientifically suitable for publication and will be formally accepted for publication once it meets all outstanding technical requirements.

Within one week, you’ll receive an e-mail detailing the required amendments. When these have been addressed, you’ll receive a formal acceptance letter and your manuscript will be scheduled for publication.

An invoice will be generated when your article is formally accepted. Please note, if your institution has a publishing partnership with PLOS and your article meets the relevant criteria, all or part of your publication costs will be covered. Please make sure your user information is up-to-date by logging into Editorial Manager at Editorial Manager® and clicking the ‘Update My Information' link at the top of the page. For questions related to billing, please contact billing support.

If your institution or institutions have a press office, please notify them about your upcoming paper to help maximize its impact. If they’ll be preparing press materials, please inform our press team as soon as possible -- no later than 48 hours after receiving the formal acceptance. Your manuscript will remain under strict press embargo until 2 pm Eastern Time on the date of publication. For more information, please contact onepress@plos.org.

Kind regards,

Ibrahim Jahun, MD, MSC, PhD

Academic Editor

PLOS One

Reviewers' comments:

Reviewer’s Responses to Questions

**Comments to the Author**

Reviewer #2: All comments have been addressed

**********

2. Is the manuscript technically sound, and do the data support the conclusions?

Reviewer #2: Yes

**********

3. Has the statistical analysis been performed appropriately and rigorously?

Reviewer #2: N/A

**********

4. Have the authors made all data underlying the findings in their manuscript fully available?

The PLOS Data policy

Reviewer #2: Yes

**********

5. Is the manuscript presented in an intelligible fashion and written in standard English?

Reviewer #2: Yes

**********

Reviewer #2: Thank you for your revision. I do not have additional comments or concerns, and I believe this manuscript is ready for publication.

**********

what does this mean?). If published, this will include your full peer review and any attached files.

If you choose “no”, your identity will remain anonymous but your review may still be made public.

**Do you want your identity to be public for this peer review?**  For information about this choice, including consent withdrawal, please see our Privacy Policy

Reviewer #2: **Yes:** Hiroshi Azuma

**********

# Editor report: Acceptance letter

**DOI:** 10.1371/journal.pone.0359157.r006

**Authors:** Ibrahim Jahun

PONE-D-26-09215R2

PLOS One

Dear Dr. Lodwick,

I'm pleased to inform you that your manuscript has been deemed suitable for publication in PLOS One. Congratulations! Your manuscript is now being handed over to our production team.

At this stage, our production department will prepare your paper for publication. This includes ensuring the following:

* All references, tables, and figures are properly cited

* All relevant supporting information is included in the manuscript submission,

* There are no issues that prevent the paper from being properly typeset

You will receive further instructions from the production team, including instructions on how to review your proof when it is ready. Please keep in mind that we are working through a large volume of accepted articles, so please give us a few days to review your paper and let you know the next and final steps.

Lastly, if your institution or institutions have a press office, please let them know about your upcoming paper now to help maximize its impact. If they'll be preparing press materials, please inform our press team within the next 48 hours. Your manuscript will remain under strict press embargo until 2 pm Eastern Time on the date of publication. For more information, please contact onepress@plos.org.

You will receive an invoice from PLOS for your publication fee after your manuscript has reached the completed accept phase. If you receive an email requesting payment before acceptance or for any other service, this may be a phishing scheme. Learn how to identify phishing emails and protect your accounts at https://explore.plos.org/phishing.

If we can help with anything else, please email us at customercare@plos.org.

Thank you for submitting your work to PLOS One and supporting open access.

Kind regards,

PLOS One Editorial Office Staff

on behalf of

Dr. Ibrahim Jahun

Academic Editor

PLOS One
